# Combined image and genomic analysis of high-grade serous ovarian cancer reveals PTEN loss as a common driver event and prognostic classifier

**DOI:** 10.1186/s13059-014-0526-8

**Published:** 2014-12-17

**Authors:** Filipe C Martins, Ines de Santiago, Anne Trinh, Jian Xian, Anne Guo, Karen Sayal, Mercedes Jimenez-Linan, Suha Deen, Kristy Driver, Marie Mack, Jennifer Aslop, Paul D Pharoah, Florian Markowetz, James D Brenton

**Affiliations:** Cancer Research UK Cambridge Institute, University of Cambridge, Li Ka Shing Centre, Robinson Way, Cambridge, CB2 0RE UK; Department of Obstetrics and Gynaecology, University of Cambridge, The Rosie Hospital, Robinson Way, Cambridge, CB2 0SW UK; Department of Pathology, Box 232, Addenbrooke’s Hospital, Hills Road, Cambridge, CB2 0QQ UK; National Institute for Health Research Cambridge Biomedical Research Centre, Cambridge, UK; Department of Pathology, Nottingham University Hospital, Nottingham, UK; Strangeways Research Laboratories, University of Cambridge, 2 Worts’ Causeway, Cambridge, CB1 8RN UK; Department of Oncology, University of Cambridge, Hutchison/MRC Research Centre, Hills Road, Cambridge, CB2 0XZ UK; Cambridge Experimental Cancer Medicine Centre, Cambridge, UK

## Abstract

**Background:**

*TP53* and *BRCA1/2* mutations are the main drivers in high-grade serous ovarian carcinoma (HGSOC). We hypothesise that combining tissue phenotypes from image analysis of tumour sections with genomic profiles could reveal other significant driver events.

**Results:**

Automatic estimates of stromal content combined with genomic analysis of TCGA HGSOC tumours show that stroma strongly biases estimates of *PTEN* expression. Tumour-specific PTEN expression was tested in two independent cohorts using tissue microarrays containing 521 cases of HGSOC. PTEN loss or downregulation occurred in 77% of the first cohort by immunofluorescence and 52% of the validation group by immunohistochemistry, and is associated with worse survival in a multivariate Cox-regression model adjusted for study site, age, stage and grade. Reanalysis of TCGA data shows that hemizygous loss of *PTEN* is common (36%) and expression of *PTEN* and expression of androgen receptor are positively associated. Low androgen receptor expression was associated with reduced survival in data from TCGA and immunohistochemical analysis of the first cohort.

**Conclusion:**

*PTEN* loss is a common event in HGSOC and defines a subgroup with significantly worse prognosis, suggesting the rational use of drugs to target PI3K and androgen receptor pathways for HGSOC. This work shows that integrative approaches combining tissue phenotypes from images with genomic analysis can resolve confounding effects of tissue heterogeneity and should be used to identify new drivers in other cancers.

**Electronic supplementary material:**

The online version of this article (doi:10.1186/s13059-014-0526-8) contains supplementary material, which is available to authorized users.

## Background

High-grade serous ovarian carcinoma (HGSOC) is the most common type of ovarian cancer and accounts for the majority of mortality from the disease. However, overall survival has been virtually unchanged since the introduction of platinum-based treatments [1]. HGSOC is characterised by ubiquitous mutation of *TP53* [2] and high prevalence of *BRCA1* and *BRCA2* germ-line mutations. With the exception of these genes, little is known about other prevalent driver events, and *BRCA1/2* and *PR* are the only robustly validated prognostic markers [3,4]. HGSOC has genomic similarities with basal-like breast tumours, which are also characterised by *TP53* and *BRCA1* alterations but additionally have *PTEN* loss [5–7]. Since *PTEN* loss is an important early initiating event in *BRCA1*-associated basal-like breast tumours [8], we hypothesised that it could also be a driver event in HGSOC.

PTEN is a phosphatase that inhibits cell proliferation induced by the PI3K pathway and acts as a tumour suppressor gene [9]. Targeted deletion of *PTEN* has been used to modulate the initiation of HGSOC and endometrioid ovarian cancer (EOC) in mouse models [10–13], but it is unknown whether *PTEN* loss could initiate or drive the progression of HGSOC in humans. The Cancer Genome Atlas (TCGA) study on genetic and epigenetic alterations in 489 cases of HGSOC confirmed *TP53* mutation and *BRCA1* downregulation as the main driver events and identified *PTEN* alterations in only 7% of tumours [4]. However, other immunohistochemistry-based studies in smaller cohorts found much higher frequencies of *PTEN* alterations, with loss of PTEN expression in 15% and partial loss in 50% to 60% of cases [14–16].

HGSOC has previously been stratified into distinct molecular subgroups based on gene-expression profiles: proliferative, differentiated, immunoreactive and mesenchymal [4,17,18]. However, the clinical utility of these classifiers is unclear, particularly as individual HGSOC samples may express multiple subtype signatures and the signatures show strong effects from stromal factors [18]. These signatures are likely to be driven by cell-autonomous effects such as *BRCA1* mutation (immunoreactive subtype) and the *Let-7* pathway (mesenchymal subtype) [19,20]. Identification of other dominant cell-autonomous drivers therefore requires deconvolution of stromal signatures from those of carcinoma cells. Joint analysis of tissue images and genomic profiles has only recently been introduced to study these effects, and reveals information that cannot be attained from genomic data alone [21].

We hypothesised that *PTEN* loss might be more frequent than observed in the TCGA data set owing to confounding by samples with high stromal content. Here, we have developed bioinformatic and image analysis methods to correct gene expression signatures in the TCGA HGSOC data and tested these predictions in two independent cohorts of HGSOC cases.

## Results

### Estimation of *PTEN* expression in high-grade serous ovarian carcinoma is strongly influenced by stromal content

**Figure 1:**
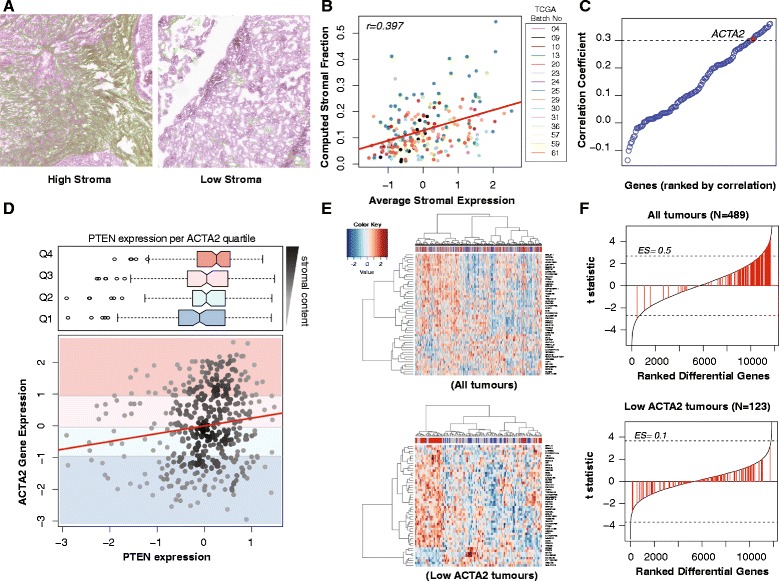
**PTEN expression in TCGA samples correlates with ACTA2 expression, and thus stromal content.** **(A)** Example of H&E stained sections from TCGA samples having low and high stromal content. The stromal content detected using the segmentation algorithm is shown in green. **(B)** Average expression of combined stromal signature correlated well with automated quantification in (A) (*r*=0.397, *N*=216 patients). **(C)** Univariate correlation testing of stromal genes from Yoshihara with image analysis showing that ACTA2 is highly correlated with stromal content (*r*=0.306) [22]. **(D)** Scatter plot showing the distribution of PTEN vs ACTA2 mRNA expression for each sample (Pearson correlation, *r*=0.152; *P*<0.001) with corresponding box plots showing distribution of PTEN expression in different ACTA2 expression quartiles. There is higher PTEN expression with high ACTA2 expression (Jonchkeere Terpsa test, *P*=0.001). **(E)** Heat maps of top 50 differentially expressed genes between PTEN^high^ and PTEN^low^ tumours in the TCGA data set before and after selecting for ACTA2 low samples. In each case, the top quartile of PTEN expression was labelled PTEN^high^, and the lowest quartile PTEN^low^. Correction for ACTA2 content reveals *AR* as one of the top differentially expressed genes. **(F)** Stromal gene set enrichment plots after differential expression analysis between high and low PTEN. Stromal-related genes from the Yoshihara signature (141 genes, highlighted in red) are redistributed [22]. There is less enrichment for stromal-related genes after correcting for stroma content (enrichment score 0.5 to 0.1). Dotted lines indicate adjusted *P*=0.05.

We evaluated the stromal content of 216 HGSOC samples from TCGA in a total of 302 images using a computational framework validated through scoring by an independent observer (Jonckheere–Terpstra test for trend *P*=0.001) (Figure 1A and Additional file 1: Figure S1). The automated stromal scores were highly correlated with the expression of genes from a published stromal gene signature (Figure 1B) [22]. *ACTA2* ranked 17 in the top correlated stromal genes and was therefore selected for subsequent analysis on the basis of its known stromal-specific expression (Figure 1C) [23].

High *ACTA2* expression in the TCGA samples was directly correlated with *PTEN* expression and was never associated with low *PTEN* values, suggesting that in the majority of samples it was stromal *PTEN* expression that was being measured (Figure 1D).

Differential gene analysis comparing the upper and the lower quartiles of *PTEN* expression showed enrichment for stromal genes in tumours with high *PTEN* (Gene Set Enrichment Analysis (GSEA) Enrichment Score = 0.5). However, performing the analysis on samples with low *ACTA2* content (the first quartile) showed a more random distribution of stromal genes (GSEA ES=0.1), suggesting this subset is less influenced by stromal content (Figure 1E,F). The wider distribution of *PTEN* expression in quartile one of *ACTA2* expression also supports the hypothesis of tumour *PTEN* loss being more prevalent than previously estimated (Figure 1D).

### *PTEN* loss is prevalent and has prognostic value in high-grade serous ovarian carcinoma

**Figure 2:**
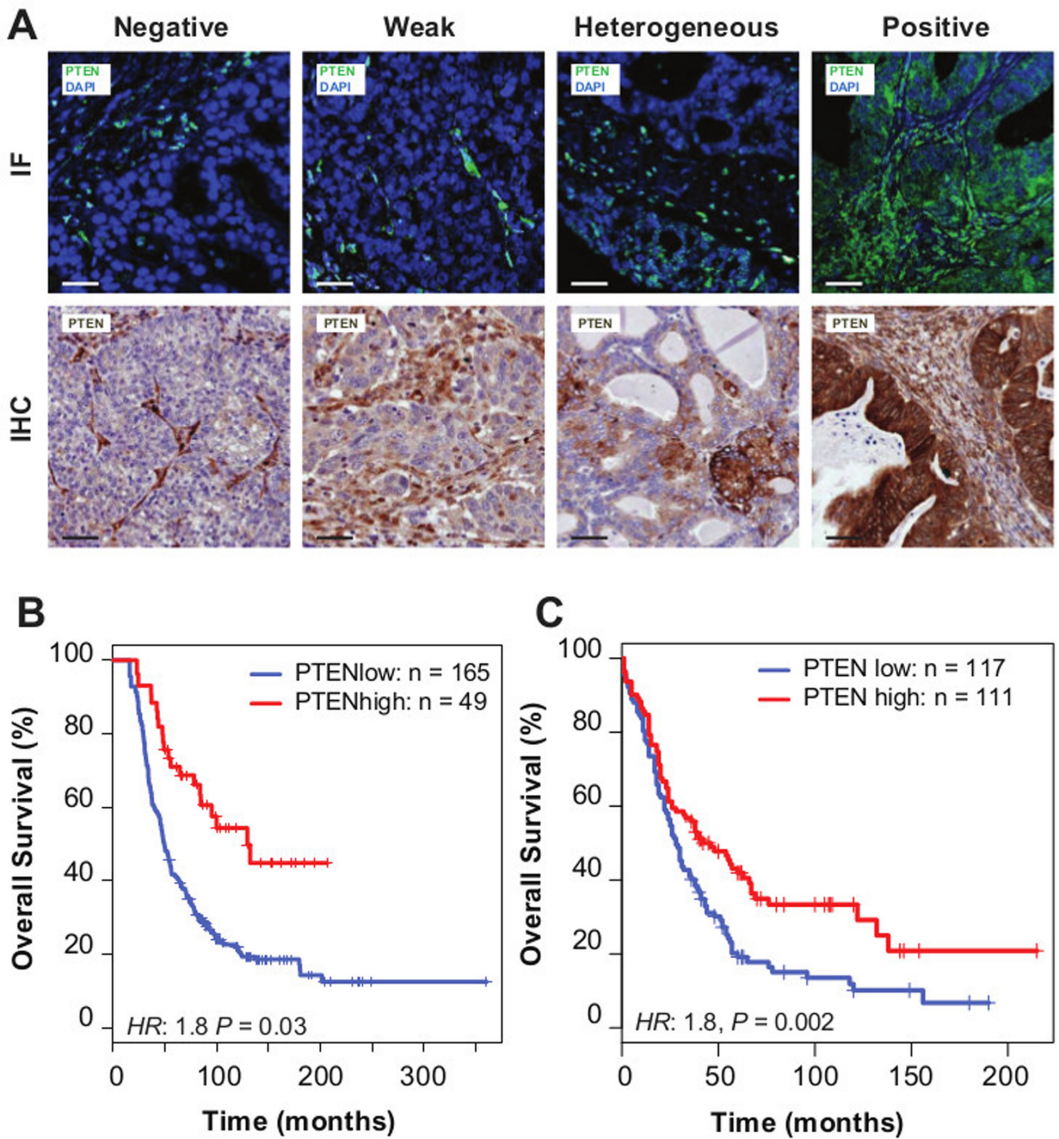
**PTEN loss is highly prevalent in HGSOC and is associated with poor prognosis.** **(A)** Examples of immunofluorescence (IF) and immunohistochemistry (IHC) staining in ovarian cancer samples for scoring of PTEN expression. **(B)** Kaplan–Meier survival curves for PTEN positive vs PTEN negative, weak or heterogeneous staining for HGSOC from the SEARCH study (using IF) (multivariate hazard ratio 1.8, 95% CI 1.0 to 3.0, *P*=0.03) and **(C)** Kaplan–Meier survival curves for PTEN positive vs PTEN negative, weak or heterogeneous stainings for HGSOC from the Nottingham Ovarian Cancer Study (using IHC) (multivariate hazard ratio 1.8, 95% CI 1.2 to 2.6, *N*=228, *P*=0.002). CI, confidence interval; IF, immunofluorescence; IHC, immunohistochemistry.

To test the predictions that reduced *PTEN* expression could be a frequent event, we developed methods to quantify tumour-specific PTEN expression using a semi-quantitative immunofluorescence (IF) procedure. We applied this to tissue microarrays constructed from the population-based Study of Epidemiology and Risk Factors in Cancer Heredity (SEARCH) cohort (*N*=245 HGSOCs from 516 ovarian cancer samples; Table 1) [3]. For HGSOC, PTEN expression was variable and showed a range of intensities, from negative (23*%*;*N*=49), weak positive (32*%*;*N*=68) to strongly positive fluorescence (23*%*;*N*=49). Heterogeneous fluorescence was also observed in 48 samples, 22% of the cases (Figure 2A).

**Table 1:** **Summary of SEARCH cohort**

		**HGSOC**	**LGSOC**	**MOC**	**EOC**	**CCOC**	**Others**
Number of patients		245	35	50	97	62	27
Age at diagnosis (years)		57 (8.2)	53.9 (11.8)	53.4 (12.4)	54.5 (9.4)	55.8 (7.4)	56.2 (9)
Entry time (months)		23.5 (18.9 to 32.1)	26.4 (19.6 to 33.5)	28 (18.2 to 47.6)	28.7 (21.2 to 46.4)	29.4 (19.4 to 59.6)	27.8 (23.1 to 31.6)
Last follow-up time (months)		71.1 (45.4 to 105.6)	126.5 (85.7 to 149.1)	123 (83.1 to 170.3)	140.9 (86.5 to 171.2)	144.8 (101.6 to 208.2)	97.8 (73.7 to 139.7)
Vital status	Data available	245 (100%)	35 (100%)	50 (100%)	97 (100%)	62 (100%)	27 (100%)
	Alive	89 (36%)	27 (77%)	39 (78%)	69 (71%)	47 (76%)	16 (59%)
	Died	156 (64%)	8 (23%)	11 (22%)	28 (29%)	15 (24%)	11 (41%)
Stage	Data available	238 (97%)	33 (94%)	47 (94%)	96 (99%)	62 (100%)	24 (89%)
	I	46 (19%)	27 (82%)	42 (89%)	76 (79%)	50 (81%)	13 (54%)
	II	27 (11%)	2 (6%)	1 (2%)	13 (14%)	6 (10%)	5 (21%)
	III	139 (58%)	4 (12%)	4 (9%)	6 (6%)	5 (8%)	5 (21%)
	IV	26 (11%)	0 (0%)	0 (0%)	1 (1%)	1 (2%)	1 (4%)
Grade	Data available	201 (82%)	13 (37%)	26 (52%)	78 (80%)	26 (42%)	11 (41%)
	I	1 (0%)	11 (85%)	10 (38%)	22 (28%)	2 (8%)	1 (9%)
	II	56 (28%)	1 (8%)	14 (54%)	40 (51%)	11 (42%)	7 (64%)
	III	144 (72%)	1 (8%)	2 (8%)	16 (21%)	13 (50%)	3 (27%)
PTEN status (IF)	Data available	214 (87%)	29 (83%)	41 (82%)	89 (92%)	59 (95%)	26 (96%)
	Negative	49 (23%)	2 (7%)	12 (29%)	39 (44%)	34 (58%)	8 (31%)
	Weak positive	68 (32%)	6 (21%)	11 (27%)	27 (30%)	18 (31%)	8 (31%)
	Heterogeneous	48 (22%)	7 (24%)	2 (5%)	3 (3%)	1 (2%)	3 (12%)
	Positive	49 (23%)	14 (48%)	16 (39%)	20 (22%)	6 (10%)	7 (27%)
PTEN status (IHC)	Data available	211 (86%)	28 (80%)	44 (88%)	90 (93%)	56 (90%)	26 (96%)
	Negative	53 (25%)	3 (11%)	9 (20%)	35 (39%)	22 (39%)	4 (15%)
	Weak positive	73 (35%)	8 (29%)	5 (11%)	28 (31%)	18 (32%)	5 (19%)
	Heterogeneous	34 (16%)	3 (11%)	6 (14%)	6 (7%)	3 (5%)	3 (12%)
	Positive	51 (24%)	14 (50%)	24 (55%)	21 (23%)	13 (23%)	14 (54%)

Data are mean (SD), median (OQR), *n* (%) or *n*.

CCOC, clear cell ovarian cancer; EOC, endometrial ovarian cancer; HGSOC, high-grade serous ovarian cancer; IF, immunofluorescence; IHC, immunohistochemistry; LGSOC, low-grade serous ovarian cancer; MOC, mucinous ovarian cancer.

Reduced PTEN fluorescence (including negative, weak or heterogeneous expression) was associated with significantly worse survival for HGSOC compared with positive fluorescence, independent of study site, age, stage and grade (hazard ratio 1.8, 95% confidence interval (CI) 1.0 to 3.0, *P*=0.03; Figure 2B, Additional file 2: Table S1).

Comparison of immunohistochemistry (IHC) for PTEN to the immunofluorescent assay showed strong correlation (*P*≪0.001; Additional file 3: Figure S2A). We therefore used IHC to extend the initial analysis in an independent validation cohort of incident ovarian cancer cases (*N*=276 HGSOC cases from 507 ovarian cancer samples; Table 2). Reduced PTEN expression was associated with significantly worse survival for HGSOC compared with positive expression, independent of study site, age, stage and grade (hazard ratio 1.8, 95% CI 1.2 to 2.6, *N*=228, *P*=0.002) (Figure 2C, Additional file 2: Table S2). Combined analysis of both data sets was associated with a multivariate hazard ratio 1.5 (95% CI 1.1 to 2.0, *N*=439, *P*=0.006; Additional file 3: Figure S2B) for reduced *PTEN* expression.

**Table 2:** **Summary of Nottingham cohort**

		**HGSOC**	**LGSOC**	**MOC**	**EOC**	**CCOC**	**Others**
Number of patients		276	40	54	62	50	24
Age at diagnosis (years)		62.6 (10.7)	54.4 (16)	56.6 (17.8)	60.8 (11.8)	59.4 (12.2)	62 (12.2)
Overall survival (months)		34 (17 to 60)	80 (50 to 107.2)	63 (16 to 108)	90 (59 to 120)	52.5 (30.5 to 108.8)	44 (2 to 88.5)
Vital status	Data available	272 (99%)	38 (95%)	53 (98%)	61 (98%)	48 (96%)	23 (96%)
	Alive	69 (25%)	30 (79%)	26 (49%)	36 (59%)	19 (40%)	11 (48%)
	Died	203 (75%)	8 (21%)	27 (51%)	25 (41%)	29 (60%)	12 (52%)
Stage	Data available	270 (98%)	40 (100%)	52 (96%)	62 (100%)	48 (96%)	24 (100%)
	I	41 (15%)	24 (60%)	42 (81%)	36 (58%)	28 (58%)	8 (33%)
	II	31 (11%)	3 (8%)	1 (2%)	13 (21%)	8 (17%)	1 (4%)
	III	168 (62%)	12 (30%)	7 (13%)	11 (18%)	12 (25%)	12 (50%)
	IV	30 (11%)	1 (2%)	2 (4%)	2 (3%)	0 (0%)	3 (12%)
Grade	Data available	197 (71%)	21 (52%)	39 (72%)	60 (97%)	38 (76%)	10 (42%)
	I	1 (1%)	20 (95%)	18 (46%)	10 (17%)	0 (0%)	2 (20%)
	II	4 (2%)	0 (0%)	15 (38%)	17 (28%)	0 (0%)	1 (10%)
	III	192 (97%)	1 (5%)	6 (15%)	33 (55%)	38 (100%)	7 (70%)
PTEN status (IHC)	Data available	233 (84%)	34 (85%)	40 (74%)	55 (89%)	43 (86%)	23 (96%)
	Negative	27 (12%)	1 (3%)	4 (10%)	18 (33%)	14 (33%)	5 (22%)
	Weak positive	65 (28%)	5 (15%)	17 (42%)	13 (24%)	17 (40%)	6 (26%)
	Heterogeneous	29 (12%)	0 (0%)	3 (8%)	0 (0%)	1 (2%)	5 (22%)
	Positive	112 (48%)	28 (82%)	16 (40%)	24 (44%)	11 (26%)	7 (30%)

Data are mean (SD), median (OQR), *n* (%) or *n*.

CCOC, clear cell ovarian cancer; EOC, endometrial ovarian cancer; HGSOC, high-grade serous ovarian cancer; IF, immunofluorescence; IHC, immunohistochemistry; LGSOC, low-grade serous ovarian cancer; MOC, mucinous ovarian cancer.

We examined for interactions between *BRCA1* and PTEN loss of expression as *PTEN* loss is a frequent initiating event in *BRCA1*-associated breast tumours and is associated with basal-like breast cancer [8]. All patients with a deleterious germ-line *BRCA1* mutation had HGSOC tumours and were marginally more likely to have negative or weak PTEN staining (Fisher’s exact test *P*=0.06, *N*=9/10 and Additional file 2: Table S1).

### *PTEN* is frequently deleted in high-grade serous ovarian carcinoma

**Figure 3:**
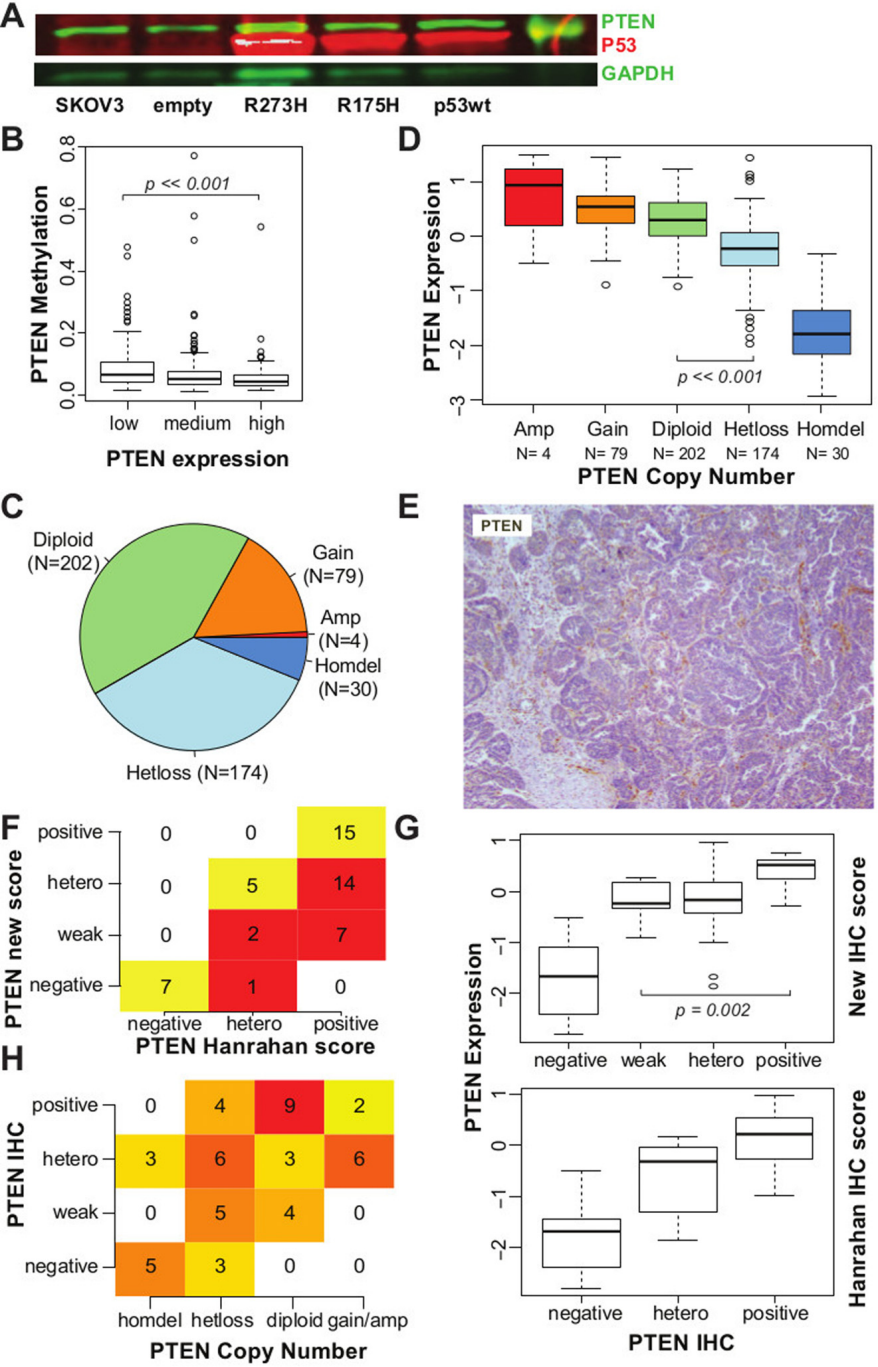
**PTEN loss in HGSOC mainly occurs through copy number alteration (CNA).** **(A)** Western blot showing the quantification of PTEN, TP53 and GAPDH (loading control) from extracts of the ovarian cancer SKOV3 cell line and complemented with wild-type and mutated TP53. **(B)** Box plot showing distribution of methylation levels according to PTEN expression levels in TCGA samples. A small increase in DNA methylation is observed in samples with lower PTEN expression (Wilcoxon test *P*≪0.001). **(C)** Pie chart showing distribution of PTEN ploidy within the TCGA data set. **(D)** Box plot showing distribution of PTEN mRNA expression according to PTEN ploidy, suggesting that CNA influences mRNA expression (*t*-test, diploid vs hetloss *P*≪0.001). **(E)** Example of IHC staining for PTEN scored as positive in [14], but reclassified here as weak staining, as staining in tumour cells is markedly reduced in comparison to stromal cells. **(F)** Table showing differences between scoring for PTEN IHC staining used [14] and reclassified score. Of the original 36 positive samples, 21 have been reclassified as heterogeneous or weakly positive. **(G)** Box plot showing distribution of PTEN mRNA expression within each scoring group for PTEN IHC staining, according to the Hanrahan score [14] and the reclassified score. There is a significant difference between PTEN expression in tumours classified as weak positives and positives (*t*-test, *P*=0.002). **(H)** Contingency table showing significant correlation between scores for PTEN IHC staining and PTEN CNA (Fisher’s exact test, *P*≪0.001). CNA, copy number alteration; IF, immunofluorescence; GAPDH, glyceraldehyde 3-phosphate dehydrogenase; hetero, heterogeneous; IHC, immunohistochemistry; TCGA, The Cancer Genome Atlas.

*TP53* is mutated in more than 95% of HGSOC cases [2,4] and has been previously implicated in controlling *PTEN* transcription [24]. We tested for *TP53* effects on *PTEN* expression by introducing a bacterial artificial chromosome transgene containing the entire human *TP53* locus into the *TP53*-null cell line SKOV3. *PTEN* expression was independent of *TP53* complementation for both wild-type and mutated (R175H, R273H) transgenes (Figure 3A). Comparison of promoter methylation and expression of *PTEN* in the TCGA data set showed infrequent methylation of the *PTEN* promoter region in low-*PTEN* expressing cases (Figure 3B).

By contrast, loss of a single *PTEN* allele was common in HGSOC (36%; *N*=174) in addition to the previously described homozygous deletion occurring in 6% of tumours [4] (Figure 3C). *PTEN* gene expression was significantly lower when there was loss of at least one allele (Figure 3D; *t*-test, *P*≪0.001). To assess whether protein expression of PTEN was associated with copy number, we applied our IHC staining classification to a subgroup of 51 samples from the TCGA cohort that has been recently stained for PTEN [14]. A large number of positive samples were reclassified as weak positive (Figure 3E,F and Additional file 2: Table S3). These tumours had similar levels of *PTEN* mRNA to those with heterogeneous staining, and significantly lower levels than tumours with positive staining (Wilcoxon test, *P*=0.002; Figure 3G), emphasising the importance of differentiating intensities in PTEN staining. Negative staining was strongly correlated with homozygous deletion, and weak or heterogeneous staining with hemizygous loss; positive staining was associated with no chromosomal loss (Fisher’s exact test, *P*=0.001; Figure 3H).

### Androgen receptor expression is associated with *PTEN* expression

We analysed *PTEN* differentially expressed genes for TCGA samples with low *ACTA2* content to mitigate the effect of stromal contamination. *AR* was one of the top differentially expressed genes (Figure 1E), and this was confirmed using an orthogonal method, csSAM, which takes into account stromal content from H&E images (Additional file 4).

**Figure 4:**
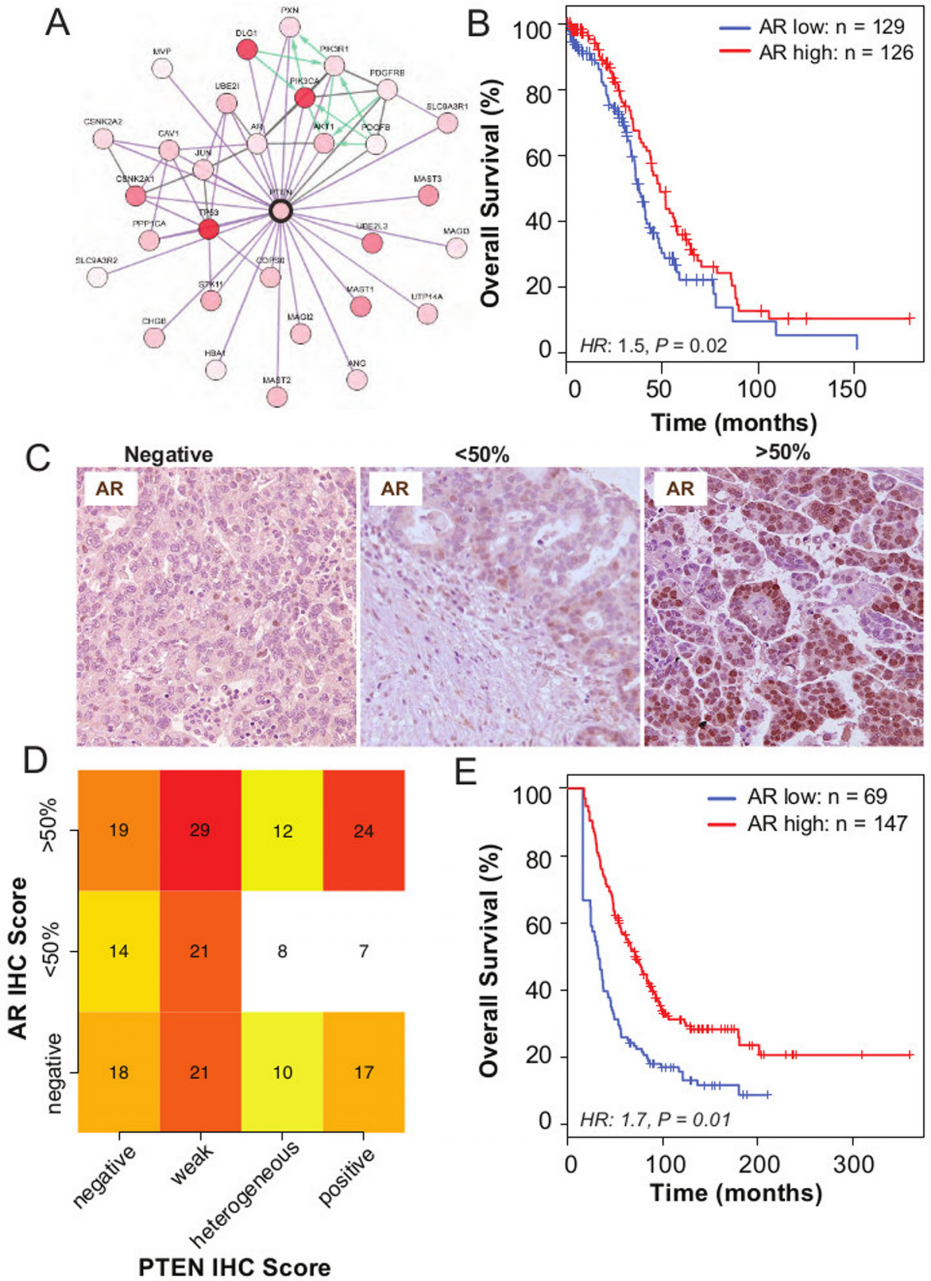
**Androgen receptor is co-regulated with** ***PTEN*** **and is associated with good prognosis.** **(A)** Protein–protein interaction network of proteins associated with high PTEN expression shows co-expression of *AR* and *PTEN* (figure obtained from cBioPortal website using the provisional TCGA data for ovarian serous cystadenocarcinoma). **(B)** Kaplan–Meier survival curves for AR expression show prognostic value, after dichotomising AR RNA-sequencing expression levels at the median level (*N*_high_=126, *N*_low_=129; hazard ratio 1.5, 95% CI 1.1 to 2.1, *P*=0.02). **(C)** Examples of immunohistochemistry (IHC) AR staining of HGSOC samples. **(D)** Heat map shows no statistical association between AR and PTEN IHC expression in HGSOC samples from the SEARCH cohort (chi-squared test, *P*=0.63). **(E)** Kaplan–Meier survival curves for AR positive vs AR negative for HGSOC from the SEARCH study (using IHC) (multivariate hazard ratio 1.7, 95% CI 1.1 to 2.4, *P*=0.01). CI, confidence interval; HGSOC, high-grade serous ovarian carcinoma; HR, hazard ratio; IF, immunofluorescence; IHC, immunohistochemistry; SEARCH, Study of Epidemiology and Risk Factors in Cancer Heredity.

Protein–protein interaction data from TCGA available through cBioPortal [25] showed that the highly ranked phosphorylated proteins in the lower quartile of *PTEN* expression (defined as *PTEN* RNA-sequencing expression, *z* score<−0.5) included AKT1, AKT2 and AKT3, which reflects activation of the PI3K pathway. Additionally, another highly ranked protein expressed in this subgroup was AR. A direct link between AR and PTEN was suggested by overlaying genomic information, including copy number and gene expression, on a known protein interaction network (Figure 4A). Moreover, *AR* expression has also been associated with *PTEN* expression in prostate cancer [26]. Therefore, we hypothesised that *AR* expression was prognostically significant. Using the TCGA RNA-sequencing data, we found that low AR expression was associated with shorter overall survival (hazard ratio 1.5, 95% CI 1.1 to 2.1, *P*=0.02; Figure 4B).

These results were validated experimentally in 216 samples from the SEARCH cohort. It was found that 43%, 25% and 32% of HGSOC expressed high levels of AR (≥50% of tumour cells), low levels of AR (<50% of tumour cells) or no AR, respectively (Figure 4C). In a multivariate analysis, we found a similar prognostic effect for expression in these samples (hazard ratio 1.7, 95% CI 1.1 to 2.4, *P*=0.01), despite there being no strong association between AR and PTEN (chi-squared test *P*=0.63; Figure 4D,E).

### Differentiated and proliferative expression subtypes are associated with high and low *PTEN* expression

**Figure 5:**
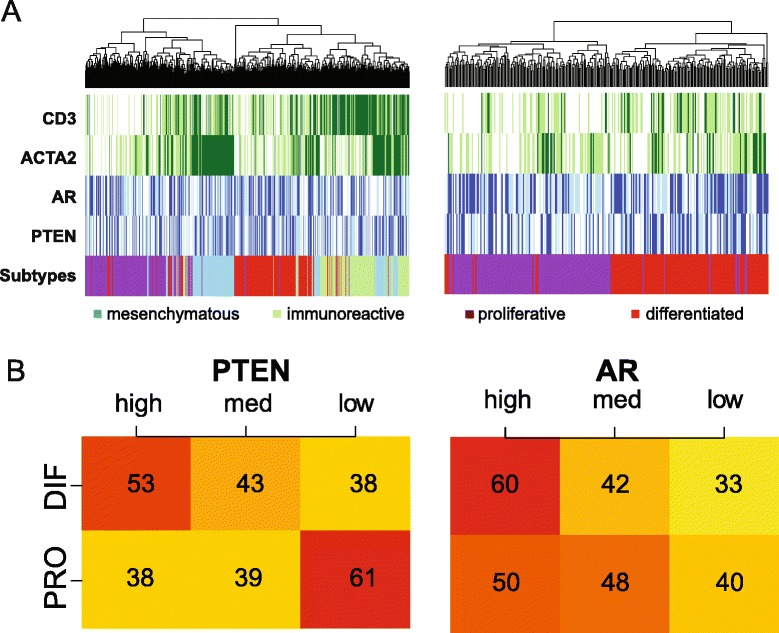
**PTEN** ^**high**^ **AR** ^**high**^ **and PTEN** ^**low**^ **AR** ^**low**^ **tumours correlate with the differentiated and proliferative subgroups.** **(A)** Heat maps representing the association between HGSOC TCGA subtypes and expression of CD3, ACTA2, AR and PTEN in the TCGA data set. Mesenchymal and immunoreactive subtypes show good correlation with ACTA2 and CD3 expression, respectively (left). The heat map on the right shows only the proliferative and differentiated subtypes and suggests that high AR and PTEN expression is associated with the differentiated subtype. **(B)** Categorisation of patients into high, medium and low expression for PTEN and AR shows an association between PTEN expression and the differentiated and proliferative subtypes (chi-squared test, PTEN *P*=0.02 and AR *P*=0.38). DIF, differentiated; HGSOC, high-grade serous ovarian carcinoma; med, medium; PRO, proliferative; TCGA, The Cancer Genome Atlas.

TCGA subdivided HGSOC into four subgroups based on gene expression profiles, differentiated, proliferative, mesenchymal and immunoreactive, in which the latter two were enriched for stromal cells and leukocytes (Figure 5A) [4,18].

*PTEN* loss is associated with activation of the PI3K pathway and consequently with proliferation. Therefore, we hypothesised that *PTEN* expression could be associated with the remaining differentiated and proliferative subgroups. By focusing on these two subgroups and categorising the raw *AR* and *PTEN* expression into tertiles, higher *PTEN* expression was associated with the differentiated subgroup (Figure 5B, chi-squared test *P*=0.02), whilst lower *PTEN* expression was associated with the proliferative subgroup.

## Discussion

In this work, we have developed and validated image analysis methods to score stromal components in tumour images automatically. We used these methods to identify tissue samples with low stromal content from TCGA, which allowed us to examine the range of *PTEN* RNA expression accurately, confirming that *PTEN* expression in HGSOC was highly variable. These observations supported our hypothesis that *PTEN* loss could be common in HGSOC and was confirmed using a validated *PTEN* antibody on tissue microarrays from two independent clinical cohorts.

Extensive genomic analysis of HGSOC has revealed common involvement of tumour suppressor genes, including *TP53*, *BRCA1* and *BRCA2*, but only rare involvement of ‘actionable’ oncogenic mutations. The importance of identifying driver mutations in cancer and using them for therapeutic targets has been extensively demonstrated and a robust molecular stratification of HGSOC is required to predict prognosis accurately and to support rational drug development for this disease. Previous genomic analysis of HGSOC has identified four molecular subtypes of HGSOC based on gene expression profiles: differentiated, proliferative, mesenchymal and immunoreactive [4,17,18]. However, these profiles have only weak prognostic value, are highly influenced by stromal contribution and do not have direct therapeutic implications. Cellular heterogeneity in tumour samples is a common confounder for genomic analysis and it is important to note that the spatial distribution and number of normal and tumour cells can provide important phenotypes that are not represented in genomic profiles [21,27].

In this work, by using image analysis to focus on tumour-cell-specific PTEN expression, we have shown that *PTEN* loss is a common event in HGSOC, supporting previous IHC-based studies [14–16]. Although there is conflicting data on the prognostic value of *PTEN* [16,28], in our analysis of 442 HGSOC cases from two separate cohorts, we have demonstrated that *PTEN* loss or downregulation is prognostic and is strongly associated with worse overall survival. Positive PTEN staining has comparable survival effects to those described for *BRCA1/2* carriers [4]. TCGA previously demonstrated homozygous deletion of *PTEN* in 6% of HGSOC cases [4], and our reanalysis of this data set additionally shows that heterozygous loss is common in tumour cells (36%) and is associated with reduced expression of *PTEN* RNA and protein.

As methylation of *PTEN* was only infrequently observed in the TCGA data, other post-transcriptional modifications may play an important role in regulating PTEN [29]. PTEN is regulated by a complex network of miRNAs and mRNAs that share the same miRNA binding site – these have been termed competing endogeneous RNA (ceRNA). Also, the *PTEN* pseudogene *PTENP1* can indirectly regulate *PTEN* expression [30]. Further studies will be needed to clarify whether the heterogeneous expression patterns observed can be explained by these post-transcriptional modifications, tumour heterogeneity or upstream genetic changes.

PTEN gene dosage is finely regulated and small expression changes may have important phenotypic effects [31]. In our analysis, weak expression of PTEN had similar detrimental effects on survival as total loss of expression. These survival effects are consistent with models suggesting haploinsufficiency phenotypes for *PTEN* [8,32]. Previous analyses of early serous tubal carcinomas showed that 33% (*N*=4/12) of cases had complete loss of PTEN expression and a further 33% had heterogeneous loss [15]. Together with our observations, these data strongly support the contention that *PTEN* is a prevalent early driver event in HGSOC. This is further supported by data from mouse models where the addition of *PTEN* deletion to alteration of *DICER*, or *TP53* and *BRCA1*, was critical for initiation and progression of HGSOC [10,11].

In breast cancer, *PTEN* loss is more prevalent in association with *BRCA1* germ-line mutations, and tumour analysis at the single-cell level has shown that it is the most common initiating event in *BRCA1*-associated breast tumours [7,8]. Owing to our small sample size, our results suggest, but do not confirm, that *PTEN* loss is more prevalent in *BRCA1*-associated HGSOC.

We also show that the *PTEN*^high^ subgroup with improved outcome was associated with the differentiated expression signature, whereas the poor prognosis *PTEN*^low^ subgroup was associated with the proliferative signature. The stratification of HGSOC patients into *PTEN*^high^ and *PTEN*^low^ subgroups may have important therapeutic implications. Firstly, *PTEN* loss activates the PI3K pathway and tumours that present reduced expression of *PTEN* may respond to PI3K inhibitors. Additionally, *PTEN* loss has also been associated with response to PARP inhibitors in endometrial cell lines [33] and a mechanistic basis for this has been suggested by recent findings that implicate nuclear PTEN in the regulation of homologous recombination [34]. Recent data suggest strong activity for compound PARP-PI3K inhibition in prostate and breast cancers and these drugs may be effective for the PTEN^low^ HGSOC subgroup [35,36].

The important association between *AR* and *PTEN* has previously been demonstrated for prostate cancer but not tested for ovarian cancer [26]. By correcting gene signatures from TCGA for stromal content, we showed that *AR* is co-expressed with *PTEN* and higher levels of *AR* expression were associated with longer overall survival. This is consistent with a recent meta-analysis that also suggested a better overall survival for patients with breast tumours with higher androgen receptor (AR) expression, independent of Estrogen Receptor (ER), and new clinical trials targeting AR in breast cancer have already been put in place [37]. However, our tissue microarray data showed a weaker correlation between PTEN and AR protein expression than observed in the genomic data. This may reflect the possible role of post-transcriptional modifications on protein expression, and the large degree of intra-tumour heterogeneity observed in IHC staining. Clinical trials with AR antagonists in ovarian cancer performed over 20 years ago suggested that only a small subset of tumours may respond to these drugs [38]. Our results suggest that stratification based on AR expression may allow for the identification of a potentially responsive high AR subset, which is associated with better prognosis.

## Conclusions

*PTEN* loss, together with *TP53* and *BRCA1* alterations, is a common event in HGSOC and, in combination with *AR*, allows for a prognostic stratification of HGSOC subgroups, which may be amenable to targeted therapies. We have demonstrated that important genomic events that are confounded by stromal contribution in tumour samples, such as PTEN loss, can be resolved by integrating image analysis with protein and gene expression. Such bioinformatic approaches are broadly applicable and could lead to important discoveries in other diseases where heterogeneity of the tissue may be a confounding issue in genomic analyses.

## Materials and methods

### Overview of data sets used

Analyses were performed on three data sets:

1. The publicly available TCGA, from which H&E images, mRNA expression, genomic data including copy number variation and DNA methylation data, and corresponding clinical information for 489 HGSOC patient samples were obtained. This was downloaded from the TCGA data portal [4] and cBioPortal [25]. A total of 51 IHC samples for PTEN from this data set were obtained from Hanrahan *et al.* [14] and used to correlate genomic information with protein expression [14].
2. SEARCH data set with tissue samples and corresponding clinical information for 245 HGSOC samples (out of 516 ovarian cancer cases; Table 1). Patients were recruited after a diagnosis of ovarian cancer and if they were able to consent for participation in the study [3]. Key demographical and clinical data on the patients, including *BRCA1* germ-line mutation status, were presented anonymously.
3. Nottingham Ovarian Cancer Study (NOT) data set with tissue samples and corresponding clinical data from 276 HGSOC samples (out of 507 ovarian cancer cases; Table 2). This is a retrospective study of ovarian cancer cases diagnosed between 1991 and 2011 [3]. For this study, the institutional research ethics boards (East Of England Cambridgeshire REC (for SEARCH) and Derbyshire REC (for NOT)) waived the need to obtain consent. Both local human research investigation committees approved each study.

### Correcting gene expression profiles using image analysis

Scoring by eye for overall image quality (based on discolouration, folding over of the mounted section and completeness of the section) was performed on 312 slides, of which 46 were discarded owing to poor quality, leaving 266 slides from 194 patients. Based on quality, 302 H&E slides from 216 patient samples in the TCGA database were selected, of which an observer (FCM) manually scored 266 for stromal content. Images were segmented by first applying an entropy filter to remove the background from an image, followed by colour deconvolution according to Ruifroks’ method (Additional file 5) [39]. The haematoxylin channel was subtracted from the eosin channel, leaving a raw stromal signal. Otsu’s thresholding and smoothing was then performed to estimate a stromal fraction [40]. These values along with stromal gene scores were used to predict the stromal content in the remaining TCGA samples. Correlation between automated and manual scoring was performed using the Jonckheere–Terpstra test for trend. Gene-expression-based validation of the method was performed by generating a stromal gene list [22] and performing univariate Pearson’s correlation testing between stromal quantification and expression.

### Differential gene expression analysis

*PTEN* expression was categorised into quartiles and the top and bottom quartiles were used for differential expression analysis, which was performed using either the limma package [41] or an orthogonal method, csSAM [42], on the complete TCGA set (*N*=489) and in the subset of low ACTA2 tumours (*N*=122). Differentially expressed genes were selected after correction for the false discovery rate for multiple testing and using a cut-off *P*<0.05. The top 50 differentially expressed genes were selected for further unsupervised hierarchical clustering and visualisation using the made4 library [43]. Unsupervised hierarchical clustering was performed using the Euclidean distance metric and Ward’s method.

### Statistical tests and survival analysis

All statistical analysis were performed using R [44]. All the R code necessary to replicate the data analysis is available in Additional file 6. Survival analysis was performed using a Cox proportional hazards model. Since some patients died just after diagnosis and were not included in the SEARCH study, left truncation was included in the analysis of this cohort, which means that we took into account both the time from diagnosis to entry into the study and the time from entry into the study to censoring or death.

### PTEN immunostaining, scanning and scoring of SEARCH and NOT samples

IHC was carried out using tissue microarrays obtained from Formalin Fixed, Paraffin-Embedded (FFPE) tissues and primary antibodies for PTEN and AR (Cell Signaling, Danvers, MA, USA; PTEN – Clone 138G6; AR – Clone D6F11). The staining protocol for PTEN clone 138G6 was previously described by FCM [8] and validated for specificity and sensitivity using *Pten* knockout mice and xenograft tissue from PTEN-positive and -negative human cancer cell lines (see Supplementary Figure 1 in [8]). Heat-induced antigen retrieval was carried out in 10 mmol ^−1^ citric acid (pH 6.0) in a pressure cooker at 120°C for 10 min. Sections were incubated with PTEN or AR antibodies overnight, diluted 1:100 in 5% goat serum, followed by incubation with anti-rabbit biotinylated secondary antibody for 1 h and peroxidase-conjugated avidin-biotin complexes (Elite ABC; Vector Laboratories, Burlingame, CA, USA). Formed immunocomplexes were visualised using diaminobenzidine (DAKO, Glostrup, Denmark, EU) and slides were counterstained with haematoxylin. Sections were rinsed in PBS between each step.

IF was carried out by adding a tyramide signal amplification step (Perkin-Elmer) after the secondary antibody, followed by incubation with Alexa Fluor 647-conjugated streptavidin (Invitrogen). Nuclei were counterstained with 4^′^,6-diamidino-2-phenylindole (DAPI) (Invitrogen).

IF and IHC samples were stored at −20°C and room temperature (RT), respectively, for at least 48 h before image analysis. For IHC, an Ariol scanning system (Leica, Wetzlar, Germany, EU) was used to obtain the digital images. For IF, images were acquired with a SP5 Leica Confocal Microscope, ×40 plan objective, and analysed by Leica software (Leica Application Suite, Advanced Fluorescence 2.2.0). Scoring was performed according to intensity (using stromal cells as internal positive controls) and percentage of stained cells by two independent observers (FCM and MJL). We subdivided tumours as negative (no staining in any tumour cell), weak positive (all tumour cells weakly stained compared to stromal cells), positive (all tumour and stromal cells equally stained) or heterogeneous (combination of positive and negative/weak staining) staining.

### Western-blot PTEN quantification in relation to *TP53* status

Western blot analysis was performed for extracts from SKOV3 ovarian cancer cell lines (ATCC; HTB-77) and derivatives obtained by Bacterial artificial chromosome (BAC) lipofectamine transfection. A BAC modification kit (Gene Bridges, Heidelberg, Germany, EU; catalogue number K002) was used to obtain the BAC clones (empty control and with R175H or R273H mutations) from the original BAC containing wild-type *TP53* (CTD-3049A20; Invitrogen, catalogue number 96012). Whole-cell extracts were collected after scraping cells in protein lysis buffer containing 50 mmoll ^−1^ Tris-HCl (pH 7.4), 150 mmoll ^−1^ NaCl, 5 mmoll ^−1^ ethylenediaminetetraacetic acid (EDTA), 50 mmoll ^−1^ NaF, 0.5% NP40, one Complete™ Mini and EDTA free tablet per 50 ml. Protein concentrations were determined using Bio-Rad protein assay kit (Hercules, CA, USA). Equal concentrations were resolved by sodium dodecyl sulphate polyacrylamide gel electrophoresis (SDS-PAGE), transferred to Immobilon-fluorescence PVDF membrane (Millipore, Bedford, MA, USA), blocked and probed with anti-human TP53 (clone DO-1, Santa Cruz, Dallas, Texas, USA; 1:2,000; overnight incubation at 4°C), anti-human PTEN (clone 138G6, Cell Signaling; 1:1,000; overnight incubation at 4°C) and anti GAPDH (Cell Signaling; 1:5,000; overnight incubation at 4°C) antibodies. IRDye800-conjugated anti-rabbit immunoglobulin G (IgG) and IRDye700-conjugated anti-mouse IgG (Li-Cor, Lincoln, NE, USA; 1:5,000 and 1:10,000 dilutions; incubated at RT for 1 h) were used as secondary antibodies. Signal intensities were analysed by using the Odyssey infrared image system (Li-Cor, Lincoln, NE, USA). Two replicates of the experiment were performed and similar results were obtained.

## Additional files

Additional file 1
**Figure S1.** Automated quantification of stroma correlates well with manual scoring. Good correlation was observed between automated stromal scoring and by eye scoring (*r*=0.593, *N*=266 images; Jonckheere–Terpstra test for trend *P*=0.001).

Additional file 2
**Supplementary Tables.** Pathological and clinical data from SEARCH, NOT and Hanrahan cohorts. NOT, Nottingham Ovarian Cancer Study; SEARCH, Study of Epidemiology and Risk Factors in Cancer Heredity.

Additional file 3
**Figure S2.** PTEN IHC staining correlates with PTEN IF staining and shows prognostic value. **(A)** Contingency table showing strong correlation between scores obtained from PTEN IF and IHC stainings (chi-squared test, *P*≪0.001). **(B)** Combined SEARCH and NOT studies (using IHC) (multivariate hazard ratio 1.5, 95% CI 1.1 to 2.0), *P*=0.006. CI, confidence interval; IF, immunofluorescence; IHC, immunohistochemistry; NOT, Nottingham Ovarian Cancer Study; SEARCH, Study of Epidemiology and Risk Factors in Cancer Heredity.

Additional file 4
**Supplementary Information.** Supplementary information includes all code for the generation of plots and statistical analyses in R.

Additional file 5
**Code for image Analysis.** Code used to estimate stromal content in TCGA histopathological images.

Additional file 6
**R code used for data analysis and to obtain all the results and figures in this publication.** R code.

## Abbreviations

AR: androgen receptor
CI: confidence interval
CNA: copy number alteration
EOC: endometrioid ovarian cancer
GAPDH: glyceraldehyde 3-phosphate dehydrogenase
GSEA: Gene Set Enrichment Analysis
H&E: hematoxylin and eosin
HGSOC: high-grade serous ovarian carcinoma
IF: immunofluorescence
IHC: immunohistochemistry
miRNA: microRNA
NOT: Nottingham Ovarian Cancer Study
PBS: phosphate-buffered saline
SEARCH: Study of Epidemiology and Risk Factors in Cancer Heredity
TCGA: The Cancer Genome Atlas
