# Supplementary figures and images for: Combined image and genomic analysis of high-grade serous ovarian cancer reveals PTEN loss as a common driver event and prognostic classifier
Source: Genome Biol. 2014 Dec 17;15(12):526. doi: 10.1186/s13059-014-0526-8 (PMC4268857; doi:10.1186/s13059-014-0526-8)

**Computed Stromal Fraction**

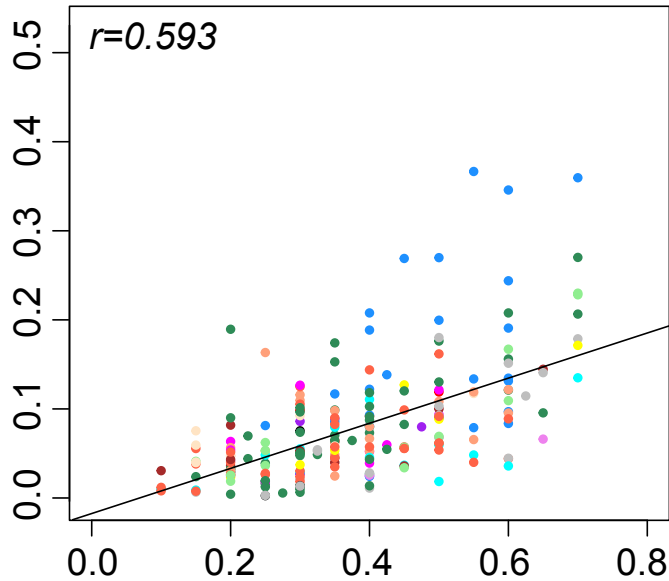

*JT P value = 0.001*

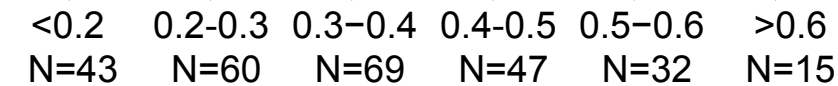

Supplement: Additional file 1 — Figure S1. Automated quantification of stroma correlates well with manual scoring. Good correlation was observed between automated stromal scoring and by eye scoring (r=0.593, N=266 images; Jonckheere–Terpstra test for trend P=0.001). [file 13059_2014_526_MOESM1_ESM.pdf]

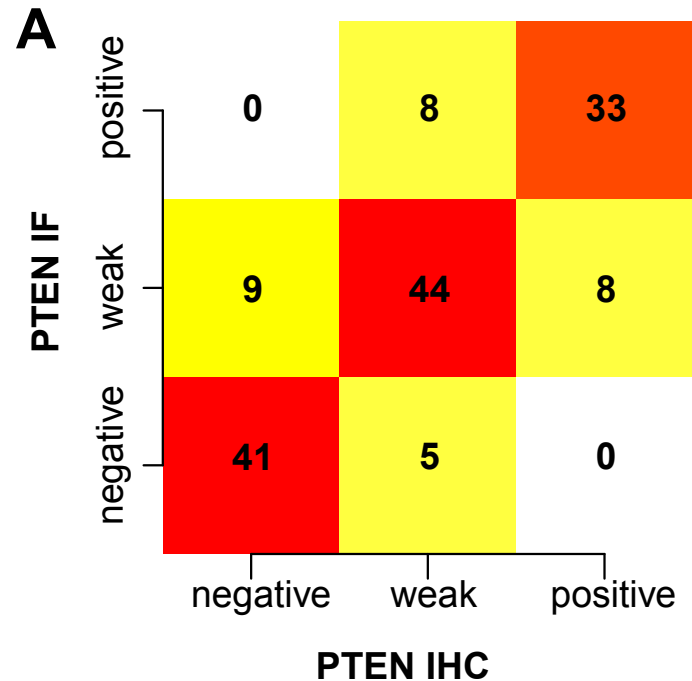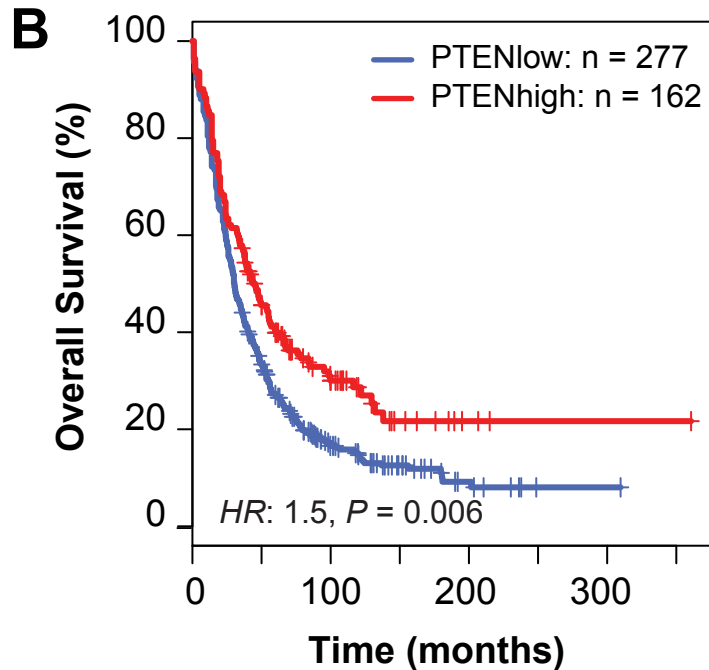

Supplement: Additional file 3 — Figure S2. PTEN IHC staining correlates with PTEN IF staining and shows prognostic value. (A) Contingency table showing strong correlation between scores obtained from PTEN IF and IHC stainings (chi-squared test, P≪0.001). (B) Combined SEARCH and NOT studies (using IHC) (multivariate hazard ratio 1.5, 95% CI 1.1 to 2.0), P=0.006. CI, confidence interval; IF, immunofluorescence; IHC, immunohistochemistry; NOT, Nottingham Ovarian Cancer Study; SEARCH, Study of Epidemiology and Risk Factors in Cancer Heredity. [file 13059_2014_526_MOESM3_ESM.pdf]

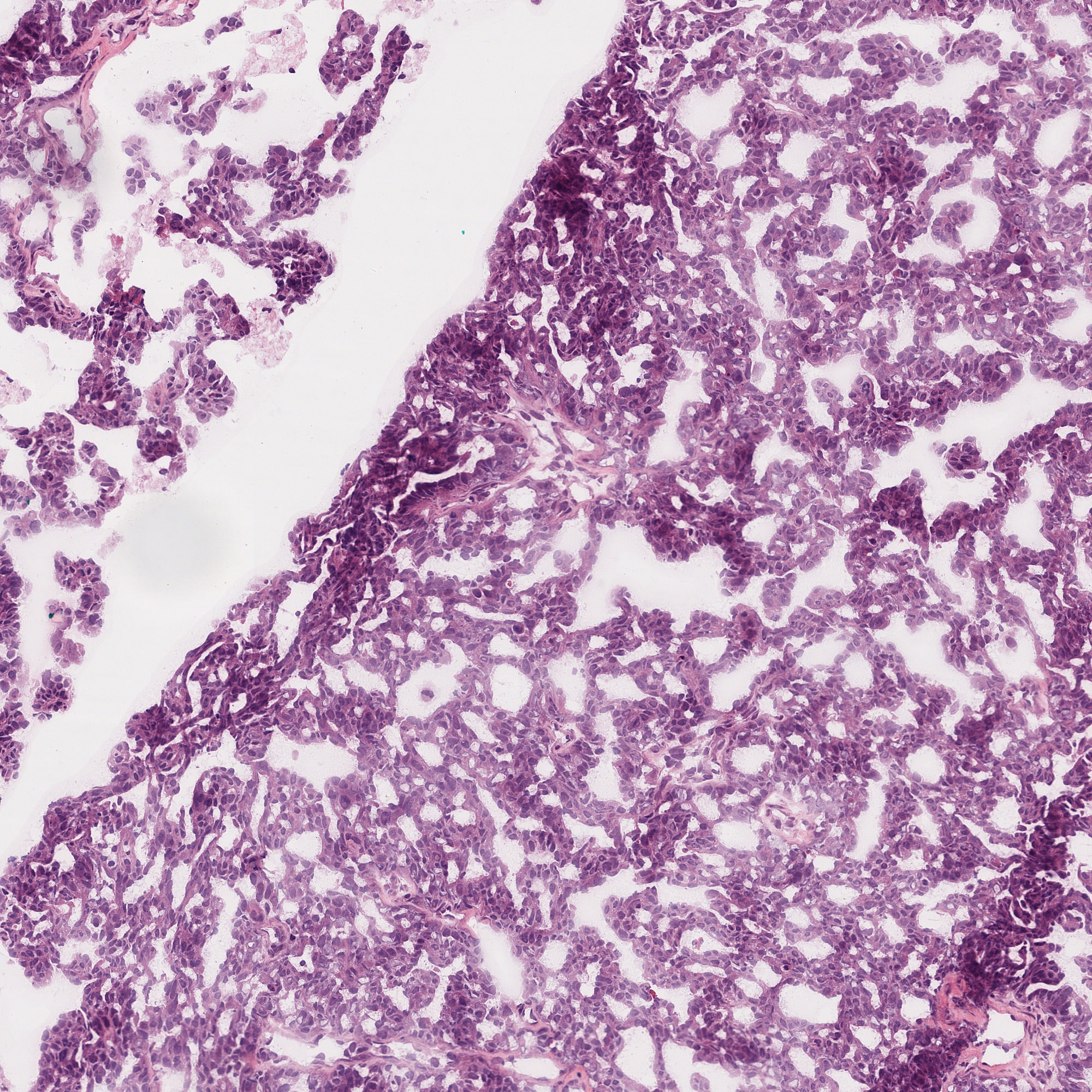

Supplement: Additional file 5 — Code for image Analysis. Code used to estimate stromal content in TCGA histopathological images. [file 13059_2014_526_MOESM5_ESM.zip › SSHE/sampleImages/10-0928-01A-BS1-Da123.jpg]

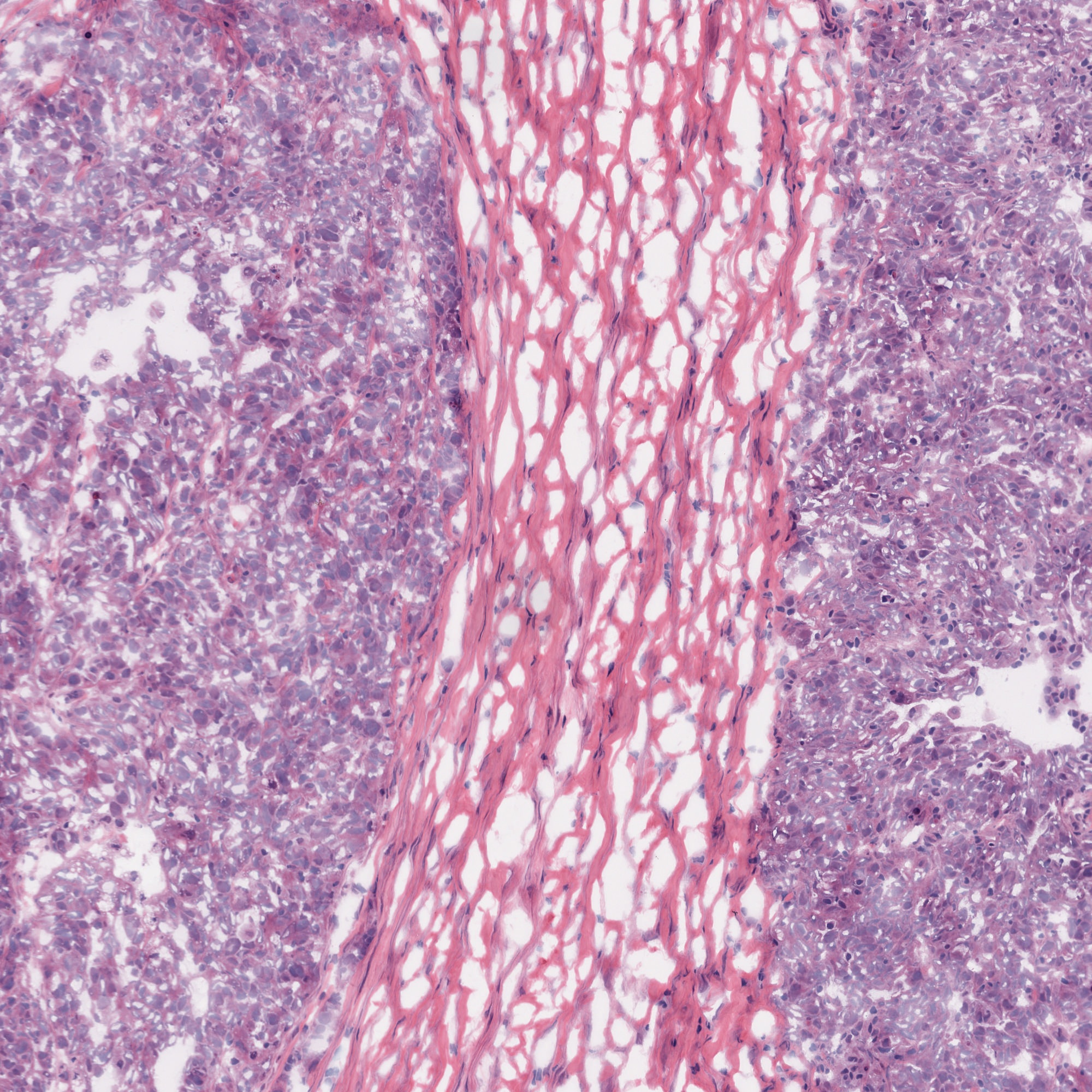

Supplement: Additional file 5 — Code for image Analysis. Code used to estimate stromal content in TCGA histopathological images. [file 13059_2014_526_MOESM5_ESM.zip › SSHE/sampleImages/61-1995-01A-BS1-Da193.jpg]

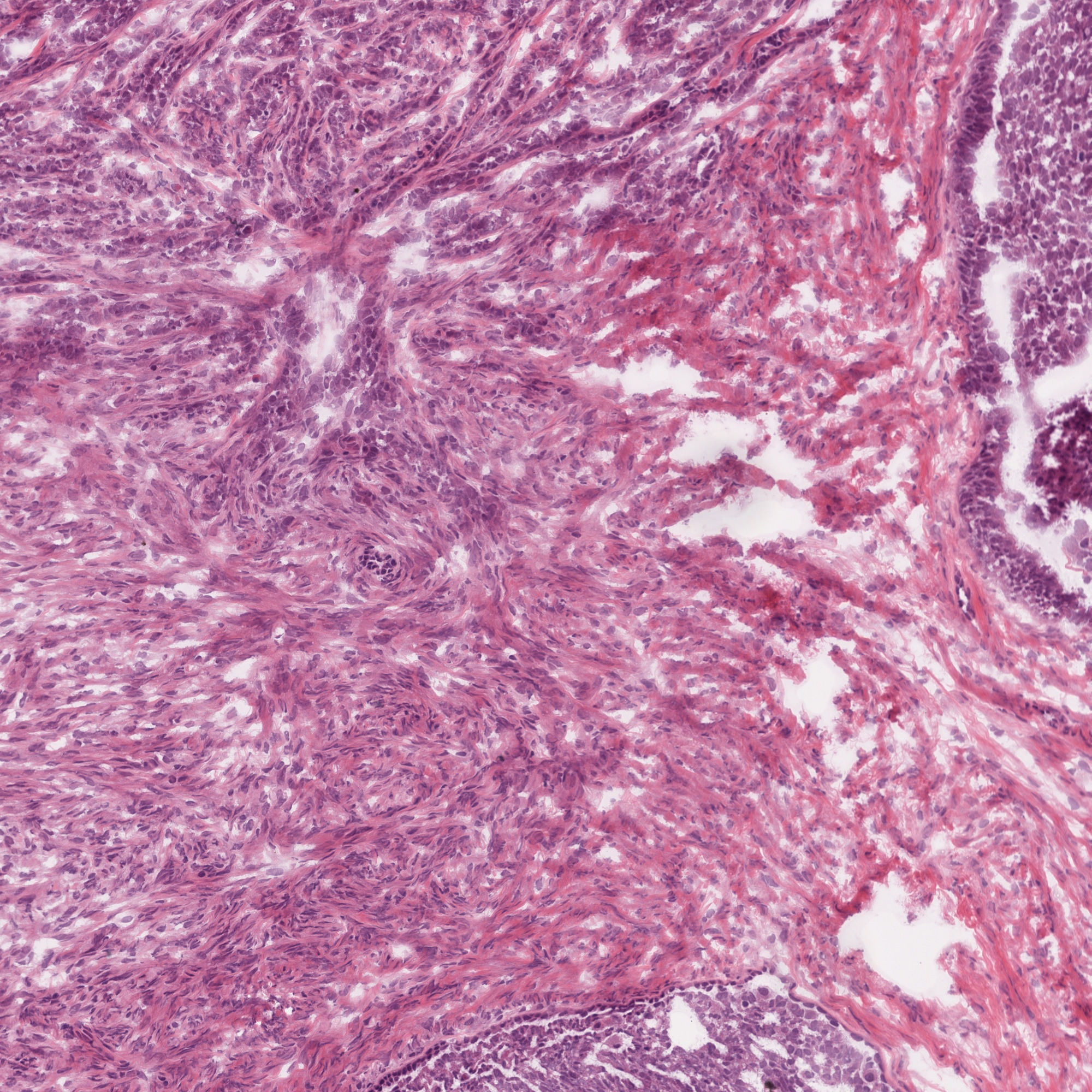

Supplement: Additional file 5 — Code for image Analysis. Code used to estimate stromal content in TCGA histopathological images. [file 13059_2014_526_MOESM5_ESM.zip › SSHE/sampleImages/61-2016-01A-BS1-Da69.jpg]

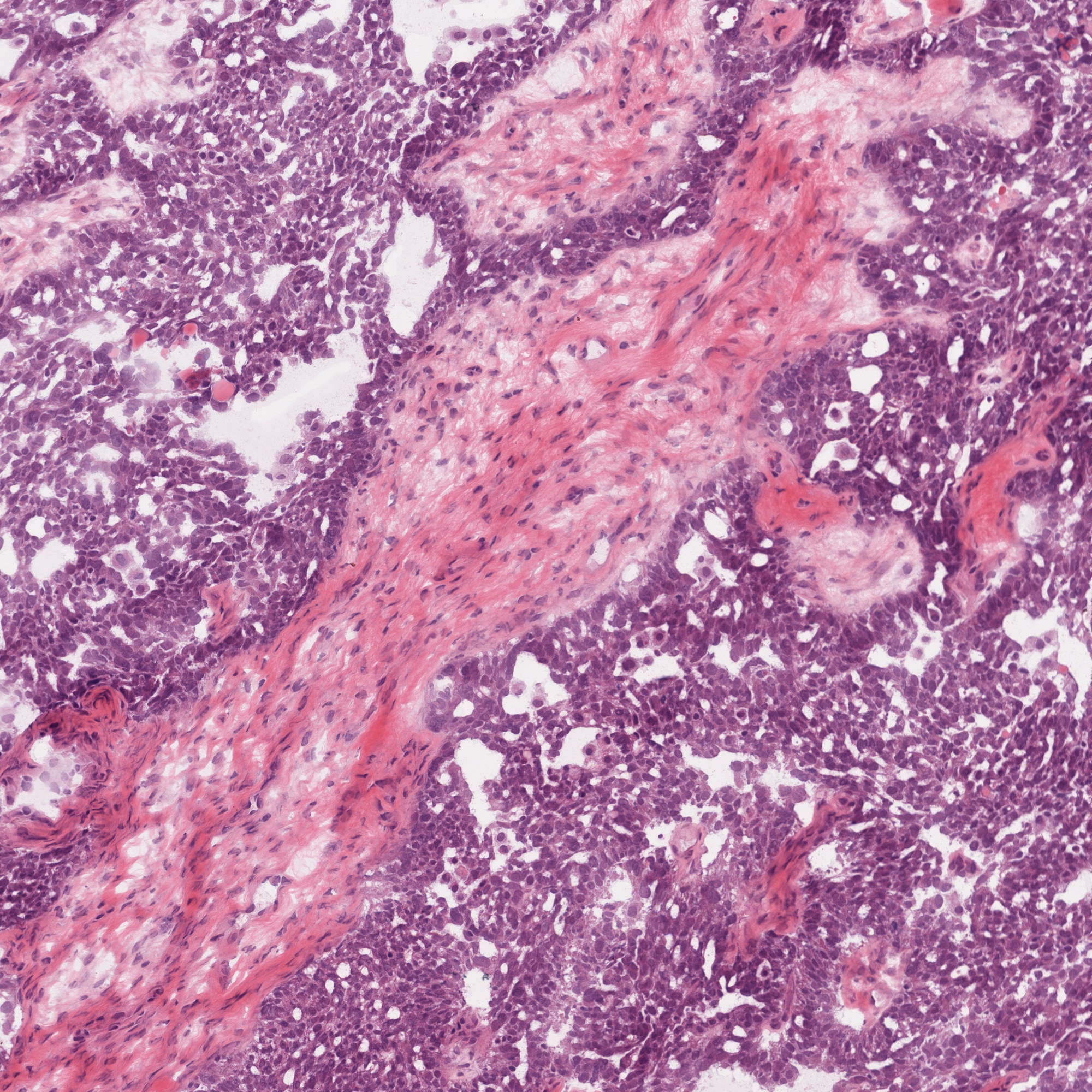

Supplement: Additional file 5 — Code for image Analysis. Code used to estimate stromal content in TCGA histopathological images. [file 13059_2014_526_MOESM5_ESM.zip › SSHE/sampleImages/61-21012_01A-TSI-Da99.jpg]
