# Supplementary material for: Combined image and genomic analysis of high-grade serous ovarian cancer reveals PTEN loss as a common driver event and prognostic classifier
Source: Genome Biol. 2014 Dec 17;15(12):526. doi: 10.1186/s13059-014-0526-8 (PMC4268857; doi:10.1186/s13059-014-0526-8)
Supplement: Additional file 4 — Supplementary Information. Supplementary information includes all code for the generation of plots and statistical analyses in R. [file 13059_2014_526_MOESM4_ESM.pdf]

Supporting Information for Martins *et al.* (2014)

June 12, 2014

Contents

1 Libraries and functions **3**

2 External Data Sources **4**

2.1 TCGA data 5
2.2 PTEN immunohistochemistry on TCGA samples 5
2.3 Gene signatures for expression subtypes and stromal scoring 6
2.4 cBioData 6
2.5 SEARCH cohort 7
2.6 Nottingham cohort 10

3 Exploring the relationship between PTEN gene expression and stromal content **13**

3.1 Manual and automatic scoring of stromal content 13
3.2 Automatic evaluation of stromal content of 302 HGSC tissue slides from the TCGA [Fig. S1A, S1B] 15
3.3 Is ACTA2 a good marker for stromal content? [Fig. 1B, 1C] 19
3.4 Distribution of PTEN vs ACTA2 mRNA expression [Fig. 1D] 24
3.5 Selecting a subgroup of TCGA samples with low ACTA2 expression levels 26
3.6 Differential expression analysis [Fig. 1E] 27
3.7 Low vs High PTEN expression is confounded by stroma [Fig. 1F] 30

4 Survival differences between PTEN-high and PTEN-low in HGSC **32**

4.1 Survival differences between PTEN-high and PTEN-low in HGSC from the SEARCH and Nottingham cohorts [Fig. 2B, 2C, S2B] 32
4.2 Multivariate Cox-hazard model 38
4.3 Correlation between immunofluorescence (IF) and immunohistochemistry (IHC) staining in samples from the SEARCH cohort [Fig. S2A] 42
4.4 BRCA germline mutation against PTEN expression in SEARCH cohort . 43

5 Exploring the relationship between PTEN loss with PTEN DNA methylation and copy number alterations 45
5.1 Relationship between PTEN methylation and expression levels in the TCGA samples [Fig. 3B] 45
5.2 Distribution of PTEN ploidy within the TCGA dataset [Fig. 3C] 47
5.3 Relationship between PTEN ploidy and expression levels in the TCGA samples [Fig. 3D] 48
5.4 PTEN gene expression within each IHC staining group [Fig. 3F and 3G] . 50
5.5 The Hanrahan 'positive' samples can be resolved into weak, het and pos categories 54
5.6 Correlation between scores for PTEN IHC staining and PTEN CNA [Fig. 3H] 56

6 Testing the prognostic value of AR and its association with PTEN expression 58
6.1 Verifying AR as a differentially expressed gene using csSAM 58
6.2 Survival Analysis using data from the TCGA cohort [Fig. 4B] 61
6.3 Validation of the survival analysis using data from the SEARCH cohort [Fig. 4C, 4D] 63

7 Differentiated and proliferative ovarian cancer subtypes are associated with high and low PTEN expression 70
7.1 Association between HGSC TCGA subtypes and expression of CD3, ACTA2, AR and PTEN in the TCGA dataset [Fig. 5A] 70
7.2 Association between PTEN expression and the diferentiated and proliferative subtypes [Fig. 5B] 75

8 Session info: R-packages and their versions used for this analysis 77

1 Libraries and functions

The R scripts provided in this vignette fully reproduces the statistical analysis of Martins et al (2014). The following R packages may be required:

- `limma` (Smyth et al., 2005)
- `made4` (Culhane et al., 2005)
- GSEA (from Broad Institute) (Barbie et al., 2009)
- `cluster` (Maechler et al., 2013)
- `survival`
- `clinfun` (Seshan, 2013)
- `heatmap.plus` (Day, 2012)
- `csSAM` (Shen-Orr et al., 2010)
- `RColorBrewer` (Neuwirth, 2011)
- `scales`
- `quantreg`

We use a local library for some low level functions. This will be loaded from `http://www.markowetzlab.org/supplements/` if not present on the local machine.

```
## loaddata file from local copy or from URL
if (file.exists("../../lib/SweaveFunctionsScript.R")) {
    source("../../lib/SweaveFunctionsScript.R")
    cat("SweaveFunctionsScript lib loaded from local copy")
} else {
    source("http://www.markowetzlab.org/supplements/Martins2014-SweaveFunctionsScript.R")
    cat("SweaveFunctionScript lib loaded from URL")
}

## Loading required package:  ade4
## Loading required package:  RColorBrewer
## Loading required package:  gplots
## KernSmooth 2.23 loaded
## Copyright M. P. Wand 1997-2009
##
## Attaching package:  'gplots'
##
## The following object is masked from 'package:stats':
```

```
##
##    lowess
##
## Loading required package:  scatterplot3d
## Loading required package:  splines
## Loading required package:  compiler
## Loading required package:  SparseM
##
## Attaching package:  'SparseM'
##
## The following object is masked from 'package:base':
##
##    backsolve
##
##
## Attaching package:  'quantreg'
##
## The following object is masked from 'package:survival':
##
##    untangle.specials
```

2 External Data Sources

The package Martins2014-supplement-data.Rdata contains the publicly available data described in the following subsections. This will be loaded from `http://www.markowetzlab.org/supplements/` if not present on the local machine.

```
## load data file from local copy or from URL
if (file.exists("../../data/Martins2014-supplementary-data.Rdata")) {
    load("../../data/Martins2014-supplementary-data.Rdata")
    cat("Martins2014-supplementary-data.Rdata loaded from local copy")
} else {
    load(url("http://www.markowetzlab.org/supplements/Martins2014-supplementary-data.Rdata
    cat("Martins2014-supplementary-data.Rdata loaded from URL")
}

## Martins2014-supplementary-data.Rdata loaded from local copy
```

2.1 TCGA data

Data from 489 high-grade serous ovarian cancers reported by The Cancer Genome Atlas Network (2012) were obtained from `https://tcga-data.nci.nih.gov/docs/publications/ov_2011/`. The `TCGA.GeneExpMat` object contains the gene expression matrix obtained from `https://tcga-data.nci.nih.gov/docs/publications/ov_2011/`:

```
dim(TCGA.GeneExpMat)

## [1]   489 11864
```

The `TCGA.ClinData` object contains the clinical annotations for all samples obtained from: `https://tcga-data.nci.nih.gov/docs/dictionary/TCGA_BCR_DataDictionary.xml`

```
dim(TCGA.ClinData)

## [1] 489  13

colnames(TCGA.ClinData)

##  [1] "BCRPATIENTBARCODE"              "AgeAtDiagnosis.yrs"
##  [3] "VITALSTATUS"                    "TUMORSTAGE"
##  [5] "TUMORGRADE"                     "TUMORRESIDUALDISEASE"
##  [7] "PRIMARYTHERAPYOUTCOMESUCCESS" "PERSONNEOPLASMCANCERSTATUS"
##  [9] "OverallSurvival.mos"            "ProgressionFreeStatus"
## [11] "ProgressionFreeSurvival.mos"  "PlatinumFreeInterval.mos"
## [13] "PlatinumStatus"
```

2.2 PTEN immunohistochemistry on TCGA samples

PTEN expression was previously assessed by immunohistochemistry (IHC) in 51 TCGA tumour samples Hanrahan et al. (2012).

```
dim(Hanrahan2012)

## [1] 100  11

colnames(Hanrahan2012)

##  [1] "OverallSurvival.mos."            "VITALSTATUS"
##  [3] "ProgressionFreeStatus"           "ProgressionFreeSurvival..mos.."
##  [5] "PlatinumFreeInterval..mos.."     "PlatinumStatus"
##  [7] "PTEN"                            "PTEN.TCGA"
##  [9] "exp"                             "PTEN.genotype"
## [11] "others"
```

2.3 Gene signatures for expression subtypes and stromal scoring

Verhaak et al. (2013) described four gene signatures for the CLOVAR classification of expression subtypes in HGSC. The gene signatures were obtained from Supplementary Table 1 (Verhaak et al., 2013). Patient subtype was assigned by computing a Single-sample GSEA (ssGSEAProjection) score for each gene signature and for each patient. (The R code for this is available at the GenePattern website `http://www.broadinstitute.org/cancer/software/genepattern/modules/docs/ssGSEAProjection/4`)

```
attach(CLOVARscores)
dim(CLOVARscores)

## [1] 489  10

colnames(CLOVARscores)

##  [1] "PatSubClass.ID"
##  [2] "PatSubClass.SUBTYPE"
##  [3] "Differentiated.ssGSEA.raw.score"
##  [4] "Immunoreactive.ssGSEA.raw.score"
##  [5] "Mesenchymal.ssGSEA.raw.score"
##  [6] "Proliferative.ssGSEA.raw.score"
##  [7] "Differentiated.ssGSEA.normalized.score"
##  [8] "Immunoreactive.ssGSEA.normalized.score"
##  [9] "Mesenchymal.ssGSEA.normalized.score"
## [10] "Proliferative.ssGSEA.normalized.score"
```

The list of genes used to score each subtype were obtained from Supplementary Table 7 (Verhaak et al., 2013) together with 141 stromal-specific genes described by Yoshihara et al. (2013).

```
attach(GeneSignatures)
names(GeneSignatures)

## [1] "ClovarSig"        "ClovarIMR"        "ClovarMES"
## [4] "ClovarPRO"        "ClovarDIF"        "StromalYoshihara"
```

2.4 cBioData

The `cbiodata` object contains PTEN DNA methylation, CNA data, AR and PTEN RNAseq expression values downloaded from `http://cbioportal.org`.

```
dim(cbiodata)

## [1] 489   4
```

```r
colnames(cbiodata)

## [1] "PTEN.methyl" "PTEN.cnv"    "AR"          "PTEN"
```

2.5 SEARCH cohort

In the SEARCH cohort we have 15 variables for 516 patient samples. The table `SEARCH` contains the clinical information and histopathologic properties for each of the 516 tumours/patients from the SEARCH cohort.

```r
dim(SEARCH)

## [1] 516  15

colnames(SEARCH)

##  [1] "Germline.BRCA.mut"        "Grade"
##  [3] "Consensus.Grade"          "Stage"
##  [5] "Age.At.Diagnosis"         "Months.To.Entry"
##  [7] "Menopause.Status.At.Diag" "Month.To.Status"
##  [9] "Vital.Status"             "HISTOLOGY"
## [11] "ER"                       "PR"
## [13] "PTEN.IHC"                 "PTEN.IF"
## [15] "AR.IHC"
```

Grade varies between 1–3, with grade 1 representing low-grade and grades 2 and 3 representing high-grade. Stage varies between 1–4. Histology is classified as CCOC (clear cell ovarian cancer), EOC (endometrial ovarian cancer), HGSC (high-grade serous ovarian cancer), LGSC (low-grade serous ovarian cancer), MOC (mucinous ovarian cancer), or mixed/other subtypes.

```r
table(SEARCH$Grade, useNA = "always")

##
##    1    2    3 <NA>
##   47  129  179  161

table(SEARCH$Stage, useNA = "always")

##
##    1    2    3    4 <NA>
##  254   54  163   29   16

table(SEARCH$HISTOLOGY, useNA = "always")
```

```
##
##    CCOC     EOC    HGSC    LGSC     MOC  Others    <NA>
##      62      97     245      35      50      27       0
```

Months to entry represent the time (in months) that lasted between diagnosis and entry in the SEARCH study. Months to status represent the time (in months) from entry into the study to the vital status, either 0 (censored) or 1 (dead).

```
summary(SEARCH$Months.To.Entry)

##    Min. 1st Qu.  Median    Mean 3rd Qu.    Max.
##     4.8    19.3    25.8    33.7    36.1   211.0

summary(SEARCH$Month.To.Status)

##    Min. 1st Qu.  Median    Mean 3rd Qu.    Max.
##    15.9    61.3    98.5   110.0   152.0   361.0

table(SEARCH$Vital.Status, useNA = "always")

##
##    0    1 <NA>
##  287  229    0
```

Estrogen and progesterone receptor staining results were described in Sieh et al. (2013) and were classified as 0 (no expression), 1 (< 50% cells stained) or 2 (> 50% cells stained). Germline BRCA mutation status was obtained from the OCAC study and was scored as either tested or not tested.

```
table(ER = SEARCH$ER, PR = SEARCH$PR, useNA = "always")

##       PR
## ER       0   1   2 <NA>
##   0    114  13   2    0
##   1     26  25  10    0
##   2     56  32  46    1
##   <NA>   6   1   1  183

table(SEARCH$Germline.BRCA.mut, useNA = "always")

##
##     BRCA1     BRCA2    Failed NotTested    Tested      <NA>
##        12        20         5        75       404         0
```

The characteristics and clinical information for the SEARCH cohort are tabled below:

		HGSC	LGSC	MOC	EOC	CCOC	Others
Number of patients		245	35	50	97	62	27
Age at diagnosis (years)		57 (8.2)	53.9 (11.8)	53.4 (12.4)	54.5 (9.4)	55.8 (7.4)	56.2 (9)
Entry time (months)		23.5 (18.9 to 32.1)	26.4 (19.6 to 33.5)	28 (18.2 to 47.6)	28.7 (21.2 to 46.4)	29.4 (19.4 to 59.6)	27.8 (23.1 to 31.6)
Last follow-up time (months)		71.1 (45.4 to 105.6)	126.5 (85.7 to 149.1)	123 (83.1 to 170.3)	140.9 (86.5 to 171.2)	144.8 (101.6 to 208.2)	97.8 (73.7 to 139.7)
Vital Status	Data available	245 (100%)	35 (100%)	50 (100%)	97 (100%)	62 (100%)	27 (100%)
	Alive	89 (36%)	27 (77%)	39 (78%)	69 (71%)	47 (76%)	16 (59%)
	Died	156 (64%)	8 (23%)	11 (22%)	28 (29%)	15 (24%)	11 (41%)
Stage	Data available	238 (97%)	33 (94%)	47 (94%)	96 (99%)	62 (100%)	24 (89%)
	I	46 (19%)	27 (82%)	42 (89%)	76 (79%)	50 (81%)	13 (54%)
	II	27 (11%)	2 (6%)	1 (2%)	13 (14%)	6 (10%)	5 (21%)
	III	139 (58%)	4 (12%)	4 (9%)	6 (6%)	5 (8%)	5 (21%)
	IV	26 (11%)	0 (0%)	0 (0%)	1 (1%)	1 (2%)	1 (4%)
Grade	Data available	201 (82%)	13 (37%)	26 (52%)	78 (80%)	26 (42%)	11 (41%)
	I	1 (0%)	11 (85%)	10 (38%)	22 (28%)	2 (8%)	1 (9%)
	II	56 (28%)	1 (8%)	14 (54%)	40 (51%)	11 (42%)	7 (64%)
	III	144 (72%)	1 (8%)	2 (8%)	16 (21%)	13 (50%)	3 (27%)
PTEN status (IF)	Data available	214 (87%)	29 (83%)	41 (82%)	89 (92%)	59 (95%)	26 (96%)
	Negative	49 (23%)	2 (7%)	12 (29%)	39 (44%)	34 (58%)	8 (31%)
	Weak Negative	68 (32%)	6 (21%)	11 (27%)	27 (30%)	18 (31%)	8 (31%)
	Heterogeneous	48 (22%)	7 (24%)	2 (5%)	3 (3%)	1 (2%)	3 (12%)
	Positive	49 (23%)	14 (48%)	16 (39%)	20 (22%)	6 (10%)	7 (27%)
PTEN status (IHC)	Data available	211 (86%)	28 (80%)	44 (88%)	90 (93%)	56 (90%)	26 (96%)
	Negative	53 (25%)	3 (11%)	9 (20%)	35 (39%)	22 (39%)	4 (15%)
	Weak Negative	73 (35%)	8 (29%)	5 (11%)	28 (31%)	18 (32%)	5 (19%)
	Heterogeneous	34 (16%)	3 (11%)	6 (14%)	6 (7%)	3 (5%)	3 (12%)
	Positive	51 (24%)	14 (50%)	24 (55%)	21 (23%)	13 (23%)	14 (54%)

Data are mean (SD), median (OQR), n(%), or n. HGSC: High-Grade Serous ; LGSC: Low-Grade Serous; MOC: Mucinous; EOC: Endometrial; CCOC: Clear Cell Ovarian Cancer

2.6 Nottingham cohort

The table `NOT` shows the clinical data and histopathologic properties associated with the tumours/patients from the Nottingham cohort. In the NOT cohort we have 10 variables for 507 patients.

```
dim(NOT)

## [1] 507  10

colnames(NOT)

##  [1] "NewTMAno"         "Age.At.Diagnosis" "Grade"
##  [4] "Stage"            "month.recurfree"  "survival.months"
##  [7] "Vital.Status"     "HISTOLOGY"        "PTEN.IHC"
## [10] "CA125"
```

Grade varies between 1 and 3. Grade 1 represents low-grade and grade 3 corresponds to high-grade. Stage varies between 1 and 4. Histology is classified as CCOC (clear cell ovarian cancer), EOC (endometrial ovarian cancer), HGSC (high-grade serous ovarian cancer), LGSC (low-grade serous ovarian cancer), MOC (mucinous ovarian cancer), or mixed/other subtypes.

```
table(NOT$Grade, useNA = "always")

##
##    1    2    3 <NA>
##   51   37  277  142

table(NOT$Stage, useNA = "always")

##
##    1    2    3    4 <NA>
##  179   57  222   38   11

table(NOT$HISTOLOGY, useNA = "always")

##
##   CCOC    EOC   HGSC   LGSC Others    MOC   <NA>
##     50     62    276     40     24     54      1
```

Survival months represent the time (in months) from the diagnosis to the vital status, either 0 (censored) or 1 (dead).

```r
summary(NOT$survival.months)

##    Min. 1st Qu.  Median    Mean 3rd Qu.    Max.    NA's
##     1.0    20.0    44.0    58.2    84.0   223.0      13

table(NOT$Vital.Status, useNA = "always")

##
##    0    1 <NA>
##  191  304   12
```

Together the SEARCH and NOT cohort have 1023 patients.

```r
nrow(SEARCH) + nrow(NOT)

## [1] 1023
```

The following table summarises the characteristics and clinical information in the NOT cohort:

		HGSC	LGSC	MOC	EOC	CCOC	Others
Number of patients		276	40	54	62	50	24
Age at diagnosis (years)		62.6 (10.7)	54.4 (16)	56.6 (17.8)	60.8 (11.8)	59.4 (12.2)	62 (12.2)
Overall Survival (months)		34 (17 to 60)	80 (50 to 107.2)	63 (16 to 108)	90 (59 to 120)	52.5 (30.5 to 108.8)	44 (2 to 88.5)
Vital Status	Data available	272 (99%)	38 (95%)	53 (98%)	61 (98%)	48 (96%)	23 (96%)
	Alive	69 (25%)	30 (79%)	26 (49%)	36 (59%)	19 (40%)	11 (48%)
	Died	203 (75%)	8 (21%)	27 (51%)	25 (41%)	29 (60%)	12 (52%)
Stage	Data available	270 (98%)	40 (100%)	52 (96%)	62 (100%)	48 (96%)	24 (100%)
	I	41 (15%)	24 (60%)	42 (81%)	36 (58%)	28 (58%)	8 (33%)
	II	31 (11%)	3 (8%)	1 (2%)	13 (21%)	8 (17%)	1 (4%)
	III	168 (62%)	12 (30%)	7 (13%)	11 (18%)	12 (25%)	12 (50%)
	IV	30 (11%)	1 (2%)	2 (4%)	2 (3%)	0 (0%)	3 (12%)
Grade	Data available	197 (71%)	21 (52%)	39 (72%)	60 (97%)	38 (76%)	10 (42%)
	I	1 (1%)	20 (95%)	18 (46%)	10 (17%)	0 (0%)	2 (20%)
	II	4 (2%)	0 (0%)	15 (38%)	17 (28%)	0 (0%)	1 (10%)
	III	192 (97%)	1 (5%)	6 (15%)	33 (55%)	38 (100%)	7 (70%)
PTEN status (IHC)	Data available	233 (84%)	34 (85%)	40 (74%)	55 (89%)	43 (86%)	23 (96%)
	Negative	27 (12%)	1 (3%)	4 (10%)	18 (33%)	14 (33%)	5 (22%)
	Weak Negative	65 (28%)	5 (15%)	17 (42%)	13 (24%)	17 (40%)	6 (26%)
	Heterogeneous	29 (12%)	0 (0%)	3 (8%)	0 (0%)	1 (2%)	5 (22%)
	Positive	112 (48%)	28 (82%)	16 (40%)	24 (44%)	11 (26%)	7 (30%)

Data are mean (SD), median (OQR), n(%), or n. HGSC: High-Grade Serous; LGSC: Low-Grade Serous; MOC: Mucinous; EOC: Endometrial; CCOC: Clear Cell Ovarian Cancer

3 Exploring the relationship between PTEN gene expression and stromal content

In this section we aimed to investigate the relationship between PTEN expression and tumour stromal content. We performed the following analysis:

1. We scored the TCGA tumour images according to their tumor content in a manual and automated fashion (Figure S1A, S1B)

2. We compared our manual and automated scores with stromal scores obtained from independent sources and with ACTA2 gene expression (Figure 1B,C)

3. We confirmed that there was a good correlation between the stromal content and PTEN expression (Figure 1D)

4. Finally, we select a subgroup of TCGA samples with low ACTA2 expression, and thus low stromal contamination (Figure 1E,F)

3.1 Manual and automatic scoring of stromal content

We developed an automated system in MATLAB to analyse the H&E-stained images from TCGA and quantify the stromal content of each tumour sample (code and documentation is provided in the Supplementary Materials). Images were segmented by first applying an entropy filter to remove background from an image, followed by colour deconvolution according to Ruifroks' method (Ruifrok and Johnston, 2001). The haematoxylin channel was subtracted from the eosin channel, leaving a raw stromal signal. Otsu's thresholding and smoothing was then performed to estimate a stromal fraction (Otsu, 1979). Image data from the TCGA includes 1–3 sections from each case, designated by the prefix BS1, TS1 and BS2. Results for the automated scoring of all sections were imported into `StrFrac` in the `TCGA.BioSpecData` matrix.

```
dim(TCGA.BioSpecData)

## [1] 489  13

colnames(TCGA.BioSpecData)[1:13]

##  [1] "ID"            "NoSubImages1" "StrFrac1"     "PathScore1"
##  [5] "ImQuality1"    "NoSubImages2" "StrFrac2"     "PathScore2"
##  [9] "ImQuality2"    "NoSubImages3" "StrFrac3"     "PathScore3"
## [13] "ImQuality3"
```

Images were then scored by eye for overall quality using a binary pass:fail variable `ImQuality` based on frequently observed artefacts (including discoloration, folding over of the mounted section and completeness of the section). Manual scoring was also performed

for the percentage of stromal cells (`PathScore`). The average stromal content for each sample was computed after excluding poor quality images

```
attach(TCGA.BioSpecData)
GoodQualAverage=rowMeans(cbind(StrFrac1*ImQuality1,
                               StrFrac2*ImQuality2,
                               StrFrac3*ImQuality3), na.rm=T)
summary(GoodQualAverage)

##    Min. 1st Qu.  Median    Mean 3rd Qu.    Max.    NA's
##    0.01    0.07    0.11    0.13    0.18    0.54     273

PathologistAverage=rowMeans(cbind(PathScore1*ImQuality1,
                                  PathScore2*ImQuality2,
                                  PathScore3*ImQuality3), na.rm=T)
summary(PathologistAverage)

##    Min. 1st Qu.  Median    Mean 3rd Qu.    Max.    NA's
##     7.5    30.0    37.5    38.6    50.0    90.0     295

detach(TCGA.BioSpecData)
```

The number of patient samples used in the study were as follows:

```
length(which(!is.na(GoodQualAverage)))

## [1] 216

length(which(!is.na(PathologistAverage)))

## [1] 194
```

Filtering the data to exclude poor quality slides was performed.

```
AutoScored=!is.na(TCGA.BioSpecData[ ,grep("StrFrac", colnames(TCGA.BioSpecData))])
ImQuality=!is.na(TCGA.BioSpecData[, grep("ImQuality", colnames(TCGA.BioSpecData))])
PathScore=!is.na(TCGA.BioSpecData[ ,grep("PathScore", colnames(TCGA.BioSpecData))])
# number of scored, good quality slides
table(AutoScored, ImQuality)

##            ImQuality
## AutoScored FALSE TRUE
##      FALSE   189  842
##      TRUE    134  302
```

```r
table(PathScore, ImQuality)

##           ImQuality
## PathScore FALSE TRUE
##     FALSE   277  878
##     TRUE     46  266
```

A total of 436 images were automatically scored, from which 134 images were discarded owing to poor quality, leaving 302 tissue slides from 216 patients. Scoring by eye was performed on 312 slides, of which 46 were discarded owing to poor quality, leaving 266 slides from 194 patients.

3.2 Automatic evaluation of stromal content of 302 HGSC tissue slides from the TCGA [Fig. S1A, S1B]

As described before, 302 H&E slides were scored using the automated system and 266 slides were scored by eye for stromal content.

```r
## vectorise the scores
attach(TCGA.BioSpecData)
AutoScores <- c(StrFrac1,StrFrac2,StrFrac3)
PathScores <- c(PathScore1,PathScore2,PathScore3)/100
ImQual     <- c(ImQuality1,ImQuality2,ImQuality3)

## Overview
table(ImQual,useNA="ifany")

## ImQual
##    1 <NA>
## 1144  323

par(mfrow=c(1,2),las=1)
hist(AutoScores,20,xlim=c(0,1),main="Automatic scoring",col="grey")
hist(PathScores,20,xlim=c(0,1),main="Manual scoring",col="grey")
```

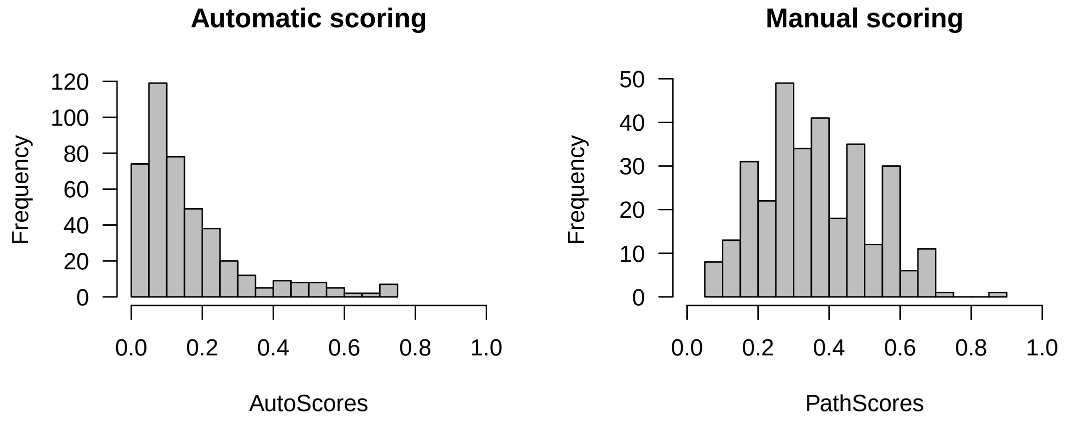

To validate the automated scoring, we compared our results (Computed Stromal Fraction) to those obtained by manual scoring.

```
#determine the batch numbers used

NIdx <- factor(substr(ID,6, 7))

#Correlation plot
par(mar=c(4.1, 4.1, 3.1, 7.1), xpd=FALSE)
plot(PathScores*ImQual, AutoScores*ImQual,
     xlab="Manual Scoring", ylab="Computed Stromal Fraction",
     col=NIdx, pch=20, main="Figure S1A: Raw Correlation", cex.main=.8,
     xlim=c(0, 0.8), ylim=c(0, 0.8))
par(xpd=TRUE)
legend(0.85, 0.82,
       legend=as.character(unique(NIdx)),
       col=1:length(levels(NIdx)), lwd=1, cex=0.75,
       title="TCGA Batch Number")
```

Figure S1A: Raw Correlation

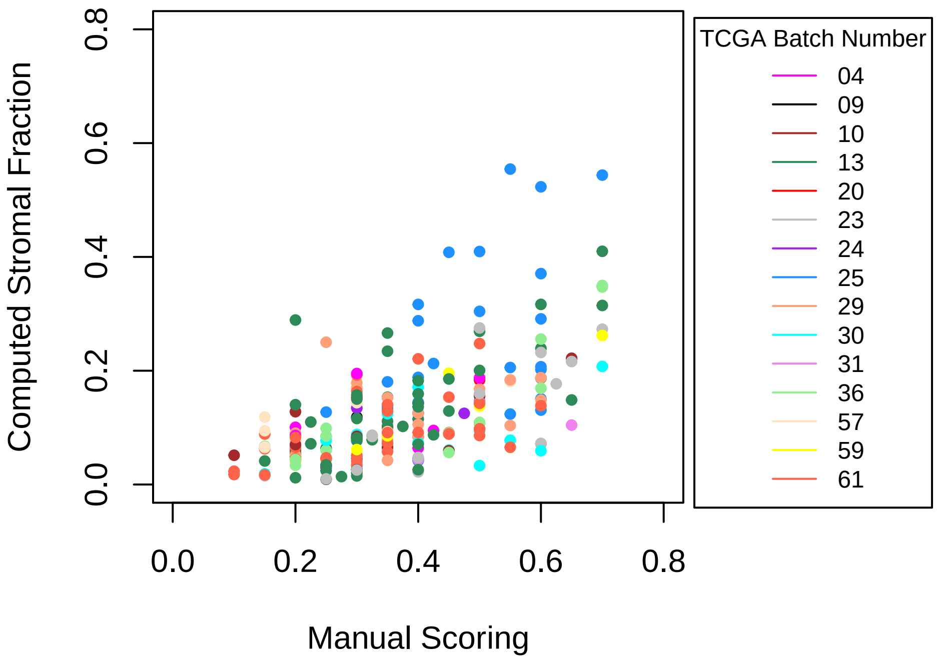


```
#Correlation Testing
cor.test(AutoScores*ImQual, PathScores*ImQual, use="complete")

##
##  Pearson's product-moment correlation
##
## data:  AutoScores * ImQual and PathScores * ImQual
## t = 11.1, df = 227, p-value < 2.2e-16
## alternative hypothesis: true correlation is not equal to 0
## 95 percent confidence interval:
##  0.5019 0.6710
## sample estimates:
##   cor
## 0.593
```

The correlation coefficient between manual and automated stromal scoring was 0.59 and no obvious batch effects were seen within the TCGA data.

As manual scoring appeared highly categorized, we split the scores into 6 different groups and tested for trend using Jonckheere's method. An increasing trend between automated and manual scoring was observed

```r
# separate manual scores into discrete groups
PathGrps <- c("<20", "20-30", "30-40", "40-50","50-60", "60+")
PatIdx <- cut(PathScores*ImQual,
              breaks=c(0, 0.2, 0.3, 0.4, 0.5, 0.60, 1.00),
              labels=PathGrps)

 boxplot(AutoScores~PatIdx,
        xaxt='n',
        ylab="Computed Stromal Fraction",
        xlab="Manual Score",
        main="Figure S1B: Distribution of computed stromal fractions
        with discrete manual scores", cex.main=.8)
 axis(1, at=seq(from=1, to=6, by=1), PathGrps)
```

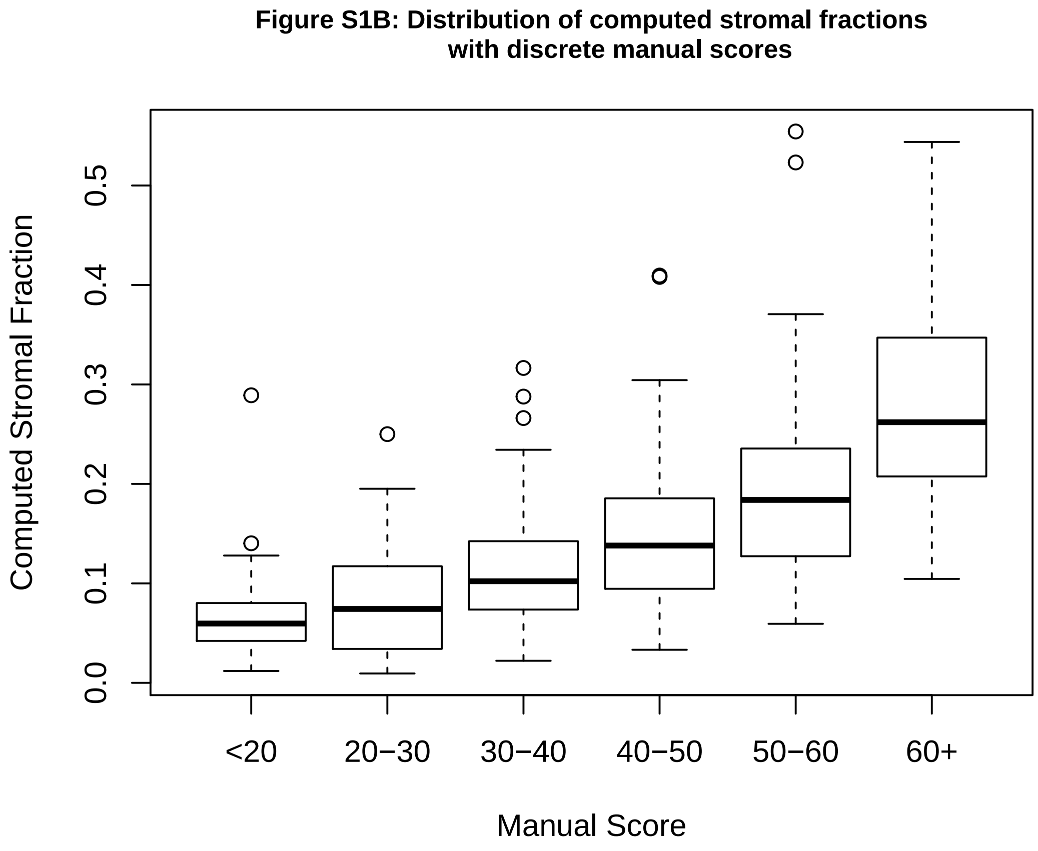


```r
jonckheere.test(AutoScores*ImQual,
                as.numeric(PatIdx),
                alternative="increasing",
```

```
                nperm=1000)

##
##  Jonckheere-Terpstra test
##
## data:
## JT = 313420, p-value = 0.001
## alternative hypothesis: increasing
```

3.3 Is ACTA2 a good marker for stromal content? [Fig. 1B, 1C]

We used the stromal signature described in Yoshihara et al. (2013) to score the stromal content of the images. We compared the automated stromal quantification with the expression of the genes in the Yoshihara signature and with ACTA2 expression.

```
# Run the correlation testing
YSig=c(StromalYoshihara, "ACTA2")
YSigCorr <- sapply(YSig, function(x) cor.test(TCGA.GeneExpMat[ ,x],
                         GoodQualAverage, use="complete")[3:4])
YSigCorr=list(pval=as.numeric(YSigCorr[1, ]),
              cor=as.numeric(YSigCorr[ 2, ]))
names(YSigCorr$pval)=YSig
names(YSigCorr$cor)=YSig
hist(YSigCorr$cor,xlim=c(-0.3,0.5),
     main="Gene expression markers for stromal content", cex.main=.8,
     xlab="Correlation gene exp vs stromal content")
```

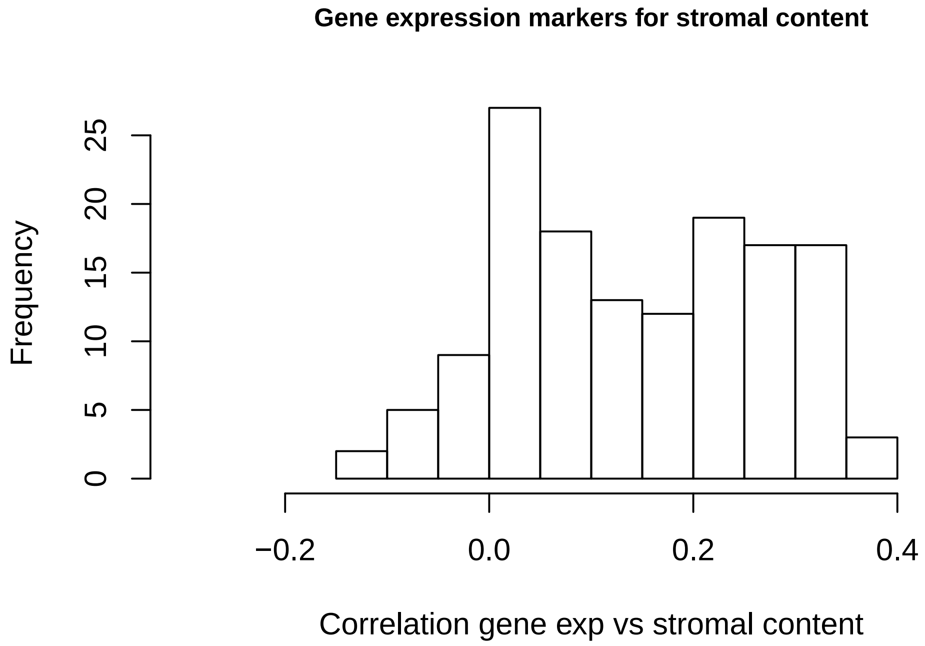


```
# Plot the correlation coefficients and/or p values
par(mfrow=c(1,2))
# Plot the pvalue ranking
ord <- order(YSigCorr$pval)
plot(1:length(YSigCorr$pval), log10(YSigCorr$pval[ord]),
     xlab="Genes [ranked by p-value]", ylab="Log10 p value",
         col=ifelse(YSig[ord]=="ACTA2","red","blue"),
                 pch=ifelse(YSig[ord]=="ACTA2",19,1), las=1,
                 xaxt='n',main="Rank of ACTA2 p-value
     with stromal content in images", cex.main=.8)
abline(h=0.3, lty=2)
text(which(YSig[ord]=="ACTA2")+24,log10(YSigCorr$pval["ACTA2"])+0.02,"ACTA2")
# Plot the correlation ranking
ord <- order(YSigCorr$cor)
plot(1:length(YSigCorr$cor), YSigCorr$cor[ord],
     xlab="Genes [ranked by correlation]",
     ylab="Correlation Coefficient",
                 col=ifelse(YSig[ord]=="ACTA2","red","blue"),
                 pch=ifelse(YSig[ord]=="ACTA2",19,1),
     las=1,
                 xaxt='n',
     main="Rank of ACTA2 correlation
```

```
     with stromalcontent in images", cex.main=.8)
abline(h=0.3, lty=2)
text(which(YSig[ord]=="ACTA2")-24,YSigCorr$cor["ACTA2"]+0.02,"ACTA2")
```

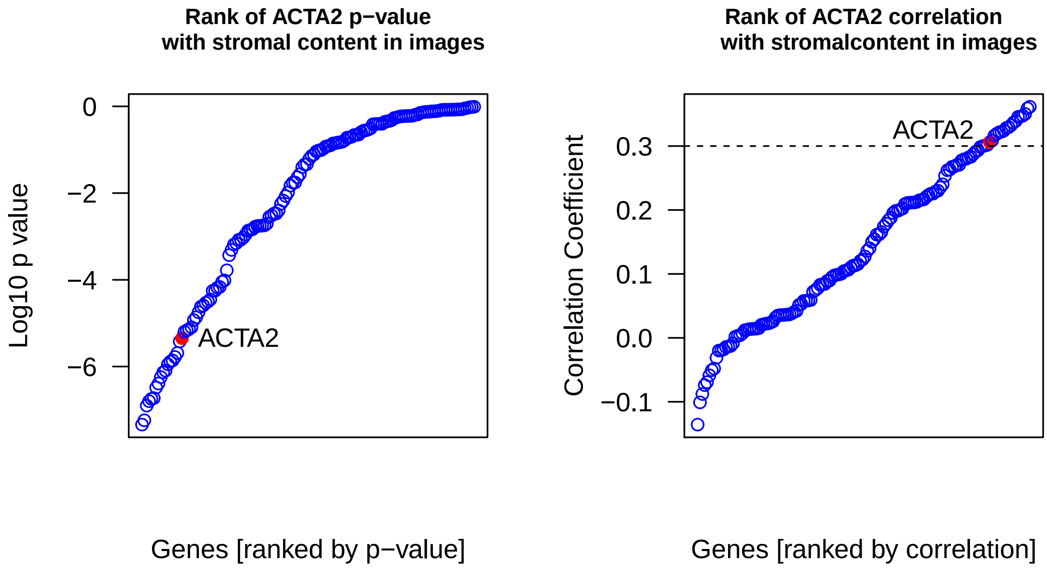


```
# Correlation with ACTA2 and genes
YSigCorr$cor["ACTA2"]

##  ACTA2
## 0.3063
```

ACTA2 is one of the top genes correlated with stromal scoring (ranked 17). The following heatmap compares ACTA2 with the other top ranked stromal genes in the tumour signatures. We correct for p-values using the BH method and select a p-value cutoff of 0.01 in the following heat-map.

```
# Heatmap of the top scoring genes
YSigCorr$padj=p.adjust(YSigCorr$pval, method="BH", n=ncol(TCGA.GeneExpMat))
HighYSig=YSig[which(YSigCorr$padj<0.01)]
heatmap(t(TCGA.GeneExpMat[ , HighYSig]), col=brewer.pal(9, "RdBu"),
        margins=c(0,5))
```

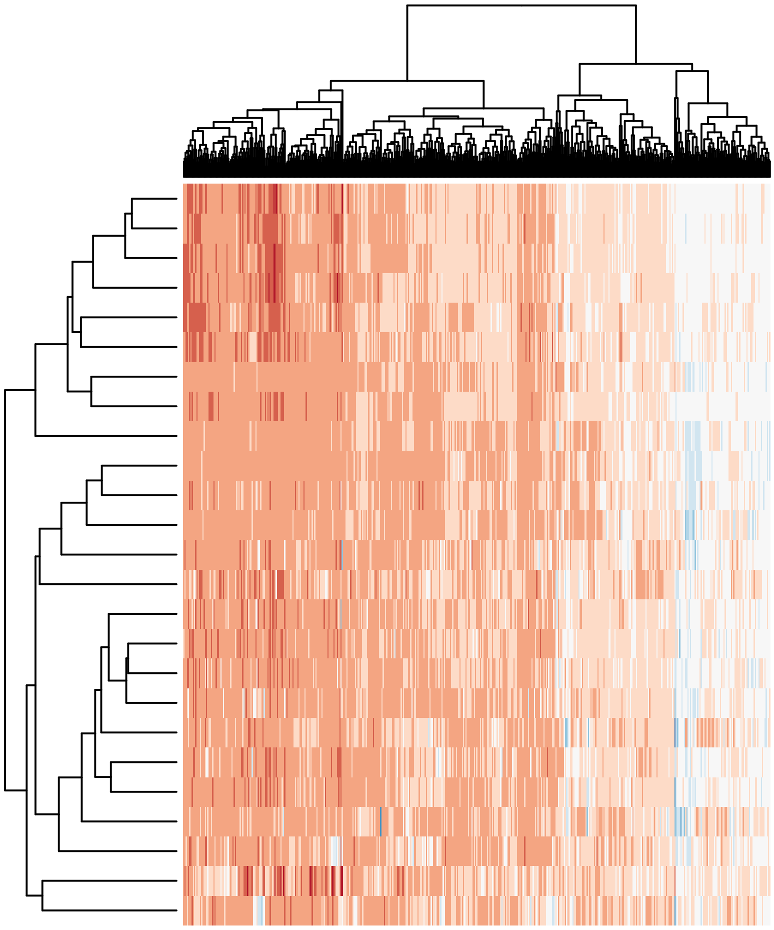

We next explored the correlation between the average expression of these stromal markers with the automated system (averaged over up to 3 slides per patient).

```
# Compute the average expression level
YSigGE=rowMeans(TCGA.GeneExpMat[ , HighYSig])

## Plot expression with observed stromal content
par(mar=c(4.1, 4.1, 3.1, 7.1), xpd=FALSE)
plot(YSigGE, GoodQualAverage, pch=20, col=NIdx,
     xlab="Stromal Signature Score",
     ylab="Computed Stromal Fraction",
     main="Fig 1B: Correlation between stromal
     signature and image analysis", cex.main=.8)

## regression line
a1=lm(GoodQualAverage~YSigGE)
abline(a1, col="red", lwd=3)
```

```r
## add legend
par(xpd=TRUE)
legend(3, 0.6,  legend=as.character(unique(NIdx)),
       col=1:length(unique(NIdx)), lwd=1,
       cex=0.75,title="TCGA Batch Number")
```

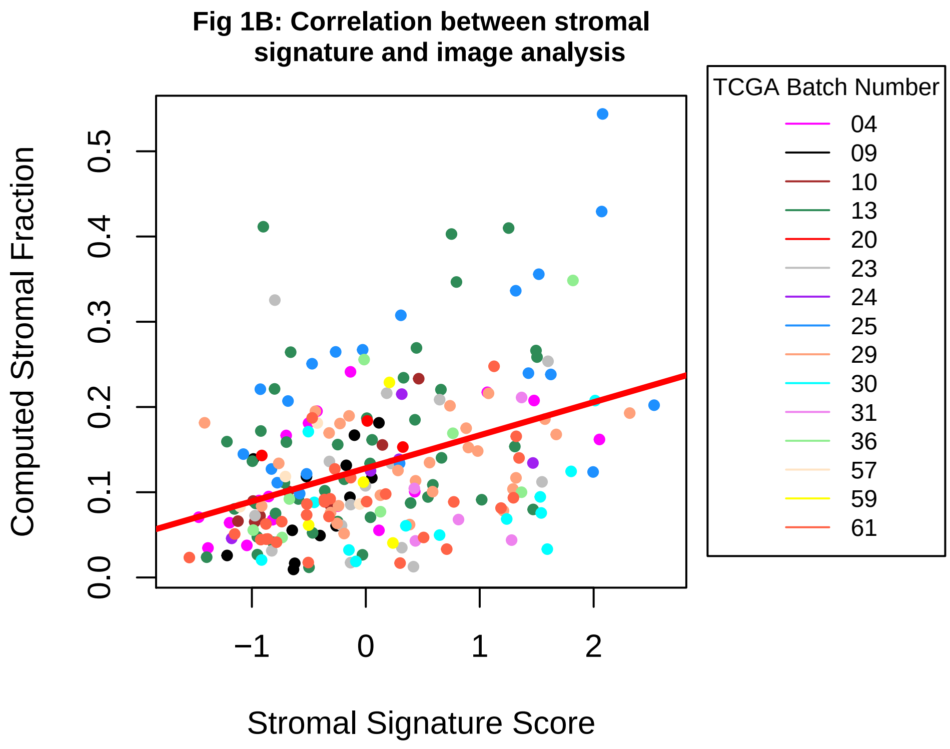


```r
par(xpd=F)

# Correlation test
cor.test(GoodQualAverage,YSigGE, use="complete")

##
##  Pearson's product-moment correlation
##
## data:  GoodQualAverage and YSigGE
## t = 6.336, df = 214, p-value = 1.374e-09
## alternative hypothesis: true correlation is not equal to 0
## 95 percent confidence interval:
##  0.2787 0.5042
## sample estimates:
##    cor
## 0.3974
```

3.4 Distribution of PTEN vs ACTA2 mRNA expression [Fig. 1D]

```
breakVals=quantile(TCGA.GeneExpMat[ ,"ACTA2"], c(0, 0.25, 0.5, 0.75,1))
data_new <- cut(TCGA.GeneExpMat[ ,"ACTA2"],breaks=breakVals, label=c(1,2,3,4))
par(mfrow=c(1,2))
boxplot(TCGA.GeneExpMat[ ,"PTEN"]~data_new, notch = T,
        ylab="ACTA2 gene expression (in quartiles)",
        names=c("Q1","Q2","Q3","Q4"),
        horizontal=T,
        xaxt='n', las=2, col=alpha(c("dodgerblue", "powderblue",
                                     "rosybrown1", "firebrick1"), 0.5))
# Define the x (PTEN expression) and y (stroma) variables to be plotted
xVal=TCGA.GeneExpMat[ ,"PTEN"]
yVal = TCGA.GeneExpMat[ ,"ACTA2"]
RangeX = range(xVal)*c(1.05, 1.1)
# plot the Stromal content against PTEN
colors <- densCols(xVal, yVal, colramp = colorRampPalette(c("gray65", "black")))
plot(NA,xlim=range(xVal),ylim=range(yVal),
     xlab="PTEN expression", ylab="Stromal Content", bg="gray")
rect(RangeX[1],breakVals[1]*1.05,RangeX[2],
     breakVals[2],col=alpha("dodgerblue", 0.25),lty=0)
rect(RangeX[1],breakVals[2],RangeX[2],
     breakVals[3],col=alpha("powderblue",0.25), lty=0)
rect(RangeX[1],breakVals[3],RangeX[2],
     breakVals[4],col=alpha("pink", 0.25),lty=0)
rect(RangeX[1],breakVals[4],RangeX[2],
     breakVals[5]*1.05,col=alpha("firebrick1", 0.25),lty=0)
abline(h=breakVals[2:4],col="grey")
points(xVal, yVal, col = colors, pch =19)

title(main="Figure 1D: PTEN expression per ACTA2 quartile",
      cex.main=.9, outer=TRUE, line=-1)

# regression line
a1=lm(yVal~xVal)
abline(a1, lwd=3, col="red")
```

Figure 1D: PTEN expression per ACTA2 quartile

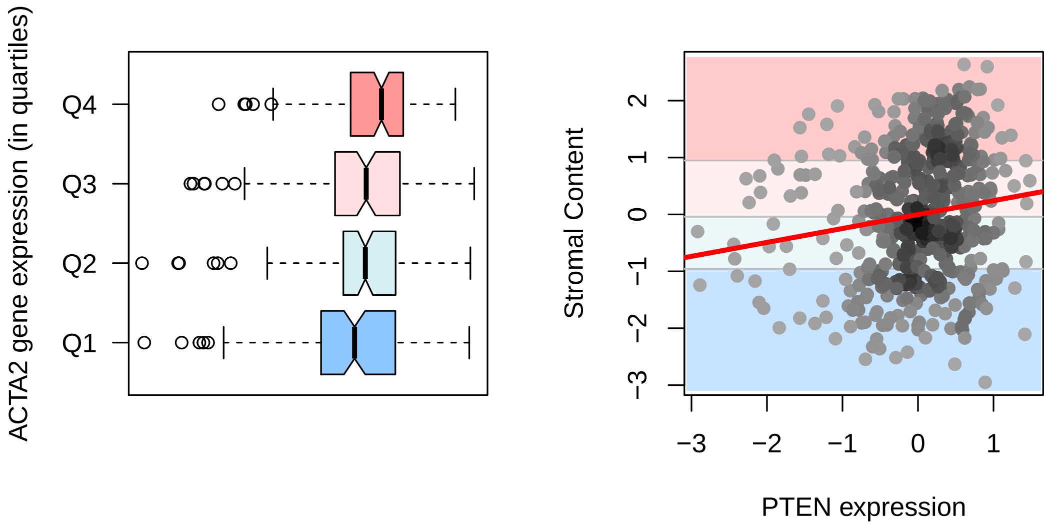


```
# test for correlation
cor.test( yVal, xVal,use="complete")

##
##  Pearson's product-moment correlation
##
## data:  yVal and xVal
## t = 3.389, df = 487, p-value = 0.0007574
## alternative hypothesis: true correlation is not equal to 0
## 95 percent confidence interval:
##  0.0640 0.2373
## sample estimates:
##    cor
## 0.1518
```

```
# Can also do a test for trend (increasing PTEN values)
jonckheere.test(TCGA.GeneExpMat[ ,"PTEN"],
                as.numeric(data_new),
                alternative="increasing",
                nperm=1000)

##
##  Jonckheere-Terpstra test
##
```

```
## data:
## JT = 50700, p-value = 0.003
## alternative hypothesis: increasing
```

Performing a regression, we see that the correlation coefficient is 0.1518 and performing a test for trend using the quartiles gives a P value of 0.003.

3.5 Selecting a subgroup of TCGA samples with low ACTA2 expression levels

We selected the tumour samples with lowest ACTA2 expression levels (lower quartile), since these samples should be the least contaminated by stromal content:

```
#categorise ACTA2 expression levels into 4 quantile groups:
ACTA2quartiles <- cut (TCGA.GeneExpMat[,"ACTA2"],
                      breaks = quantile (TCGA.GeneExpMat[,"ACTA2"],
                      c (0, .25, .5, .75, 1)),
                      include.lowest = TRUE)
summary(ACTA2quartiles)

##   [-2.95,-0.958] (-0.958,-0.0429]  (-0.0429,0.947]     (0.947,2.63]
##              123              122              122              122

#get subgroup of TCGA samples with the lowest ACTA2 levels
TCGA.lowACTA2 <- TCGA.GeneExpMat[
                  ACTA2quartiles==levels(ACTA2quartiles)[1],]

#plot histogram of ACTA2 expression:
par(mfrow=c(1,3))
hist(TCGA.GeneExpMat[,"PTEN"], xlab="PTEN gene expression",
     breaks=20, main="All tumors",col="grey")
hist(TCGA.lowACTA2[,"PTEN"], xlab="PTEN gene expression",
     breaks=20, main="Low ACTA2 tumours",col="grey")

#Plot density histogram
plot(density(TCGA.GeneExpMat[,"PTEN"]), xlab="PTEN gene expression",
     lwd=2,main="PTEN gene expression")
lines(density(TCGA.lowACTA2[,"PTEN"]),col="red",lwd=2)
legend("topleft", legend=c("All","lowACTA2"),col=c("black","red"),
       lty=1,lwd=2,cex=0.7)
```

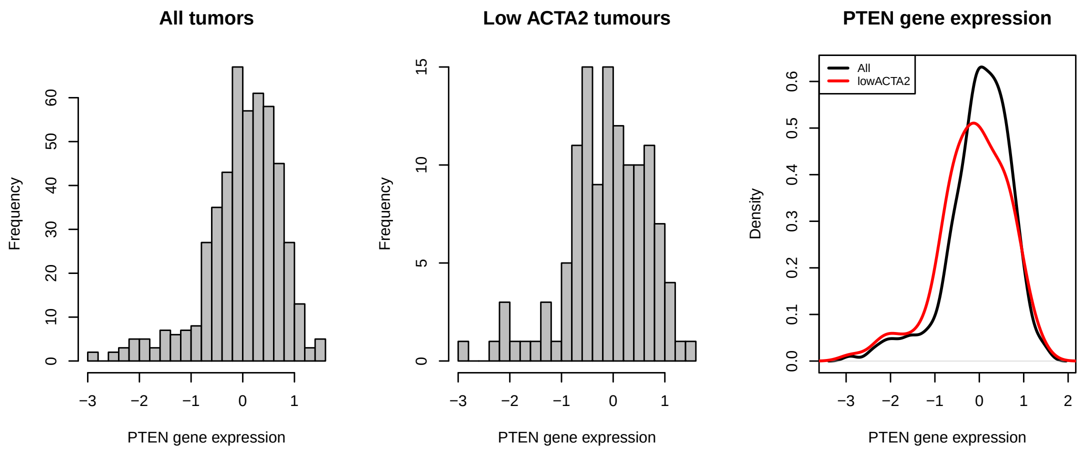

3.6 Differential expression analysis [Fig. 1E]

Here, we preformed a differential gene expression analysis between PTEN-high and PTEN-low tumours using both:

1. the complete set of TCGA samples (N= 489)
2. the subset of tumour samples with low ACTA2 expression (N= 123)

To compare PTEN-high and PTEN-low tumours we categorized the PTEN expression levels into quartiles and the top and bottom quartiles were used for differentially expression (DE) analysis using the `limma` package:

```
#Wrapper function around limma analysis
getDEgenes=function(ExpMat)
  {
  #Categorise PTEN expression levels into three groups
  #This vector will describe the labels in the columns of the dataset
  PTENgroups <- cut(ExpMat[,"PTEN"],
                    breaks = quantile (ExpMat[,"PTEN"],
                    c (0, .25, .75, 1)),include.lowest = TRUE)
  levels(PTENgroups) <- c("bottom","medium","top")

  #Create the design matrix
  design <- model.matrix(~ -1+PTENgroups)
  colnames(design) <- levels(PTENgroups)
  levels(design) <-  levels(PTENgroups)

  #Fit the linear model and construct the contrast matrix
  modelfit <- lmFit(t(ExpMat), design)
```

```
  contrast.matrix <- makeContrasts(top-bottom, levels=design)

  #compute estimated coefficients and standard errors for contrasts
  fit <- contrasts.fit(modelfit, contrast.matrix)
  fit <- eBayes(fit)
  fit$PTENgroups <- PTENgroups
  return(fit)
  }

#Differential expression analysis
fit.alltumors <-  getDEgenes(TCGA.GeneExpMat)
fit.lowACTA2 <-   getDEgenes(TCGA.lowACTA2)
```

Differentially expressed genes (DEGs) were selected after FDR correction for multiple testing and using a p-value cutoff of 0.05:

```
DE.alltumors <- topTable(fit.alltumors, coef=1, adjust="fdr",
                              sort.by="B", number=ncol(TCGA.GeneExpMat))

dim(DE.alltumors[DE.alltumors$adj.P.Val<=0.05,])

## [1] 1735    6
```

```
DE.lowACTA2 <- topTable(fit.lowACTA2, coef=1, adjust="fdr",
                              sort.by="B", number=ncol(TCGA.lowACTA2))

dim(DE.lowACTA2[DE.lowACTA2$adj.P.Val<=0.05,])

## [1] 86  6
```

The top 50 DEGs were selected for further unsupervised hierarchical clustering and visualization using the `made4` package. Unsupervised hierarchical clustering was performed the ward's method and a distance metrics based on the Pearson correlation coefficient :

```
topgenes <- topTable(fit.alltumors, coef=1, adjust="fdr",
                     sort.by="B", number=50)
toptable <- t(TCGA.GeneExpMat)[rownames(topgenes),]
heatplot(toptable, classvec=fit.alltumors$PTENgroups,
         classvecCol=c("blue","gray","red"),
         main = "Figure 1E: Top 50 DEGs\n(All tumours)",
         method="ward", cex.main=0.6)
```

```
## [1] "Data (original) range:  -4.73 3.66"
## [1] "Data (scale) range:  -6.44 6.68"
## [1] "Data scaled to range:  -3 3"
##      Class    Color
## [1,] "bottom" "blue"
## [2,] "medium" "gray"
## [3,] "top"    "red"
```

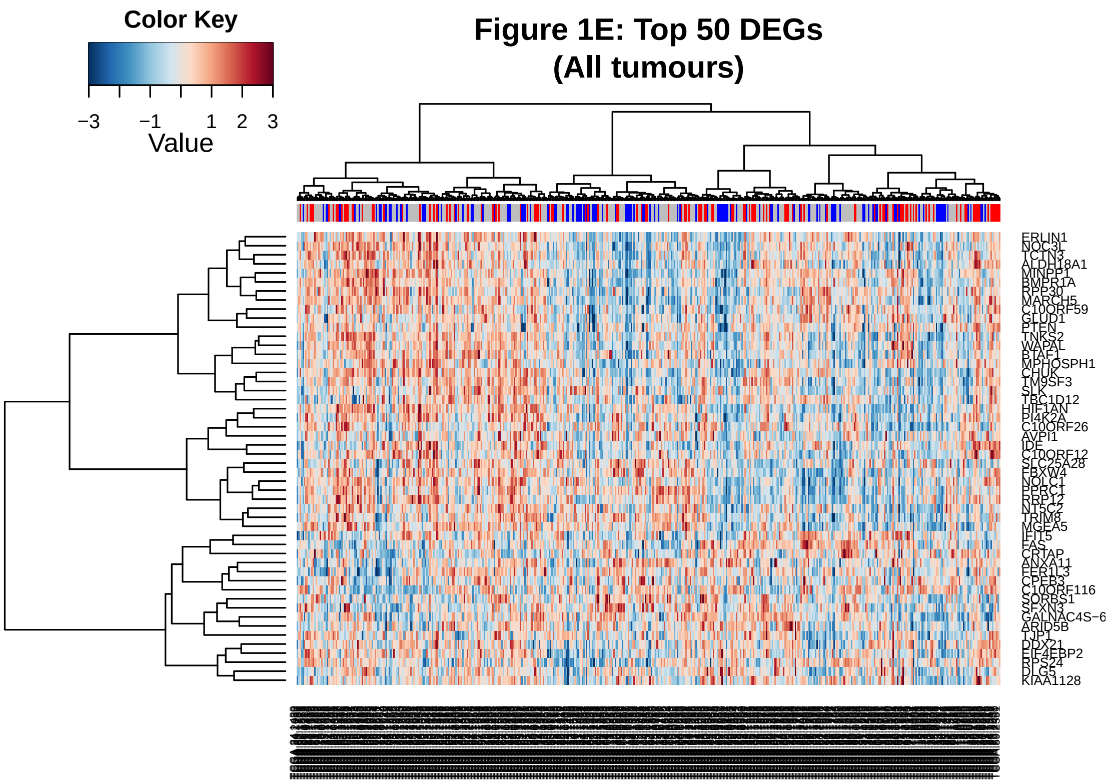


```
topgenes <- topTable(fit.lowACTA2, coef=1, adjust="fdr",
                     sort.by="B", number=50)
toptable <- t(TCGA.lowACTA2)[rownames(topgenes),]
heatplot(toptable, classvec=fit.lowACTA2$PTENgroups,
         classvecCol=c("blue","gray","red"),
         main="Figure 1E: Top 50 DEGs\n(Low ACTA2 tumours)",
         method="ward")
```

```
## [1] "Data (original) range:  -2.89 5.78"
## [1] "Data (scale) range:  -3.48 4.33"
## [1] "Data scaled to range:  -3 3"
##      Class    Color
## [1,] "bottom" "blue"
```

```
## [2,] "medium" "gray"
## [3,] "top"    "red"
```

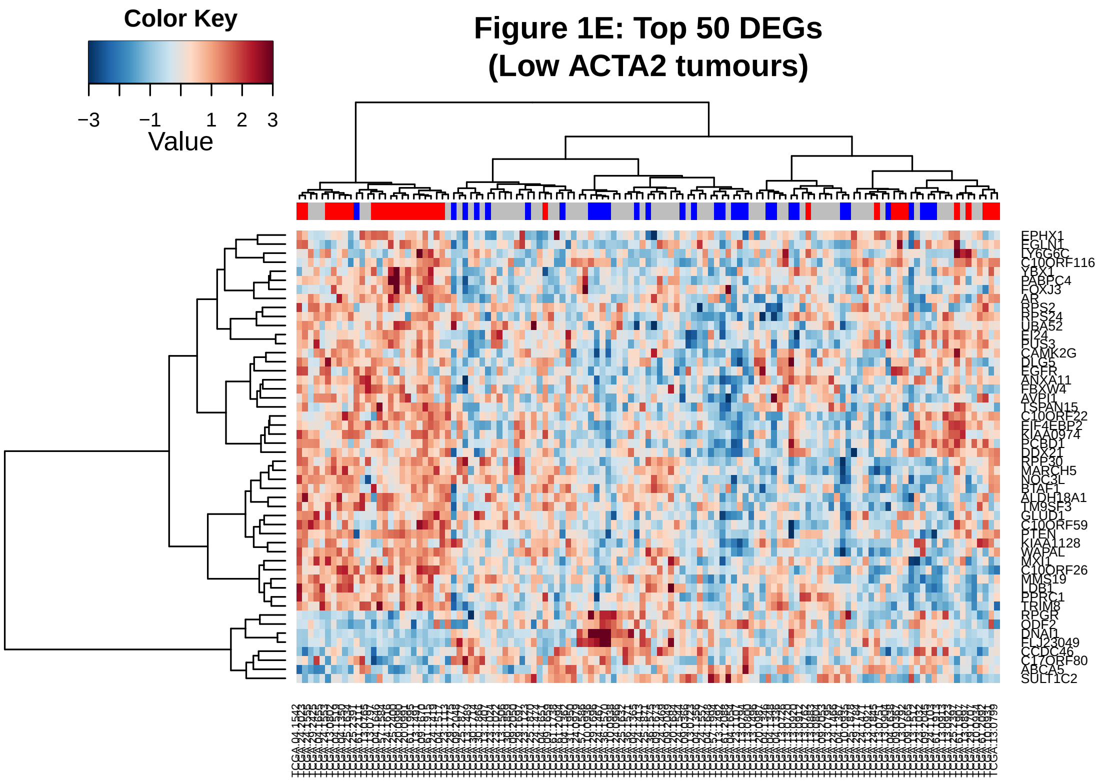

3.7 Low vs High PTEN expression is confounded by stroma [Fig. 1F]

We assessed whether the differentially expressed genes between high PTEN and low PTEN were indeed enriched for stromal genes using the Yoshihara signature. We verify that in the all cohort of tumours, tumours with high PTEN expression were enriched for stromal genes and that the low ACTA2 subset doesn't show this enrichment.

```
## Specify the dataset
DE_valsAll=cbind("ID"=rownames(DE.alltumors),
                 DE.alltumors[,c("t","adj.P.Val")])
DE_valsQuart=cbind("ID"=rownames(DE.lowACTA2),
                   DE.lowACTA2[,c("t","adj.P.Val")])

# Run the function
par(mfrow=c(1,2))
PlotStromalContribution(DE_valsAll, YSig,
                        nperm=1000, ylim=c(-5,5),
                        main=paste("All tumours (N=",
```

```
                                         nrow(TCGA.GeneExpMat),") samples",
                                         sep=""))
PlotStromalContribution(DE_valsQuart,YSig,
                        ylim=c(-5,5), nperm=1000,
                        main=paste("Low ACTA2 tumours (N=",
                                   nrow(TCGA.lowACTA2),") samples",
                                   sep=""))
```

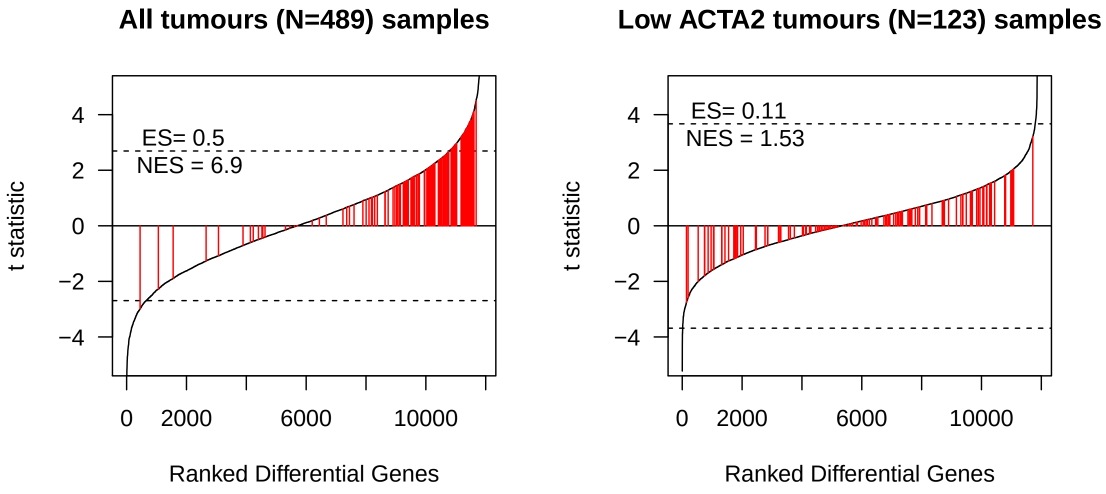

The t statistic for genes were ranked in increasing order, and genes from the Yoshihara signature are shown in red. The left plot (using the raw data) shows an enrichment for stromal genes, suggesting that these results are confounded by stromal content. The right plot (using only low ACTA2 samples) illustrates a more random distribution of stromal markers. None of these had a significant p value (indicated by the dotted lines, BH adjusted p.value equal to 0.05), suggesting that stromal content will not be a confounding factor in analysis of this sub-population.

4 Survival differences between PTEN-high and PTEN-low in HGSC

4.1 Survival differences between PTEN-high and PTEN-low in HGSC from the SEARCH and Nottingham cohorts [Fig. 2B, 2C, S2B]

Immunofluorescence and immunohistochemistry for PTEN was carried out on the SEARCH TMAs. Immunohistochemistry for PTEN was carried out on the NOT TMA. The primary PTEN antibody (Cell signalling - PTEN Clone 138G6) was previously validated (Martins et al., 2012). PTEN expression, either using IF or IHC, was scored from 1 to 4 representing negative; weak; heterogeneous or positive staining, respectively.

```
table(SEARCH$PTEN.IF, useNA = "always")

##
##      negative          weak heterogeneous      positive          <NA>
##           144           138            64           112            58

table(SEARCH$PTEN.IHC, useNA = "always")

##
##      negative          weak heterogeneous      positive          <NA>
##           126           137            55           137            61

table(NOT$PTEN.IHC, useNA = "always")

##
##      negative          weak heterogeneous      positive          <NA>
##            69           123            38           199            78
```

To investigate whether PTEN status is correlated with patients' survival we have plotted the Kaplan-Meier estimate of the survivor function for different PTEN staining scores obtained in HGSCs.

```
#Prepare SEARCH and NOT datasets for the plots below
colors = c("darkblue","lightblue","darkgray","red")
Ds <- subset(SEARCH, HISTOLOGY == "HGSC")
Dn <- subset(NOT, HISTOLOGY == "HGSC")
```

We plotted the Kaplan-Meier estimate of the survivor function in each cohort:

```
#SEARCH cohort
#Create the survival object
ss <- Surv(time=Ds$Months.To.Entry,time2=Ds$Month.To.Status,
          event=Ds$Vital.Status, type="counting")
```

```
#Survival curve for the SEARCH cohort and IF PTEN staining
par(mfrow=c(1,3))
plot(survfit(ss ~ Ds$PTEN.IF), col=colors,
     xlab="Overall survival [months]",ylab="Survival Probability",
     lwd=3,las=1,main="SEARCH Cohort (PTEN.IF)")
#add legend: number of patients in each group and in brackets the number of deaths
legendtext = paste(levels(Ds$PTEN.IF),": ",table(Ds$PTEN.IF),
                   "(",table(Ds$PTEN.IF[Ds$Vital.Status==1]),")",sep="")
legend("topright",col=colors, legend=legendtext,lty=1)

#Survival curve for the SEARCH cohort and IHC PTEN staining
plot(survfit(ss ~ Ds$PTEN.IHC), col=colors,
     xlab="Overall survival [months]",ylab="Survival Probability",
     lwd=3,las=1,main="SEARCH Cohort (PTEN.IHC)")
#add legend: number of patients in each group and in brackets the number of deaths
legendtext = paste(levels(Ds$PTEN.IHC),": ",table(Ds$PTEN.IHC),
                   "(",table(Ds$PTEN.IHC[Ds$Vital.Status==1]),")",sep="")
legend("topright",col=colors, legend=legendtext,lty=1)

#Survival curve for the NOT cohort and IHC PTEN staining
ss <- Surv(Dn$survival.months,Dn$Vital.Status)
plot(survfit(ss ~ Dn$PTEN.IHC), col=colors,
     xlab="Overall survival [months]",ylab="Survival Probability",
     lwd=3,las=1,main="NOT Cohort (PTEN.IHC)")
#add legend: number of patients in each group and in brackets the number of deaths
legendtext = paste(levels(Dn$PTEN.IHC),": ",table(Dn$PTEN.IHC),
                   "(",table(Dn$PTEN.IHC[Dn$Vital.Status==1]),")",sep="")
legend("topright",col=colors, legend=legendtext,lty=1)
```

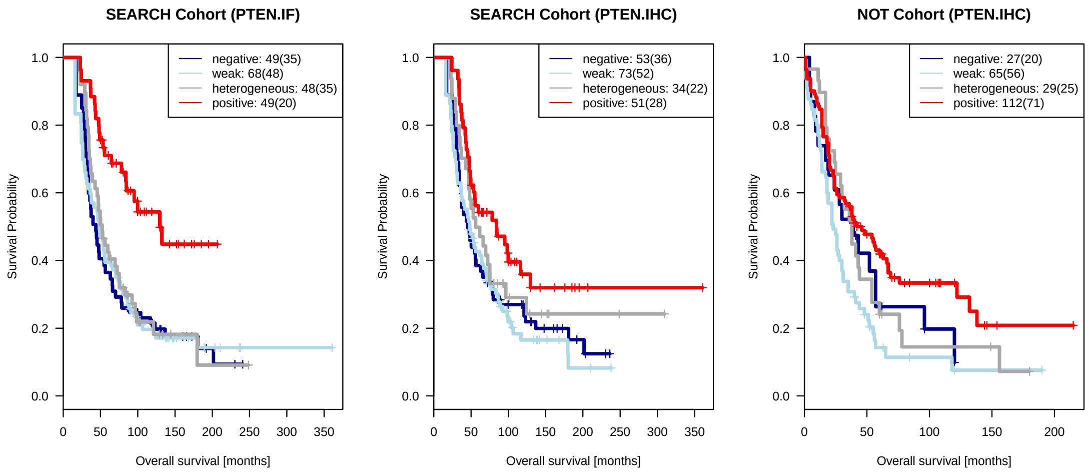

We converted the PTEN staining scores into "PTEN high" vs "PTEN low" (encoded in the PTENstatus2 column) and plotted the Kaplan-Meier survival curves. The hazard (risk) for "PTEN high" vs "PTEN low" tumours was estimated using the Cox-hazard regression model implemented in the function getsurvival:

```
#Convert PTEN scoring into "PTENlow", "PTENhigh"
Ds <- subset(SEARCH, HISTOLOGY == "HGSC")
Dn <- subset(NOT, HISTOLOGY == "HGSC")
labelvect2 <- c("negative"="PTENlow","weak"="PTENlow",
                "heterogeneous"="PTENlow","positive"="PTENhigh")
Ds$PTENstatus2.IF <- factor(labelvect2[Ds$PTEN.IF],levels=c("PTENlow","PTENhigh"))
Ds$PTENstatus2.IHC <- factor(labelvect2[Ds$PTEN.IHC],levels=c("PTENlow","PTENhigh"))
Dn$PTENstatus2.IHC <- factor(labelvect2[Dn$PTEN.IHC],levels=c("PTENlow","PTENhigh"))

#SEARCH cohort
ss1 <- Surv(time=Ds$Months.To.Entry,time2=Ds$Month.To.Status,
           event=Ds$Vital.Status, type="counting")
par(mfrow=c(2,2))
survIF.SEARCH <- getsurvival(ss1, Ds$Vital.Status, Ds$PTENstatus2.IF,
                     title="Figure 2B:\nSEARCH Cohort (PTEN.IF)")

survIHC.SEARCH <- getsurvival(ss1, Ds$Vital.Status, Ds$PTENstatus2.IHC,
                     title="SEARCH Cohort (PTEN.IHC)")
#NOT cohort
ss2 <- Surv(Dn$survival.months, Dn$Vital.Status)
survIHC.NOT <- getsurvival(ss2, Dn$Vital.Status, Dn$PTENstatus2.IHC,
                     title="Figure 2C:\nNOT Cohort (PTEN.IHC)")

#Combined (PTEN.IHC only)
combined <- rbind(data.frame("t1"=Ds$Months.To.Entry, "t2"=Ds$Month.To.Status,
                             "Vital.Status"=Ds$Vital.Status,
                             "PTENstatus2.IHC"=Ds$PTENstatus2.IHC,
                             "Age.At.Diagnosis"=Ds$Age.At.Diagnosis,
                             "Grade"=as.numeric(Ds$Grade),
                             "Stage"=as.numeric(Ds$Stage), "study"=rep(0,nrow(Ds))),
                  data.frame("t1"=rep(0,nrow(Dn)), "t2"=Dn$survival.months,
                             "Vital.Status"=Dn$Vital.Status,
                             "PTENstatus2.IHC"=Dn$PTENstatus2.IHC,
                             "Age.At.Diagnosis"=Dn$Age.At.Diagnosis,
                             "Grade"=as.numeric(Dn$Grade),
                             "Stage"=as.numeric(Dn$Stage),
                             "study"=rep(1,nrow(Dn))))
ss.comb <- Surv(time=combined$t1,time2=combined$t2,
           event=combined$Vital.Status, type="counting")
```

```
survIHC.combined <- getsurvival(ss.comb, combined$Vital.Status,
                                combined$PTENstatus2.IHC,
                                title="Figure S2B:\nSEARCH+NOT Cohorts (PTEN.IHC)")
```

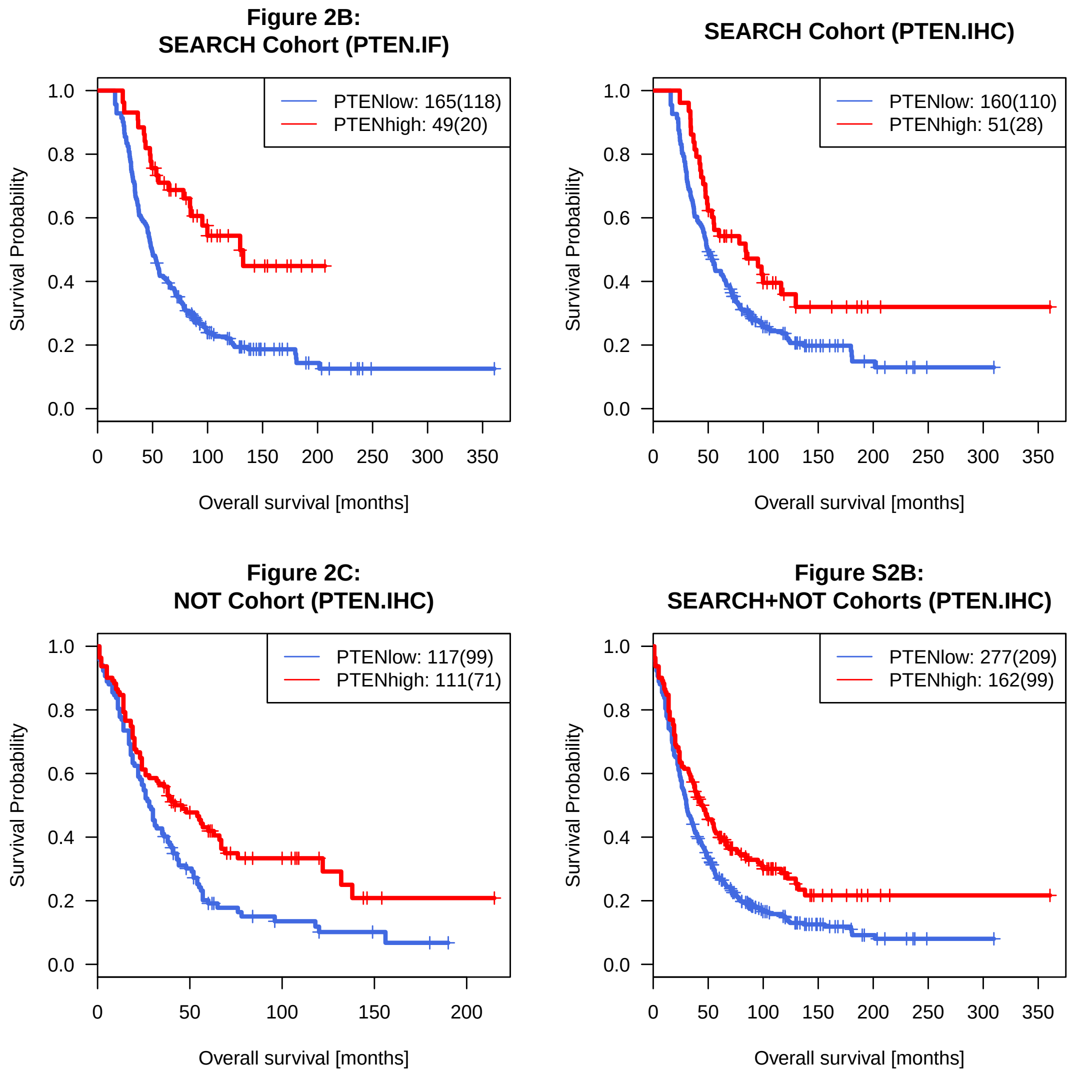


```
#add study to the cox regression model
survIHC.combined <- coxph(ss.comb ~ combined$study + combined$PTENstatus2.IHC)
```

The Cox regression analysis shows a higher hazard (risk of death), and thus worse prognosis, for subjects with lower PTEN staining. However, although the p-value is significant for the IF measurements, for IHC staining $P \not< 0{\cdot}05$ in the SEARCH cohort.

```r
summary(survIF.SEARCH)

## Call:
## coxph(formula = ss ~ predictors)
##
##   n= 214, number of events= 138
##    (31 observations deleted due to missingness)
##
##                         coef exp(coef) se(coef)     z Pr(>|z|)
## predictorsPTENhigh -0.833     0.435    0.242 -3.44  0.00058 ***
## ---
## Signif. codes:  0 '***' 0.001 '**' 0.01 '*' 0.05 '.' 0.1 ' ' 1
##
##                     exp(coef) exp(-coef) lower .95 upper .95
## predictorsPTENhigh     0.435        2.3      0.27     0.698
##
## Concordance= 0.571  (se = 0.02 )
## Rsquare= 0.065   (max possible= 0.997 )
## Likelihood ratio test= 14.3  on 1 df,   p=0.000156
## Wald test            = 11.8  on 1 df,   p=0.000576
## Score (logrank) test = 12.6  on 1 df,   p=0.000396

summary(survIHC.SEARCH)

## Call:
## coxph(formula = ss ~ predictors)
##
##   n= 211, number of events= 138
##    (34 observations deleted due to missingness)
##
##                         coef exp(coef) se(coef)     z Pr(>|z|)
## predictorsPTENhigh -0.354     0.702    0.212 -1.67    0.095 .
## ---
## Signif. codes:  0 '***' 0.001 '**' 0.01 '*' 0.05 '.' 0.1 ' ' 1
##
##                     exp(coef) exp(-coef) lower .95 upper .95
## predictorsPTENhigh     0.702       1.42     0.463      1.06
##
## Concordance= 0.528  (se = 0.02 )
## Rsquare= 0.014   (max possible= 0.997 )
## Likelihood ratio test= 2.98  on 1 df,   p=0.0841
## Wald test            = 2.79  on 1 df,   p=0.0949
## Score (logrank) test = 2.82  on 1 df,   p=0.0932
```

```r
summary(survIHC.NOT)

## Call:
## coxph(formula = ss ~ predictors)
##
##   n= 228, number of events= 170
##    (48 observations deleted due to missingness)
##
##                        coef exp(coef) se(coef)     z Pr(>|z|)
## predictorsPTENhigh -0.482     0.618    0.156 -3.08   0.0021 **
## ---
## Signif. codes:  0 '***' 0.001 '**' 0.01 '*' 0.05 '.' 0.1 ' ' 1
##
##                    exp(coef) exp(-coef) lower .95 upper .95
## predictorsPTENhigh     0.618       1.62     0.455     0.839
##
## Concordance= 0.551  (se = 0.021 )
## Rsquare= 0.041   (max possible= 0.999 )
## Likelihood ratio test= 9.64  on 1 df,   p=0.0019
## Wald test            = 9.48  on 1 df,   p=0.00208
## Score (logrank) test = 9.66  on 1 df,   p=0.00188

summary(survIHC.combined)

## Call:
## coxph(formula = ss.comb ~ combined$study + combined$PTENstatus2.IHC)
##
##   n= 439, number of events= 308
##    (82 observations deleted due to missingness)
##
##                                      coef exp(coef) se(coef)     z Pr(>|z|)
## combined$study                      0.209     1.232    0.136  1.53  0.12530
## combined$PTENstatus2.IHCPTENhigh -0.443     0.642    0.127 -3.50  0.00047
##
## combined$study
## combined$PTENstatus2.IHCPTENhigh ***
## ---
## Signif. codes:  0 '***' 0.001 '**' 0.01 '*' 0.05 '.' 0.1 ' ' 1
##
##                                  exp(coef) exp(-coef) lower .95 upper .95
## combined$study                       1.232      0.812     0.943     1.609
## combined$PTENstatus2.IHCPTENhigh     0.642      1.557     0.501     0.823
##
## Concordance= 0.558  (se = 0.016 )
```

```
## Rsquare= 0.029   (max possible= 0.999 )
## Likelihood ratio test= 13.1  on 2 df,   p=0.00146
## Wald test            = 12.7  on 2 df,   p=0.00179
## Score (logrank) test = 12.8  on 2 df,   p=0.00168
```

4.2 Multivariate Cox-hazard model

Additionally to the univariate analysis above, which showed significant differences in survival curves between "PTEN high" and "PTEN low" tumours, we used the multivariate Cox-hazard regression to correct for the patients' age, tumour stage and grade:

```
#Multivariate Cox-hazard model for PTEN.IF in the SEARCH chohort
ss1 <- Surv(time=Ds$Months.To.Entry,time2=Ds$Month.To.Status,
           event=Ds$Vital.Status, type="counting")
fitIF.SEARCH <- coxph(ss1 ~ Ds$Age.At.Diagnosis + Ds$Grade +
                        Ds$Stage + Ds$PTENstatus2.IF)

# Multivariate Cox-hazard model for PTEN.IHC in the NOT chohort
ss2 <- Surv(Dn$survival.months, Dn$Vital.Status)
fitIHC.NOT <- coxph(ss2 ~ Dn$Age.At.Diagnosis + Dn$Grade +
                        Dn$Stage + Dn$PTENstatus2.IHC)

#Multivariate Cox-hazard model for PTEN.IHC in the combined SEARCH+NOT chohorts
ss.comb <- Surv(time=combined$t1,time2=combined$t2,
           event=combined$Vital.Status, type="counting")
fitIHC.combined <- coxph(ss.comb ~ combined$study +
                                   combined$Age.At.Diagnosis +
                                   combined$Grade +
                                   combined$Stage +
                                   combined$PTENstatus2.IHC)
```

After adjustment for the other explanatory variables in the model, the PTEN "positive" IF staining is still significantly associated with higher risk:

```
summary(fitIF.SEARCH)

## Call:
## coxph(formula = ss1 ~ Ds$Age.At.Diagnosis + Ds$Grade + Ds$Stage +
##     Ds$PTENstatus2.IF)
##
##   n= 171, number of events= 112
##    (74 observations deleted due to missingness)
##
```

```
##                                coef exp(coef) se(coef)     z Pr(>|z|)
## Ds$Age.At.Diagnosis          0.0193    1.0195   0.0128  1.52     0.13
## Ds$Grade                    -0.2793    0.7563   0.1989 -1.40     0.16
## Ds$Stage                     0.6619    1.9385   0.1241  5.33  9.6e-08 ***
## Ds$PTENstatus2.IFPTENhigh -0.5622    0.5699   0.2744 -2.05     0.04 *
## ---
## Signif. codes:  0 '***' 0.001 '**' 0.01 '*' 0.05 '.' 0.1 ' ' 1
##
##                           exp(coef) exp(-coef) lower .95 upper .95
## Ds$Age.At.Diagnosis           1.020      0.981     0.994     1.045
## Ds$Grade                      0.756      1.322     0.512     1.117
## Ds$Stage                      1.939      0.516     1.520     2.472
## Ds$PTENstatus2.IFPTENhigh     0.570      1.755     0.333     0.976
##
## Concordance= 0.695  (se = 0.029 )
## Rsquare= 0.246   (max possible= 0.997 )
## Likelihood ratio test= 48.2  on 4 df,   p=8.59e-10
## Wald test            = 43  on 4 df,   p=1.04e-08
## Score (logrank) test = 45.7  on 4 df,   p=2.87e-09
```

```
anova(fitIF.SEARCH)
```

```
## Analysis of Deviance Table
##  Cox model: response is ss1
## Terms added sequentially (first to last)
##
##                       loglik Chisq Df Pr(>|Chi|)
## NULL                    -489
## Ds$Age.At.Diagnosis     -485  6.21  1      0.013 *
## Ds$Grade                -485  0.80  1      0.370
## Ds$Stage                -467 36.44  1    1.6e-09 ***
## Ds$PTENstatus2.IF       -464  4.74  1      0.029 *
## ---
## Signif. codes:  0 '***' 0.001 '**' 0.01 '*' 0.05 '.' 0.1 ' ' 1
```

In the NOT cohort:

```
summary(fitIHC.NOT)
```

```
## Call:
## coxph(formula = ss2 ~ Dn$Age.At.Diagnosis + Dn$Grade + Dn$Stage +
##     Dn$PTENstatus2.IHC)
##
##   n= 159, number of events= 125
```

```
##    (117 observations deleted due to missingness)
##
##                                  coef exp(coef) se(coef)     z Pr(>|z|)
## Dn$Age.At.Diagnosis         0.01043   1.01048  0.00891  1.17   0.2417
## Dn$Grade                   -0.19563   0.82231  0.50612 -0.39   0.6991
## Dn$Stage                    0.75605   2.12984  0.13229  5.72  1.1e-08 ***
## Dn$PTENstatus2.IHCPTENhigh -0.57561   0.56236  0.18711 -3.08   0.0021 **
## ---
## Signif. codes:  0 '***' 0.001 '**' 0.01 '*' 0.05 '.' 0.1 ' ' 1
##
##                            exp(coef) exp(-coef) lower .95 upper .95
## Dn$Age.At.Diagnosis            1.010       0.99     0.993     1.028
## Dn$Grade                       0.822       1.22     0.305     2.217
## Dn$Stage                       2.130       0.47     1.643     2.760
## Dn$PTENstatus2.IHCPTENhigh     0.562       1.78     0.390     0.811
##
## Concordance= 0.687  (se = 0.029 )
## Rsquare= 0.274   (max possible= 0.999 )
## Likelihood ratio test= 50.8  on 4 df,   p=2.44e-10
## Wald test            = 46.5  on 4 df,   p=1.94e-09
## Score (logrank) test = 47.5  on 4 df,   p=1.21e-09
```

```
anova(fitIHC.NOT)
```

```
## Analysis of Deviance Table
##  Cox model: response is ss2
## Terms added sequentially (first to last)
##
##                      loglik Chisq Df Pr(>|Chi|)
## NULL                   -551
## Dn$Age.At.Diagnosis    -547  7.52  1     0.0061 **
## Dn$Grade               -547  0.34  1     0.5580
## Dn$Stage               -530 33.36  1    7.7e-09 ***
## Dn$PTENstatus2.IHC     -525  9.59  1     0.0020 **
## ---
## Signif. codes:  0 '***' 0.001 '**' 0.01 '*' 0.05 '.' 0.1 ' ' 1
```

The PTEN status obtained with IHC staining is also significantly associated with higher risk:

```
summary(fitIHC.combined)
```

```
## Call:
## coxph(formula = ss.comb ~ combined$study + combined$Age.At.Diagnosis +
```

```
##     combined$Grade + combined$Stage + combined$PTENstatus2.IHC)
##
##   n= 329, number of events= 239
##    (192 observations deleted due to missingness)
##
##                                   coef exp(coef) se(coef)     z
## combined$study                 0.36552   1.44127  0.16972  2.15
## combined$Age.At.Diagnosis      0.01404   1.01414  0.00716  1.96
## combined$Grade                -0.24252   0.78465  0.18786 -1.29
## combined$Stage                 0.68091   1.97568  0.08869  7.68
## combined$PTENstatus2.IHCPTENhigh -0.40132   0.66944  0.14946 -2.69
##                               Pr(>|z|)
## combined$study                  0.0313 *
## combined$Age.At.Diagnosis       0.0499 *
## combined$Grade                  0.1967
## combined$Stage                 1.6e-14 ***
## combined$PTENstatus2.IHCPTENhigh   0.0073 **
## ---
## Signif. codes:  0 '***' 0.001 '**' 0.01 '*' 0.05 '.' 0.1 ' ' 1
##
##                               exp(coef) exp(-coef) lower .95 upper .95
## combined$study                    1.441      0.694     1.033     2.010
## combined$Age.At.Diagnosis         1.014      0.986     1.000     1.028
## combined$Grade                    0.785      1.274     0.543     1.134
## combined$Stage                    1.976      0.506     1.660     2.351
## combined$PTENstatus2.IHCPTENhigh     0.669      1.494     0.499     0.897
##
## Concordance= 0.688  (se = 0.02 )
## Rsquare= 0.232   (max possible= 0.999 )
## Likelihood ratio test= 86.9  on 5 df,   p=0
## Wald test            = 78.9  on 5 df,   p=1.44e-15
## Score (logrank) test = 81.1  on 5 df,   p=4.44e-16
```

```
anova(fitIHC.combined)
```

```
## Analysis of Deviance Table
##  Cox model: response is ss.comb
## Terms added sequentially (first to last)
##
##                            loglik Chisq Df Pr(>|Chi|)
## NULL                        -1171
## combined$study              -1171  0.13  1     0.7221
## combined$Age.At.Diagnosis   -1164 13.08  1     0.0003 ***
## combined$Grade              -1164  0.06  1     0.7995
```

```
## combined$Stage               -1131 66.25  1     4.4e-16 ***
## combined$PTENstatus2.IHC   -1127  7.42  1      0.0064 **
## ---
## Signif. codes:  0 '***' 0.001 '**' 0.01 '*' 0.05 '.' 0.1 ' ' 1
```

4.3 Correlation between immunofluorescence (IF) and immunohistochemistry (IHC) staining in samples from the SEARCH cohort [Fig. S2A]

Although PTEN expression could be easier to quantitate using immunofluorescence (IF) rather than immunohistochemistry (IHC), the former is not as widely used by pathologists in clinical practice. Therefore, we performed both staining techniques, IHC and IF. The intensity scores for each sample can be found in the `SEARCH` data set under `PTEN.IF` and `PTEN.IHC`:

```
table(SEARCH$PTEN.IF)

##
##      negative          weak heterogeneous      positive
##           144           138            64           112

table(SEARCH$PTEN.IHC)

##
##      negative          weak heterogeneous      positive
##           126           137            55           137
```

We confirmed that the IHC and IF staining scores are well correlated:

```
data2plot <- subset(SEARCH, PTEN.IHC != "heterogeneous" &
                      PTEN.IF != "heterogeneous" ,
                    select = c("HISTOLOGY","PTEN.IHC","PTEN.IF"))
data2plot$PTEN.IHC <- factor(data2plot$PTEN.IHC)
data2plot$PTEN.IF <- factor(data2plot$PTEN.IF)

#Plot the correlation between IF and IHC staining scores
d <- data2plot[data2plot$HISTOLOGY == "HGSC",c("PTEN.IHC","PTEN.IF")]
plotcorrImage(d)
p <- chisq.test(table(d)) #Preform chi-square test and get p-value
p <- round(p$p.value*10^32, 2)/10^32 #round
title(main=paste("Figure S2A:Correlation between\n",
                 "IF and IHC staining scores\n","p-value=",p),cex.main=.9)
```

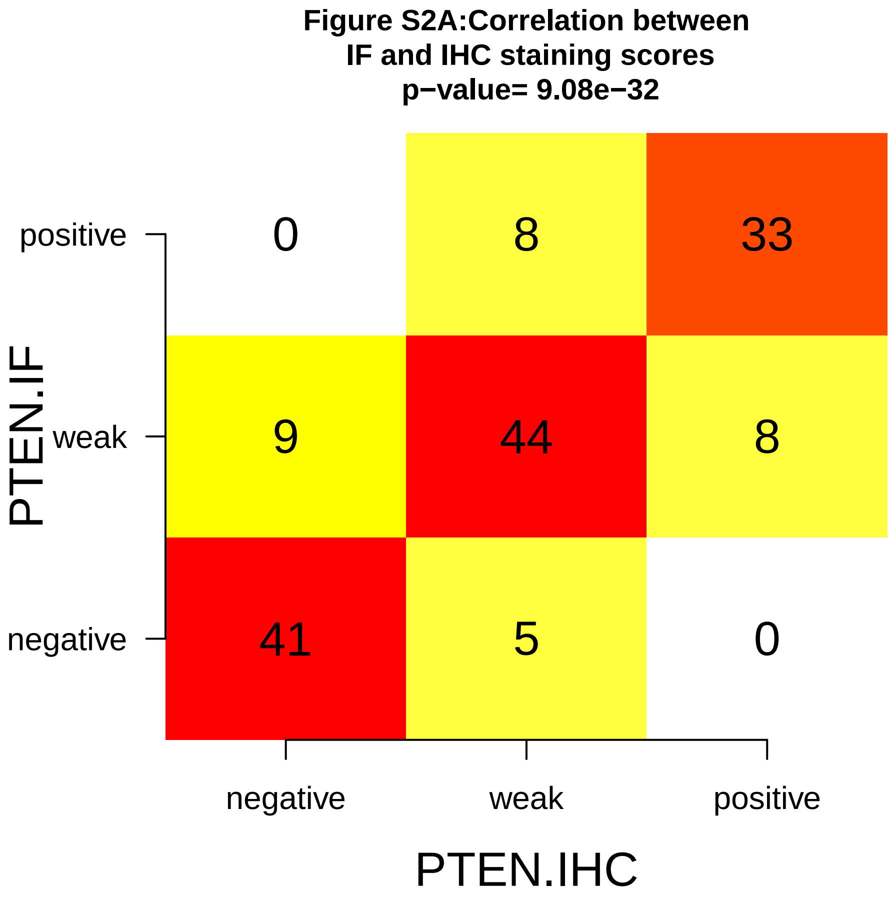

4.4 BRCA germline mutation against PTEN expression in SEARCH cohort

```
# create germline mutation table and see whether there is a relationship between BRCA muta
MutTable=table(SEARCH$Germline.BRCA.mut, SEARCH$PTEN.IHC)
MutTable

##
##              negative weak heterogeneous positive
##    BRCA1            1    8             0        1
##    BRCA2            3    7             3        4
##    Failed           1    2             0        2
##    NotTested       19   11            11       27
##    Tested         102  109            41      103

MutTable2=matrix(c(sum(MutTable["BRCA1", c("negative", "weak") ]),
            sum(MutTable["BRCA1", c("positive", "heterogeneous") ]),
```

```
            sum(MutTable[c("BRCA2", "Tested"), c("negative", "weak") ]),
            sum(MutTable[c("BRCA2", "Tested"), c("positive", "heterogeneous") ])),
                 nrow=2, ncol=2, dimnames=list(c("BRCA1", "wt"), c("Weak", "High")))

fisher.test(MutTable2)

##
##  Fisher's Exact Test for Count Data
##
## data:  MutTable2
## p-value = 0.05649
## alternative hypothesis: true odds ratio is not equal to 1
## 95 percent confidence interval:
##    0.8343 270.9669
## sample estimates:
## odds ratio
##      6.128
```

5 Exploring the relationship between PTEN loss with PTEN DNA methylation and copy number alterations

Using the TCGA set of 489 high-grade serous ovarian cancers, we investigated the relationship between PTEN expression status, copy number alterations and methylation status. The methylation and CNA data for the 489 ovarian cancer samples can be found in the cbiodata table:

```
dim(cbiodata)

## [1] 489   4

head(cbiodata)

##              PTEN.methyl PTEN.cnv  AR PTEN
## TCGA-04-1331     0.07449   Homdel NaN  NaN
## TCGA-04-1332     0.14699  Hetloss NaN  NaN
## TCGA-04-1336     0.05829  Diploid NaN  NaN
## TCGA-04-1337     0.08302  Diploid NaN  NaN
## TCGA-04-1338     0.04445     Gain NaN  NaN
## TCGA-04-1341     0.08674  Diploid NaN  NaN
```

First, we need to merge the cbiodata with the PTEN gene expression data within `TCGA.GeneExpMat[,"PTEN"]`:

```
# Merge gene expression data with methylation data
cbiodata <- merge(cbiodata, TCGA.GeneExpMat[, "PTEN"], by = "row.names")
rownames(cbiodata) <- cbiodata$Row.names
colnames(cbiodata)[ncol(cbiodata)] <- "PTEN.exp"
head(cbiodata)

##                 Row.names PTEN.methyl PTEN.cnv  AR PTEN PTEN.exp
## TCGA-04-1331 TCGA-04-1331     0.07449   Homdel NaN  NaN -2.23625
## TCGA-04-1332 TCGA-04-1332     0.14699  Hetloss NaN  NaN  0.24500
## TCGA-04-1336 TCGA-04-1336     0.05829  Diploid NaN  NaN  0.04021
## TCGA-04-1337 TCGA-04-1337     0.08302  Diploid NaN  NaN  0.02909
## TCGA-04-1338 TCGA-04-1338     0.04445     Gain NaN  NaN  0.38571
## TCGA-04-1341 TCGA-04-1341     0.08674  Diploid NaN  NaN  0.15540
```

5.1 Relationship between PTEN methylation and expression levels in the TCGA samples [Fig. 3B]

By investigating PTEN DNA-methylation levels, we found that PTEN methylation is significantly different between PTEN high and PTEN low cases:

```
#categorise PTEN expression levels into 4 quantile groups:
groupPTENexp <- cut (cbiodata[,"PTEN.exp"],
                     breaks = quantile (cbiodata[,"PTEN.exp"],
                     c(0, .25, .75, 1)), include.lowest = TRUE)

#plot boxplot PTEN methylation levels per group:
boxplot(cbiodata[,"PTEN.methyl"] ~  groupPTENexp,
        main=paste("Figure 3B: Relationship between PTEN methylation",
        "and expression levels in the TCGA samples", sep="\n"),
        ylab="PTEN Methylation", xlab="PTEN expression",
        names=c("low","medium","high"),cex.main=.8)
```

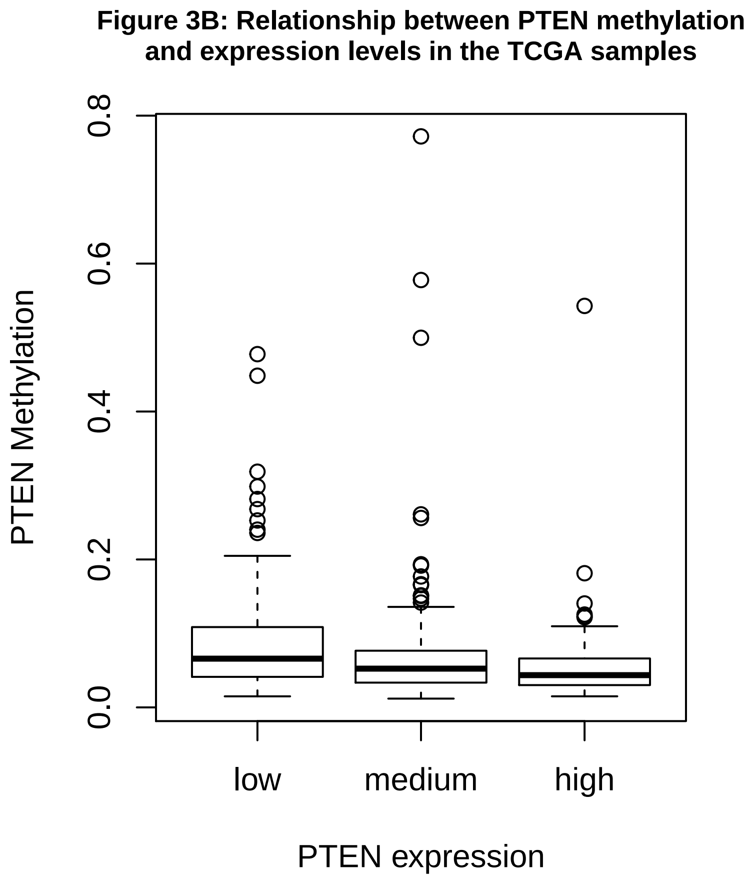


```
#pvalue comparing PTEN high vs PTEN low groups
data2test <- split(cbiodata[,"PTEN.methyl"],groupPTENexp)
names(data2test)

## [1] "[-2.92,-0.322]" "(-0.322,0.495]" "(0.495,1.48]"
```

```
wilcox.test(data2test[[1]], data2test[[3]])

##
##  Wilcoxon rank sum test with continuity correction
##
## data:  data2test[[1]] and data2test[[3]]
## W = 9882, p-value = 1.8e-05
## alternative hypothesis: true location shift is not equal to 0
```

5.2 Distribution of PTEN ploidy within the TCGA dataset [Fig. 3C]

By investigating PTEN copy number alterations, we verified that, besides the 6% tumours with homozygous deletion (30 out of 489 tumours), loss of a single allele was prevalent in the TCGA samples (36%; 174 out of 489 tumours):

```
tt <- table(cbiodata$PTEN.cnv)
par(mar=c(5,6,5,6))
pie(tt,labels=paste(names(tt)," (N=",tt,")",sep=""), cex.main=.9,
    main="Figure 3C: PTEN Copy Number\nwithin the TCGA dataset",
    col=c("red","darkorange","lightgreen","lightblue","dodgerblue"))
```

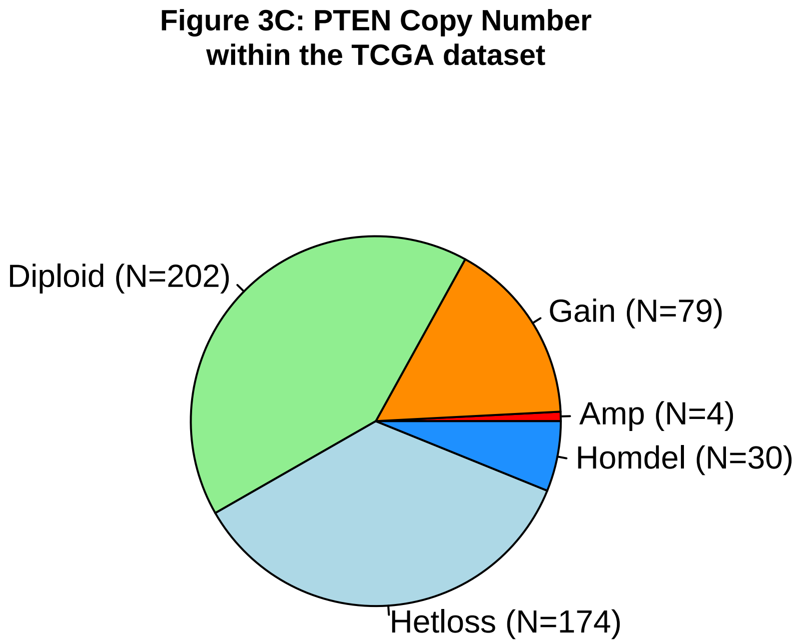

5.3 Relationship between PTEN ploidy and expression levels in the TCGA samples [Fig. 3D]

In order to understand if the hemizygous deletions could have functional consequences, we correlated the copy number alterations with PTEN expression. We found that that PTEN expression is significantly lower when there is only one allele (HetLoss), in comparison with tumours that have at least two wild-type alleles (e.g. Diploid, Gain, Amp).

```
bp <- boxplot(cbiodata$PTEN.exp ~ cbiodata$PTEN.cnv,plot=F)
boxplot(cbiodata$PTEN.exp ~ cbiodata$PTEN.cnv, xlab="PTEN CopyNumber",
        ylab="PTEN Expression", names=bp$names,cex.axis=0.9, cex.main=.8,
        main=paste("Figure 3D: Relationship between PTEN ploidy and",
                   "expression levels in the TCGA samples", sep="\n"))
mtext(at=1:5,text=paste("N=",bp$n), side=1, line=2)
```

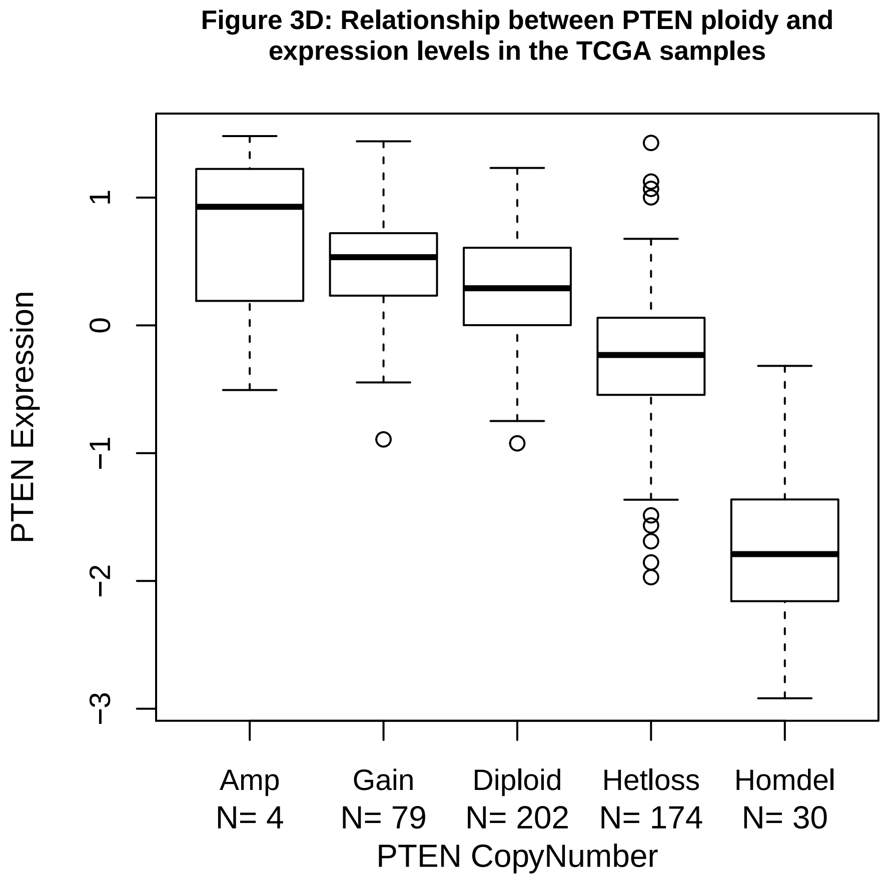

We can test whether the difference is significant:

```
# Test between diploid and Hetloss
t.test(cbiodata$PTEN.exp[cbiodata$PTEN.cnv=="Diploid"],
       cbiodata$PTEN.exp[cbiodata$PTEN.cnv=="Hetloss"])

##
##  Welch Two Sample t-test
##
## data:  cbiodata$PTEN.exp[cbiodata$PTEN.cnv == "Diploid"] and cbiodata$PTEN.exp[cbiodata
## t = 10.48, df = 337.2, p-value < 2.2e-16
## alternative hypothesis: true difference in means is not equal to 0
## 95 percent confidence interval:
##  0.4267 0.6238
## sample estimates:
## mean of x mean of y
##    0.2763   -0.2490
```

```r
# Test between diploid and hom del
t.test(cbiodata$PTEN.exp[cbiodata$PTEN.cnv=="Diploid"],
       cbiodata$PTEN.exp[cbiodata$PTEN.cnv=="Homdel"])

##
##  Welch Two Sample t-test
##
## data:  cbiodata$PTEN.exp[cbiodata$PTEN.cnv == "Diploid"] and cbiodata$PTEN.exp[cbiodata
## t = 17.95, df = 33.66, p-value < 2.2e-16
## alternative hypothesis: true difference in means is not equal to 0
## 95 percent confidence interval:
##  1.811 2.274
## sample estimates:
## mean of x mean of y
##    0.2763   -1.7664
```

5.4 PTEN gene expression within each IHC staining group [Fig. 3F and 3G]

We rescored the 51 images stained for PTEN by Hanrahan et al. (2012) using a four point scale: negative, weak negative, heterogeneous and positive. The new scores are stored in the `Hanrahan2012` object and are summarized below.

```r
table(Hanrahan2012$PTEN.TCGA, useNA = "always")

##
##    neg hetero    pos   <NA>
##      7      8     36     49

table(Hanrahan2012$PTEN, useNA = "always")

##
## negative     weak   hetero positive     <NA>
##        8        9       19       15       49
```

```r
head(Hanrahan2012)

##   OverallSurvival.mos. VITALSTATUS ProgressionFreeStatus
## 1                44.43           1   Recurred/Progressed
## 2                12.10           1   Recurred/Progressed
## 3                 8.26           0          DiseaseFree
## 4                56.59           0          DiseaseFree
## 5                47.70           0          DiseaseFree
```

```
## 6                 70.62           1   Recurred/Progressed
##   ProgressionFreeSurvival..mos.. PlatinumFreeInterval..mos..
## 1                          9.57                         3.7
## 2                          5.97                        -0.8
## 3                          8.26                         3.5
## 4                         56.59                        51.7
## 5                         47.70                        40.5
## 6                         11.51                         6.0
##   PlatinumStatus     PTEN PTEN.TCGA    exp PTEN.genotype           others
## 1      Resistant negative       neg -2.773          homo               wt
## 2      Resistant negative       neg -2.032          hemi       PI3KCA amp
## 3       Tooearly negative       neg -1.688          homo               wt
## 4      Sensitive negative       neg -0.506          hemi               wt
## 5      Sensitive negative       neg -2.798          homo AKT1 amp RB1 del
## 6      Sensitive negative    hetero -0.940          homo       PI3KCA amp
```

We reviewed their images and classifications and applied our own scoring procedure to PTEN IHC staining. The original IHC scores published in Hanrahan et al. (2012) can be found under `Hanrahan2012$PTEN.TCGA` and the scores obtained after our reclassification can be found under PTEN. Note that in `Hanrahan2012$PTEN`, PTEN IHC staining was classified as "negative", "heterogeneous" and "positive" by Hanrahan et al. (2012), while we opted to score IHC staning as "negative", "weak negative", "heterogeneous" and "positive" to further differentiate the tumours based on the intensity of PTEN staining.

Out of the original 36 samples scored as "positive", 21 were reclassified as "heterogeneous" or "weakly positive" according to our scoring procedure:

```
#Figure 3F: comparing PTEN IHC scores between Hanrahan2012
#and our own scoring procedure:
table(Hanrahan2012$PTEN, Hanrahan2012$PTEN.TCGA)

##
##            neg hetero pos
##   negative   7      1   0
##   weak       0      2   7
##   hetero     0      5  14
##   positive   0      0  15

d=subset(Hanrahan2012, select = c("PTEN.TCGA","PTEN"))
plotcorrImage(d)
title(main="Fig 3F: Concordance between TCGA and our classification",
      cex.main=.8)
```

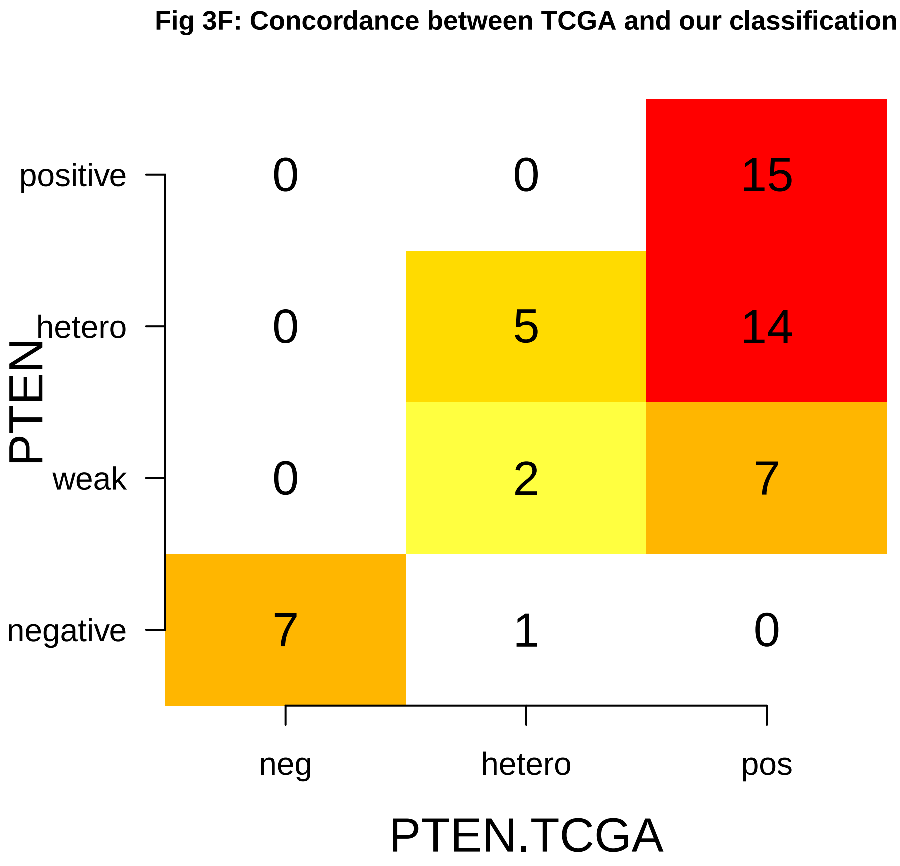

We found that PTEN gene expression correlated well with both classification scores. However, confirming our reclassification criteria, we observe a clear difference in PTEN gene expression between "weak positive" and "positive" samples.

```
par(mfrow=c(1,2))
boplot(Hanrahan2012$exp ~ Hanrahan2012$PTEN,ylab="Expression",
        xlab="PTEN classification (Ours)")
boxplot(Hanrahan2012$exp ~ Hanrahan2012$PTEN.TCGA,ylab="Expression",
        xlab="PTEN classification (TCGA)")
title(main=paste("Figure 3G: Relationship between PTEN expression",
      "and IHC classification scores",sep="\n"), out=TRUE, line=-3)
```

Figure 3G: Relationship between PTEN expression and IHC classification scores

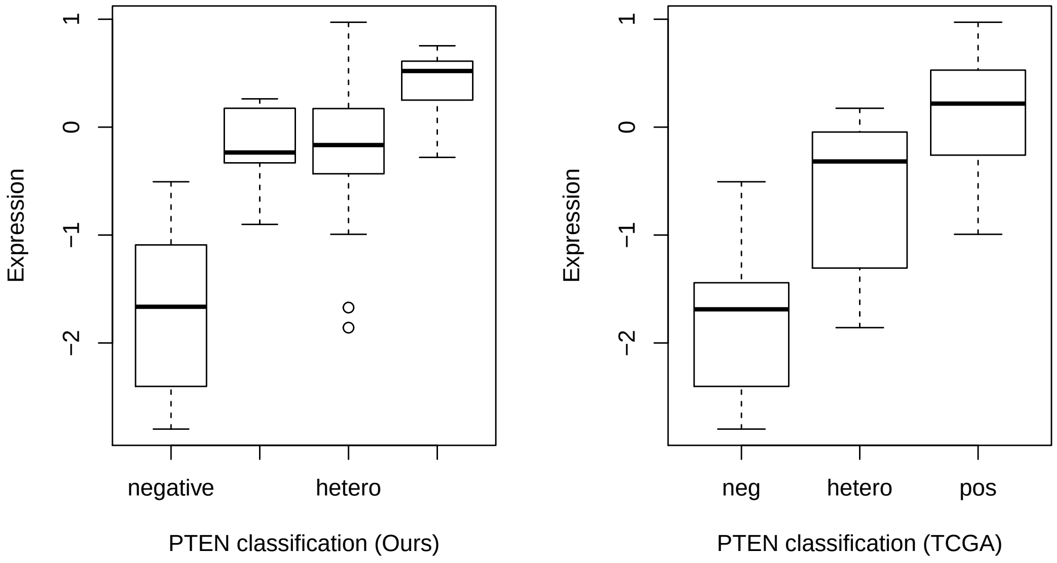

There is a significant difference between PTEN expression in tumours classified as "weak positive" and "positive":

```
group1 <- Hanrahan2012$exp[Hanrahan2012$PTEN == "weak"]
group2 <- Hanrahan2012$exp[Hanrahan2012$PTEN == "positive"]
# NewData: wilcox test weak vs pos
wilcox.test(group1,group2)

##
##  Wilcoxon rank sum test
##
## data:  group1 and group2
## W = 18, p-value = 0.002146
## alternative hypothesis: true location shift is not equal to 0

# NewData: hetero vs positive
wilcox.test(Hanrahan2012$exp[Hanrahan2012$PTEN == "hetero"],
            Hanrahan2012$exp[Hanrahan2012$PTEN == "positive"])

##
##  Wilcoxon rank sum test
##
## data:  Hanrahan2012$exp[Hanrahan2012$PTEN == "hetero"] and Hanrahan2012$exp[Hanrahan201
## W = 56, p-value = 0.003539
## alternative hypothesis: true location shift is not equal to 0
```

```
# NewData: weak vs hetero
# NewData: hetero vs positive
wilcox.test(Hanrahan2012$exp[Hanrahan2012$PTEN == "hetero"],
            Hanrahan2012$exp[Hanrahan2012$PTEN == "weak"])

##
##  Wilcoxon rank sum test
##
## data:  Hanrahan2012$exp[Hanrahan2012$PTEN == "hetero"] and Hanrahan2012$exp[Hanrahan201
## W = 80, p-value = 0.9799
## alternative hypothesis: true location shift is not equal to 0

# Orig Data: Hetero vs positive
wilcox.test(Hanrahan2012$exp[Hanrahan2012$PTEN.TCGA == "hetero"],
            Hanrahan2012$exp[Hanrahan2012$PTEN.TCGA == "pos"])

##
##  Wilcoxon rank sum test
##
## data:  Hanrahan2012$exp[Hanrahan2012$PTEN.TCGA == "hetero"] and Hanrahan2012$exp[Hanrah
## W = 62, p-value = 0.01344
## alternative hypothesis: true location shift is not equal to 0
```

5.5 The Hanrahan 'positive' samples can be resolved into weak, het and pos categories

We can also look at the original 'positive' group and see if there is a difference in expression with the reclassification:

```
idx=which(Hanrahan2012$PTEN.TCGA == "pos")
boxplot(Hanrahan2012$exp[idx] ~ Hanrahan2012$PTEN[idx],ylab="Expression",
        xlab="PTEN classification (Ours)",
        main ="Reclassified 'positive' samples")
```

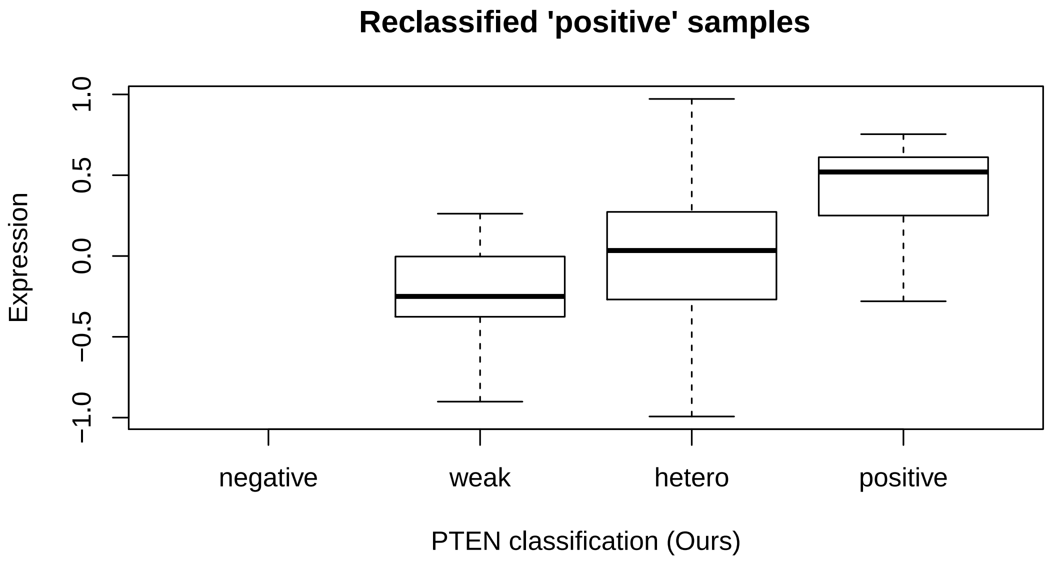


```
## Compare the samples which were previously considered "positive" by Hanrahan with the ne

# new positive vs heterogeneous
wilcox.test(Hanrahan2012$exp[Hanrahan2012$PTEN.TCGA == "pos"
                                  & Hanrahan2012$PTEN=="positive"],
            Hanrahan2012$exp[Hanrahan2012$PTEN.TCGA == "pos"
                                  & Hanrahan2012$PTEN=="hetero"])

##
##  Wilcoxon rank sum test
##
## data:  Hanrahan2012$exp[Hanrahan2012$PTEN.TCGA == "pos" & Hanrahan2012$PTEN ==  and Han
## W = 144, p-value = 0.0325
## alternative hypothesis: true location shift is not equal to 0

# new positive vs weak
wilcox.test(Hanrahan2012$exp[Hanrahan2012$PTEN.TCGA == "pos"
                                  & Hanrahan2012$PTEN=="hetero"],
            Hanrahan2012$exp[Hanrahan2012$PTEN.TCGA == "pos"
                                  & Hanrahan2012$PTEN=="weak"])

##
##  Wilcoxon rank sum test
##
## data:  Hanrahan2012$exp[Hanrahan2012$PTEN.TCGA == "pos" & Hanrahan2012$PTEN ==  and Han
```

```
## W = 58, p-value = 0.3507
## alternative hypothesis: true location shift is not equal to 0
```

We see a marked variation in PTEN gene expression of samples which were labelled as positive by Hanrahan. After deconvolving the positive samples (15 remained positive, 14 became heterogeneous and 7 are now weak), we see a statistical difference between the positive group and the other two groups.

5.6 Correlation between scores for PTEN IHC staining and PTEN CNA [Fig. 3H]

We found that "negative" staining correlated well with homozygous deletion, while "weak" or "heterogeneous" staining correlated well with hemizygous loss and "positive" staining with no chromosomal loss:

```
d <- subset(Hanrahan2012, select = c("PTEN.genotype","PTEN"))
plotcorrImage(d)
#Perform Fisher's Exact test and get p-value
p <- fisher.test(table(na.omit(d)))
title(main=paste("Figure 3H: Correlation between scores for",
     "\nPTEN IHC staining and CNA, p-value=",round(p$p.value,3)),
     cex.main=0.9)
```

Figure 3H: Correlation between scores for PTEN IHC staining and CNA, p−value= 0.001

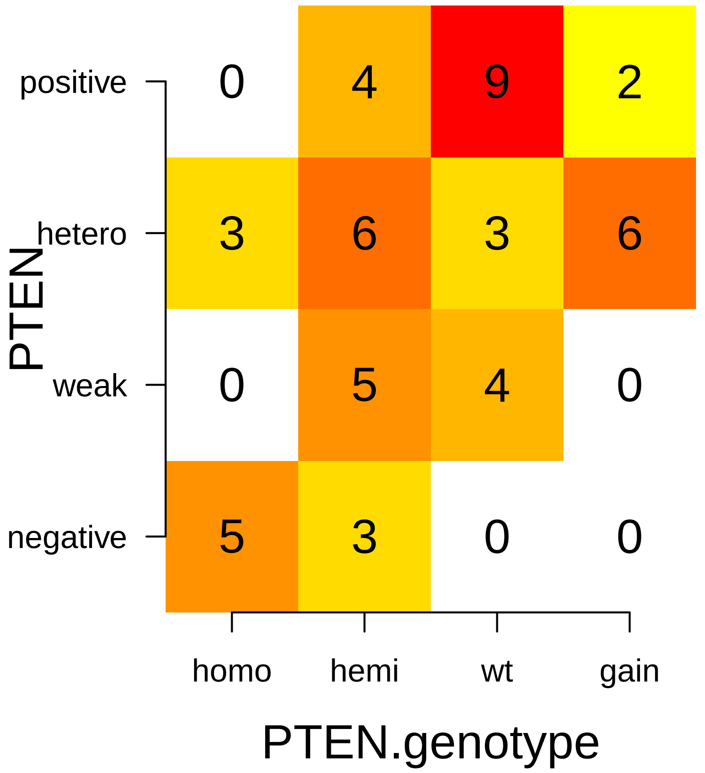

6 Testing the prognostic value of AR and its association with PTEN expression

6.1 Verifying AR as a differentially expressed gene using csSAM

The theory behind csSAM method is defining two known groups (e.g.. tumour vs non-tumour) and inferring the expression of each distinct cell type. The end result is a single expression value for the two groups, on which differential gene analysis is performed. More details can be found in Shen-Orr et al. (2010).

Here, we run csSAM using our original Gene Expression Matrix and our Stromal Fractions estimated from Image Analysis and compare it to results attained from selecting low ACTA2 patients.

```
## y is the group indices
y <- cut(TCGA.GeneExpMat[, "PTEN"], breaks = quantile(TCGA.GeneExpMat[, "PTEN"],
    c(0, 0.25, 0.75, 1)), include.lowest = TRUE, labels = c(1, 3, 2))
## cc is the tumour and stroma content
cc = data.frame(stroma = GoodQualAverage, tumor = 1 - GoodQualAverage)
idx1 = which(y != 3 & !is.na(cc[, 1]))
cc = cc[idx1, ]
y = y[idx1]
summary(y)

##  1  3  2
## 49  0 68

## Run the csSAM wrapper TODO: apply filter to remove zero values to suppress
## non-serious Warnings
deconvResults = csSamWrapper(TCGA.GeneExpMat[idx1, ], cc, y, nperms = 100, alternative = "
    standardize = TRUE, medianCenter = TRUE, fileName = "testout.pdf")

## Warning:  1 x value <= 0 omitted from logarithmic plot
## Warning:  1 x value <= 0 omitted from logarithmic plot
## Warning:  1 x value <= 0 omitted from logarithmic plot

## Note that a pdf is produced with the FDR rate and the number of genes it
## correlates to for each type summary of the results
summary(deconvResults)

##                 Length Class  Mode
## deconv              2  -none- list
## fdr.csSAM           6  -none- list
## fdr.SAM             4  -none- list
## sigGene.csSAM 23728  -none- numeric
## fileName            1  -none- character
```

```r
## Output is a FDR for each gene:
deconvResults$sigGene.csSAM[1:2, 1:5]

##        [,1]   [,2]   [,3]   [,4]   [,5]
## [1,] 0.6513 0.7190 0.8742 0.7190 0.9105
## [2,] 0.0912 0.8613 0.9483 0.5269 0.6354
```

```r
## Select a FDR threshold of interest based on tumour only samples:
GeneIdx = which(deconvResults$sigGene.csSAM[2, ] < 0.2)
SigGenesTumour = colnames(TCGA.GeneExpMat)[GeneIdx]
SigGenesTumour

##   [1] "A2BP1"     "ABCG5"     "ABHD10"    "ADAM28"    "ADAM8"
##   [6] "ANKRD5"    "AR"        "ASRGL1"    "B3GALNT1"  "B4GALNT1"
##  [11] "C14ORF161" "C6ORF105"  "CCL20"     "CCNL1"     "CD46"
##  [16] "CD48"      "CD55"      "CDKN2A"    "CFI"       "CHMP5"
##  [21] "CLGN"      "CLN5"      "COX17"     "COX5B"     "CRABP1"
##  [26] "CXCL1"     "CXCL10"    "CXCL11"    "CXCL13"    "CXCL2"
##  [31] "CXCL3"     "DEFB4"     "DIRAS2"    "DOK5"      "EFHC2"
##  [36] "GABRE"     "GOLT1B"    "GPC5"      "GSR"       "GYG1"
##  [41] "HOXD1"     "HTR2C"     "HTR3A"     "IFI44"     "IFI44L"
##  [46] "IFI6"      "IFNG"      "IL1B"      "IL8"       "ISG15"
##  [51] "ISG20"     "KCNK1"     "KLHDC8A"   "LAG3"      "LAMP3"
##  [56] "LAP3"      "LDOC1"     "LEPROTL1"  "MAD2L1"    "MAGEC2"
##  [61] "MIPEP"     "MKKS"      "MLF1"      "MMP12"     "MPPED2"
##  [66] "MYNN"      "NDUFB4"    "NMD3"      "NOX4"      "NR0B1"
##  [71] "NRGN"      "OAS1"      "OASL"      "P15RS"     "PCBD1"
##  [76] "PCDH21"    "PDZK1"     "PFN2"      "PIGR"      "PLSCR1"
##  [81] "POMP"      "PSMD10"    "PTEN"      "PYROXD1"   "RMI1"
##  [86] "RSAD2"     "RWDD2A"    "SCGB1D2"   "SCGB2A1"   "SERPINB8"
##  [91] "SGCE"      "SIT1"      "SRD5A2L"   "STAT1"     "SUSD4"
##  [96] "TDRD7"     "TMEM165"   "TMEM16A"   "TMEM39A"   "TMEM49"
## [101] "TMPRSS4"   "TNF"       "TNFSF10"   "TXNDC13"   "TXNL4B"
## [106] "UTS2"      "ZNF282"
```

Using a cut-off of 0.2 (which is below the relatively permissive value of 0.3 reported in the publication), we find 107 significant genes, of which AR is one of them.

We then check to see whether the method we have applied correlates with that used in subsetting:

```r
par(mfrow = c(1, 2))

# Can also test the ranking of genes with the Differential Analysis
```

```r
# performed before
GeneTumRank = deconvResults$sigGene.csSAM[2, ]
DE_valsRank = DE_valsAll[order(DE_valsQuart[, 1]), ]
plot(GeneTumRank, log10(DE_valsRank[, 3]), xlab = "csSAM FDR", ylab = "DEGA adjusted p val
    main = "Comparison of Subsetting method with csSAM")
abline(h = log10(0.05), v = 0.2, col = "red")

## Intersect of the two methods used:
DEGenes = rownames(toptable)
venn(list(DEGA = DEGenes, csSAM = SigGenesTumour))
```

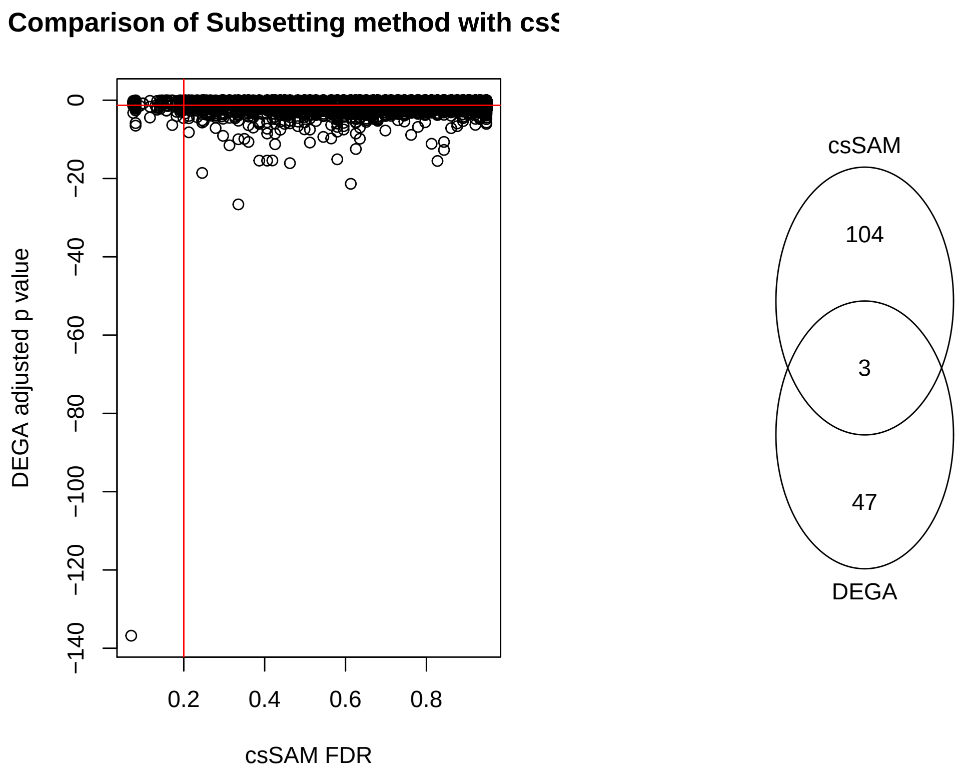


```r
intersect(DEGenes, SigGenesTumour)

## [1] "PTEN"  "AR"    "PCBD1"
```

We first compare the p-values attained from DEGA and the FDRs attained from csSAM. We see a few genes which are significant in both analyses.

Looking at the intersect of these two lists, we see 3, of which both PTEN and AR are present.

6.2 Survival Analysis using data from the TCGA cohort [Fig. 4B]

Using the TCGA RNAseq data, we found that low AR expression was associated with shorter overall survival:

```
data2plot <- merge(TCGA.ClinData, cbiodata, by.x="BCRPATIENTBARCODE",
                   by.y="row.names")
data2plot <- subset(data2plot, select=c("OverallSurvival.mos",
                                        "VITALSTATUS","AR"))
data2plot$ARquart <- cut(as.numeric(as.character(data2plot$AR)),
            breaks=quantile(as.numeric(as.character(data2plot$AR)),
                            c(0, 0.5, 1), na.rm=T),
            labels=c("low", "high"))
ss=Surv(data2plot$OverallSurvival.mos, data2plot$VITALSTATUS)
table(data2plot$ARquart[!is.na(ss)])

##
##  low high
##  129  126

plot(survfit(ss~data2plot$ARquart), col=c("royalblue","red"),
     main="Figure 4B: Survival based on AR RNA-seq", cex.main=.9,
     xlab="Overall survival [months]", ylab="Survival Probability",
     lwd=2)
#add legend: number of patients in each group
legendtext = paste("AR ", levels(data2plot$ARquart),": n = ",
                   table(data2plot$ARquart[!is.na(ss)]),sep="")
legend("topright",col=c("royalblue","red"), legend=legendtext,lty=1)
```

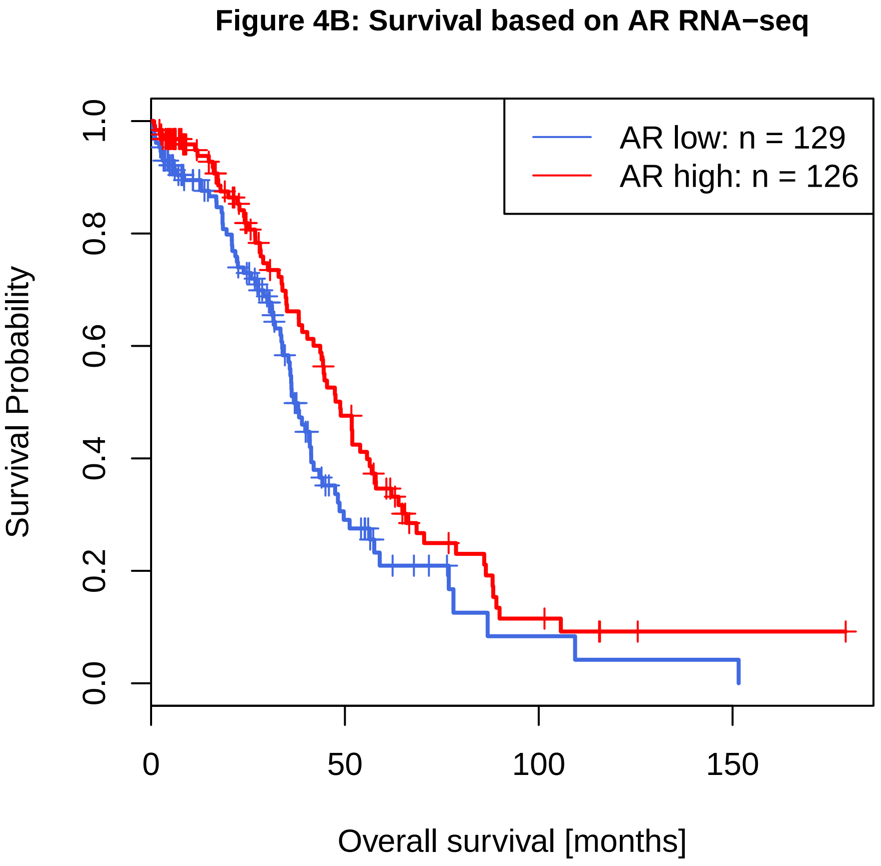


```
survdiff(ss~data2plot$ARquart)

## Call:
## survdiff(formula = ss ~ data2plot$ARquart)
##
## n=255, 234 observations deleted due to missingness.
##
##                            N Observed Expected (O-E)^2/E (O-E)^2/V
## data2plot$ARquart=low  129       74     60.6      2.97      5.33
## data2plot$ARquart=high 126       70     83.4      2.16      5.33
##
##  Chisq= 5.3  on 1 degrees of freedom, p= 0.021

summary(coxph(ss~data2plot$ARquart))

## Call:
```

```
## coxph(formula = ss ~ data2plot$ARquart)
##
##   n= 255, number of events= 144
##    (234 observations deleted due to missingness)
##
##                           coef exp(coef) se(coef)     z Pr(>|z|)
## data2plot$ARquarthigh -0.390     0.677    0.170 -2.29    0.022 *
## ---
## Signif. codes:  0 '***' 0.001 '**' 0.01 '*' 0.05 '.' 0.1 ' ' 1
##
##                         exp(coef) exp(-coef) lower .95 upper .95
## data2plot$ARquarthigh     0.677       1.48     0.485     0.945
##
## Concordance= 0.558  (se = 0.024 )
## Rsquare= 0.02   (max possible= 0.993 )
## Likelihood ratio test= 5.25  on 1 df,   p=0.022
## Wald test            = 5.26  on 1 df,   p=0.0219
## Score (logrank) test = 5.32  on 1 df,   p=0.0211
```

6.3 Validation of the survival analysis using data from the SEARCH cohort [Fig. 4C, 4D]

To validate these results experimentally, we quantified the expression of AR in 216 tumor samples from the SEARCH cohort using IHC staining. The scores for each SEARCH sample can be found in the SEARCH object under `AR.IHC`. 43%, 25% and 32% of HGSC samples expressed high levels of AR (>50% of tumour cells), low levels of AR (<50% of tumour cells) or no AR, respectively:

```
plotcorrImage(subset(SEARCH, HISTOLOGY == "HGSC", select=c("PTEN.IHC","AR.IHC")))
```

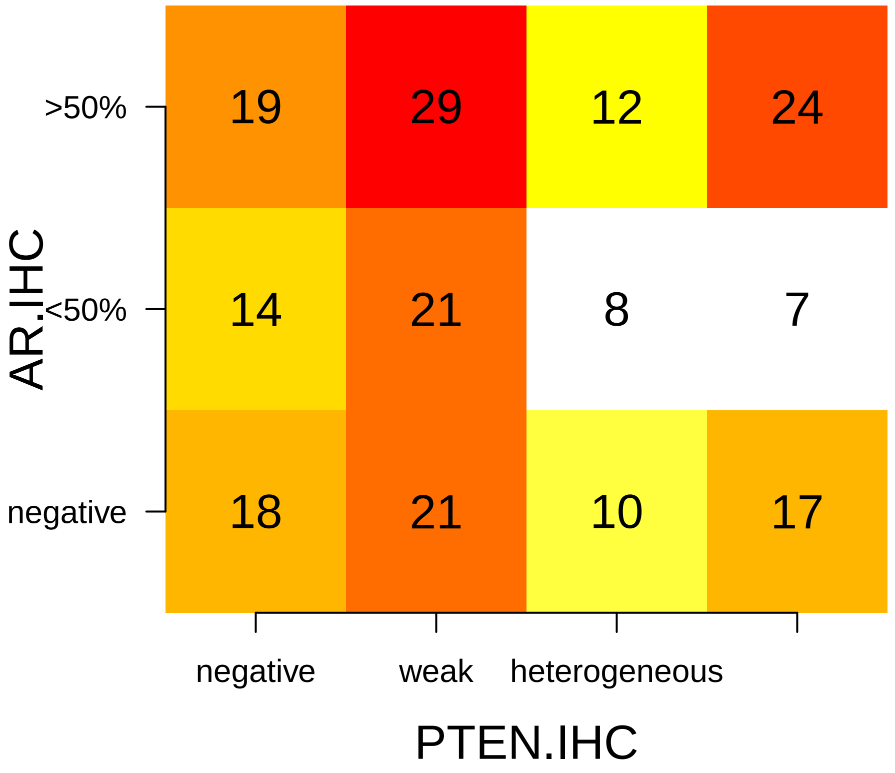

The chi-square test shows no strong correlation between AR and PTEN (p-value = 0.6349):

```
mat <- matrix(table(subset(SEARCH, HISTOLOGY == "HGSC", select = c("PTEN.IHC",
    "AR.IHC"))), nrow = 4, ncol = 3)
mat

##      [,1] [,2] [,3]
## [1,]   18   14   19
## [2,]   21   21   29
## [3,]   10    8   12
## [4,]   17    7   24

chisq.test(mat)

##
##  Pearson's Chi-squared test
```

```
##
## data:  mat
## X-squared = 4.31, df = 6, p-value = 0.6349
```

To investigate whether AR status is correlated with patients' survival we have plotted the Kaplan-Meier estimate of the survivor function for different AR staining scores obtained in HGSCs.

```
#Prepare SEARCH and NOT datasets for the plots below
Ds <- subset(SEARCH, HISTOLOGY == "HGSC")
colors = c("darkblue","lightblue","red")

#Create the survival object
ss1 <- Surv(time=Ds$Months.To.Entry,time2=Ds$Month.To.Status,
           event=Ds$Vital.Status, type="counting")
plot(survfit(ss1 ~ Ds$AR.IHC), col= colors,
     xlab="Overall survival [months]",ylab="Survival Probability",
     lwd=3,las=1,main="SEARCH Cohort (AR.IHC)")
#add legend: number of patients in each group and in brackets the number of deaths
legendtext = paste(levels(Ds$AR.IHC),": ",table(Ds$AR.IHC[!is.na(ss1)]),
                   "(",table(Ds$AR.IHC[!is.na(ss1) & Ds$Vital.Status==1]),")",sep="")
legend("topright",col=colors, legend=legendtext,lty=1)
```

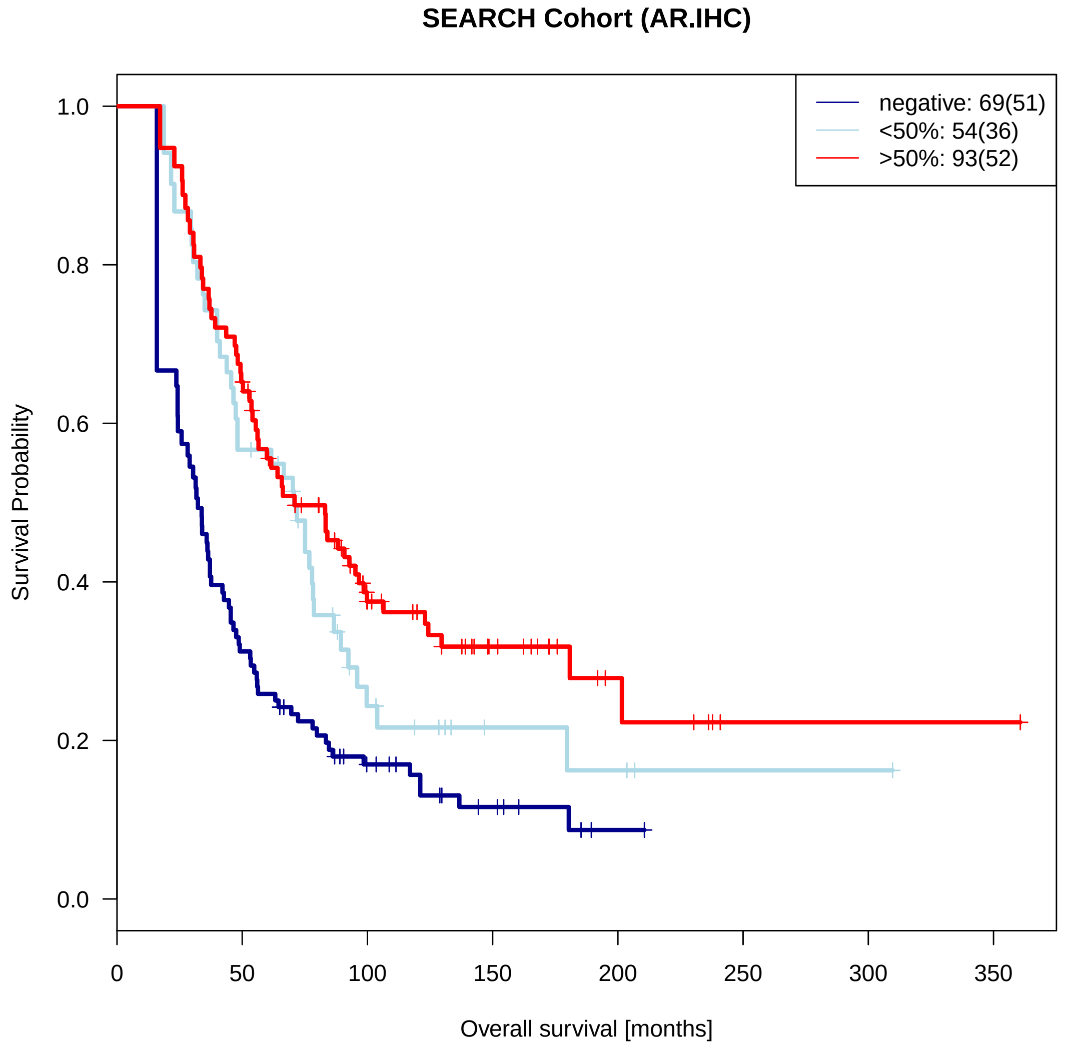

We converted the AR IHC staining scores into "AR high" vs "AR low" (encoded in the `ARstatus` column) and plotted the Kaplan-Meier survival curves. Despite no strong association between AR and PTEN (Chi-square p-value - 0.63), we found a similar prognostic effect of AR expression in the SEARCH cohort:

```
#convert AR scoring into "ARlow", "ARhigh"
labelvect <- c("negative"="ARlow","<50%"="ARhigh", ">50%"="ARhigh")
Ds$ARstatus <- factor(labelvect[Ds$AR.IHC],levels=c("ARlow","ARhigh"))
getsurvival(ss1, Ds$Vital.Status, Ds$ARstatus,title="SEARCH Cohort (AR.IHC)")
```

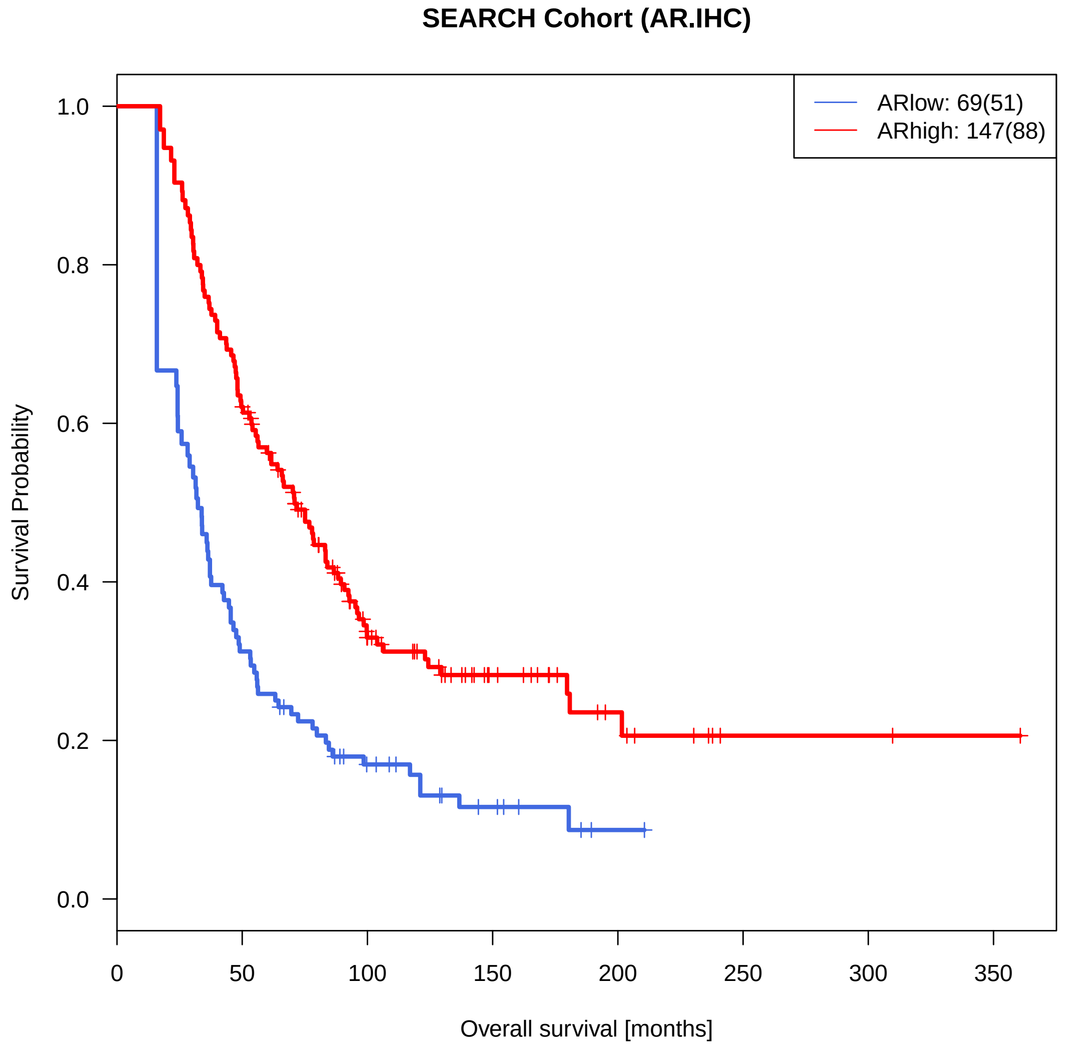


```
## Call:
## coxph(formula = ss ~ predictors)
##
##
##                    coef  exp(coef) se(coef)     z     p
## predictorsARhigh -0.4      0.67    0.177  -2.27 0.023
##
## Likelihood ratio test=4.94  on 1 df, p=0.0263  n= 216, number of events= 139
##    (29 observations deleted due to missingness)
```

The hazard (risk) for "AR high" vs "AR low" tumours was estimated using the Cox-hazard regression model implemented in the function `getsurvival` and correcting for

the patients age, tumour stage and grade.

```
fit0 <- coxph(ss1 ~ Ds$Age.At.Diagnosis + Ds$Grade + Ds$Stage + Ds$ARstatus)
summary(fit0)

## Call:
## coxph(formula = ss1 ~ Ds$Age.At.Diagnosis + Ds$Grade + Ds$Stage +
##     Ds$ARstatus)
##
##   n= 173, number of events= 113
##    (72 observations deleted due to missingness)
##
##                         coef exp(coef) se(coef)     z Pr(>|z|)
## Ds$Age.At.Diagnosis  0.0191    1.0193   0.0129  1.48    0.138
## Ds$Grade            -0.2808    0.7552   0.1975 -1.42    0.155
## Ds$Stage             0.7136    2.0414   0.1246  5.73    1e-08 ***
## Ds$ARstatusARhigh   -0.5010    0.6059   0.1974 -2.54    0.011 *
## ---
## Signif. codes:  0 '***' 0.001 '**' 0.01 '*' 0.05 '.' 0.1 ' ' 1
##
##                     exp(coef) exp(-coef) lower .95 upper .95
## Ds$Age.At.Diagnosis     1.019      0.981     0.994     1.045
## Ds$Grade                0.755      1.324     0.513     1.112
## Ds$Stage                2.041      0.490     1.599     2.606
## Ds$ARstatusARhigh       0.606      1.650     0.412     0.892
##
## Concordance= 0.693  (se = 0.029 )
## Rsquare= 0.246   (max possible= 0.997 )
## Likelihood ratio test= 48.8  on 4 df,   p=6.38e-10
## Wald test            = 43  on 4 df,   p=1.02e-08
## Score (logrank) test = 45.6  on 4 df,   p=2.93e-09

anova(fit0)

## Analysis of Deviance Table
##  Cox model: response is ss1
## Terms added sequentially (first to last)
##
##                     loglik Chisq Df Pr(>|Chi|)
## NULL                  -494
## Ds$Age.At.Diagnosis   -491  5.55  1      0.018 *
## Ds$Grade              -490  1.14  1      0.287
## Ds$Stage              -472 36.02  1      2e-09 ***
## Ds$ARstatus           -469  6.11  1      0.013 *
```

```
## ---
## Signif. codes:  0 '***' 0.001 '**' 0.01 '*' 0.05 '.' 0.1 ' ' 1
```

The AR status obtained with IHC staining is significantly associated with risk of HGSC (p-value = 0.013; HR=1.65)

7 Differentiated and proliferative ovarian cancer subtypes are associated with high and low PTEN expression

In (Verhaak et al., 2013), we find both subgroups assignments (based on gene signatures) and scores for each assignment associated with each tumour, as shown below.

```
grep("ssGSEA", colnames(CLOVARscores), value = TRUE)

## [1] "Differentiated.ssGSEA.raw.score"
## [2] "Immunoreactive.ssGSEA.raw.score"
## [3] "Mesenchymal.ssGSEA.raw.score"
## [4] "Proliferative.ssGSEA.raw.score"
## [5] "Differentiated.ssGSEA.normalized.score"
## [6] "Immunoreactive.ssGSEA.normalized.score"
## [7] "Mesenchymal.ssGSEA.normalized.score"
## [8] "Proliferative.ssGSEA.normalized.score"

summary(factor(PatSubClass.SUBTYPE))

## Differentiated Immunoreactive    Mesenchymal  Proliferative
##            135            107            109            138
```

7.1 Association between HGSC TCGA subtypes and expression of CD3, ACTA2, AR and PTEN in the TCGA dataset [Fig. 5A]

We first evaluated the association between these ssGSEA scores and ACTA2/stromal content and CD3/leucocytes content.

```
par(mfrow=c(2,2), oma = c( 0, 0, 1,3))
plot(Differentiated.ssGSEA.normalized.score, TCGA.GeneExpMat[, "ACTA2"],
     xlab="Diff Score", ylab="ACTA2 expression", col=factor(PatSubClass.SUBTYPE))
plot(Immunoreactive.ssGSEA.normalized.score, TCGA.GeneExpMat[, "ACTA2"],
     xlab="Immuno Score", ylab="ACTA2 expression", col=factor(PatSubClass.SUBTYPE))
plot(Mesenchymal.ssGSEA.normalized.score, TCGA.GeneExpMat[, "ACTA2"],
     xlab="MesScore", ylab="ACTA2 expression", col=factor(PatSubClass.SUBTYPE))
plot(Proliferative.ssGSEA.normalized.score, TCGA.GeneExpMat[, "ACTA2"],
     xlab="Prolif Score", ylab="ACTA2 expression", col=factor(PatSubClass.SUBTYPE))
title("Are ssGSEA scores influenced by stromal content?", outer = TRUE )
```

Are ssGSEA scores influenced by stromal content?

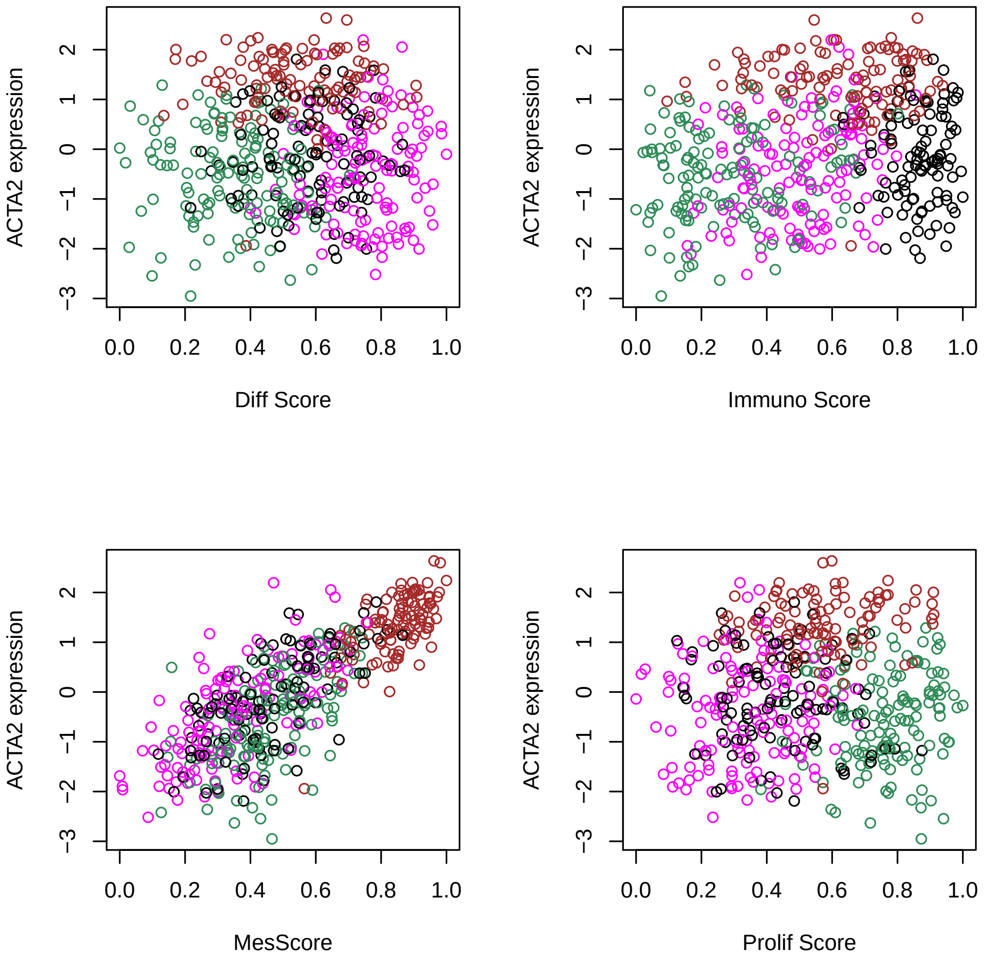


```r
summary(factor(PatSubClass.SUBTYPE))

## Differentiated Immunoreactive    Mesenchymal  Proliferative
##            135            107            109            138

cor.test(Mesenchymal.ssGSEA.normalized.score, TCGA.GeneExpMat[, "ACTA2"])

##
##  Pearson's product-moment correlation
##
## data:  Mesenchymal.ssGSEA.normalized.score and TCGA.GeneExpMat[, "ACTA2"]
## t = 28.67, df = 487, p-value < 2.2e-16
```

```
## alternative hypothesis: true correlation is not equal to 0
## 95 percent confidence interval:
##  0.7570 0.8233
## sample estimates:
##    cor
## 0.7925
```

Here, we see the Proliferative samples in blue, Immunoreactive samples in red, Differentiated samples in black and Mesenchymal in green.

A strong correlation between Mesenchymal score and ACTA2 expression is observed. The subgroups also tend to cluster for the Differentiated, Immunoreactive and Proliferative scores.

Looking at the stromal content from image analysis, we also see a higher median in the Mesenchymal subtype

```
boxplot(GoodQualAverage ~ PatSubClass.SUBTYPE, ylab = "Stromal from Image Analysis")
```

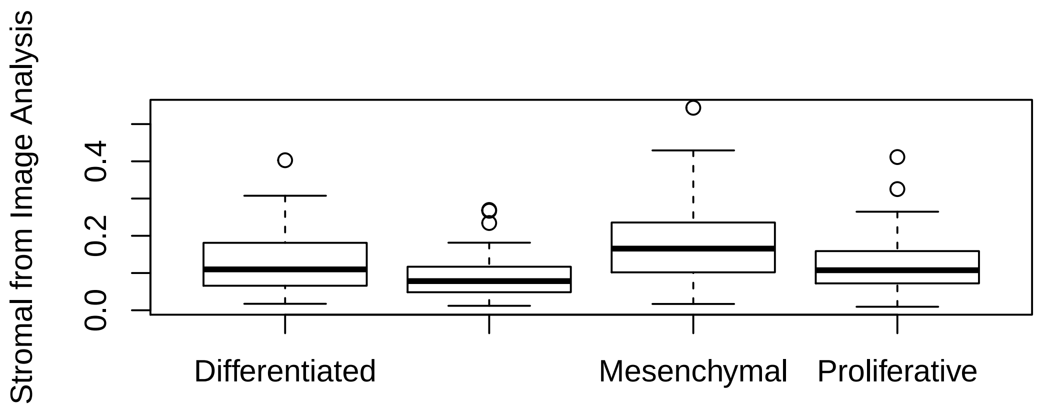


```
## Side clolours:
# CD3: Low (white) --> High (Dark Green)
# ACTA2: Low (white) --> High (Dark Green)
# AR: Low (white) --> High (Dark Blue)
# PTEN: Low (white) --> High (Dark Blue)
# Subtype: Red DIF, Green IMR, Blue MES, Pink PRO

## Determine the side colours
SubtypeCols <- factor(PatSubClass.SUBTYPE)
levels(SubtypeCols) <- list(red = "Differentiated", green = "Immunoreactive",
```

```
                              blue= "Mesenchymal", pink="Proliferative")
SubtypeCols=as.character(SubtypeCols)
GeneCols = sapply(c("PTEN", "AR"), function(x) cut(TCGA.GeneExpMat[ ,x],
                  breaks=quantile(TCGA.GeneExpMat[ ,x], c(0, 0.33, 0.67, 1)),
                  labels=c("white", "lightblue", "blue")))
MarkerCols = sapply(c("ACTA2", "CD3D"), function(x) cut(TCGA.GeneExpMat[ ,x],
                  breaks=quantile(TCGA.GeneExpMat[ ,x], c(0, 0.33, 0.67, 1)),
                  labels=c("white", "lightgreen", "darkgreen")))

##  Plot the heatmap
heatmap.plus(t(as.matrix(TCGA.GeneExpMat[ ,ClovarSig])),
             col=brewer.pal(10, "RdBu"),
             ColSideColors=cbind(SubtypeCols,GeneCols, MarkerCols))
title(main=sprintf("Figure5A: Heatmap of the distribution of
                   \nCD3 and ACTA2 expression with respect
                   \nto CLOVAR subtypes"))
```

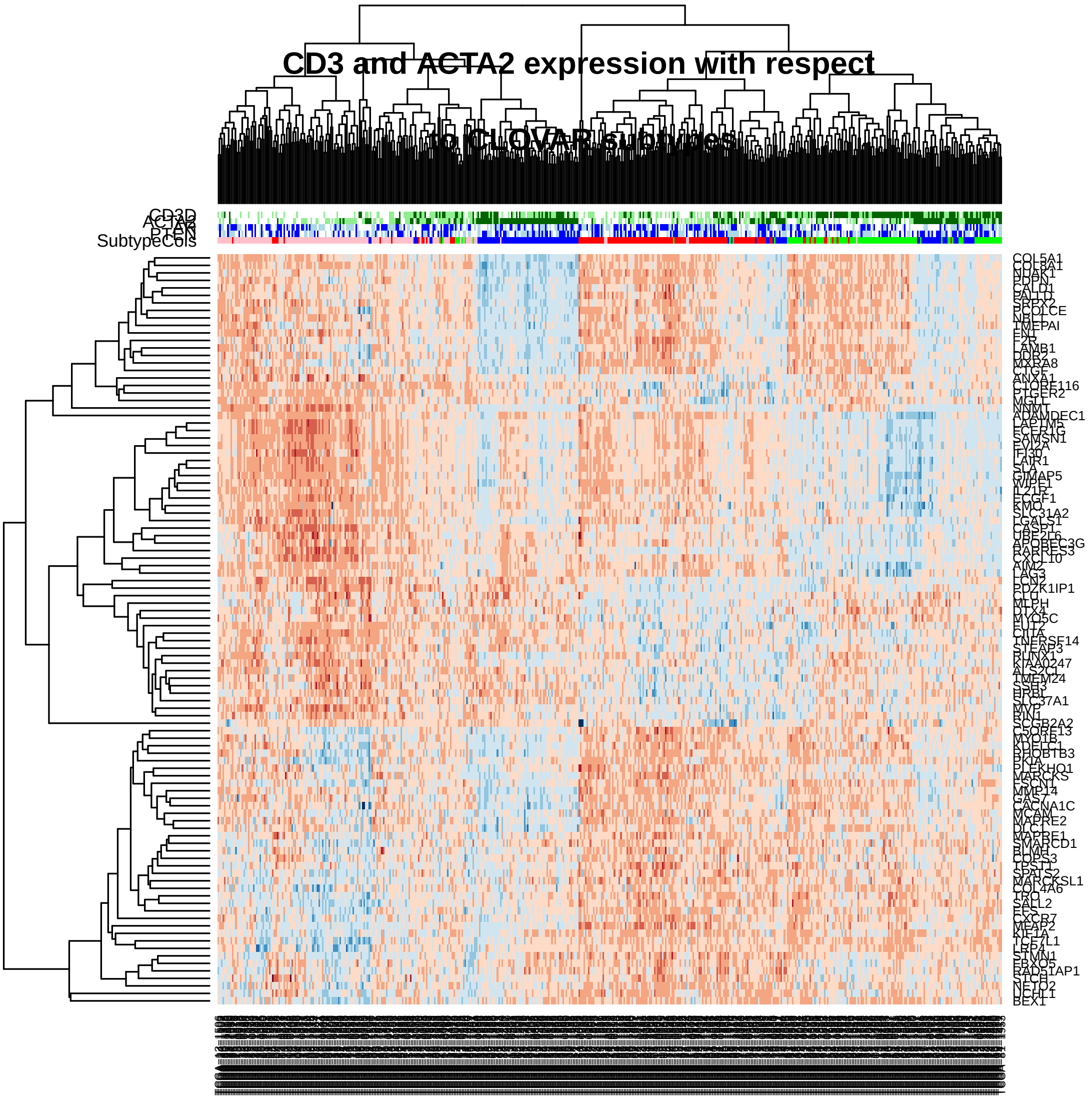

As expected, most of the "Mesenchymal" and "Immunoreactive" subgroups have high ACTA2 and CD3 respectively indicated by the dark and green bars above those given subtypes. Most of the "Proliferative" and "Differentiated" subtypes are shown to have little stromal or immune contamination. Note the cluster attained from this was used for Figure 5A.

We can attempt to omit these subgroups and see if there is a correlation between the Proliferative and Differentiated groups.

```
PDindx = which(PatSubClass.SUBTYPE == "Proliferative" | PatSubClass.SUBTYPE ==
    "Differentiated")
heatmap.plus(t(as.matrix(TCGA.GeneExpMat[PDindx, ClovarSig])), col = brewer.pal(10,
    "RdBu"), ColSideColors = cbind(SubtypeCols[PDindx], GeneCols[PDindx, ],
    MarkerCols[PDindx, ]))
```

```
title(main = sprintf("Figure5A: Heatmap for \n Differentiated and Proliferative subtypes")
```

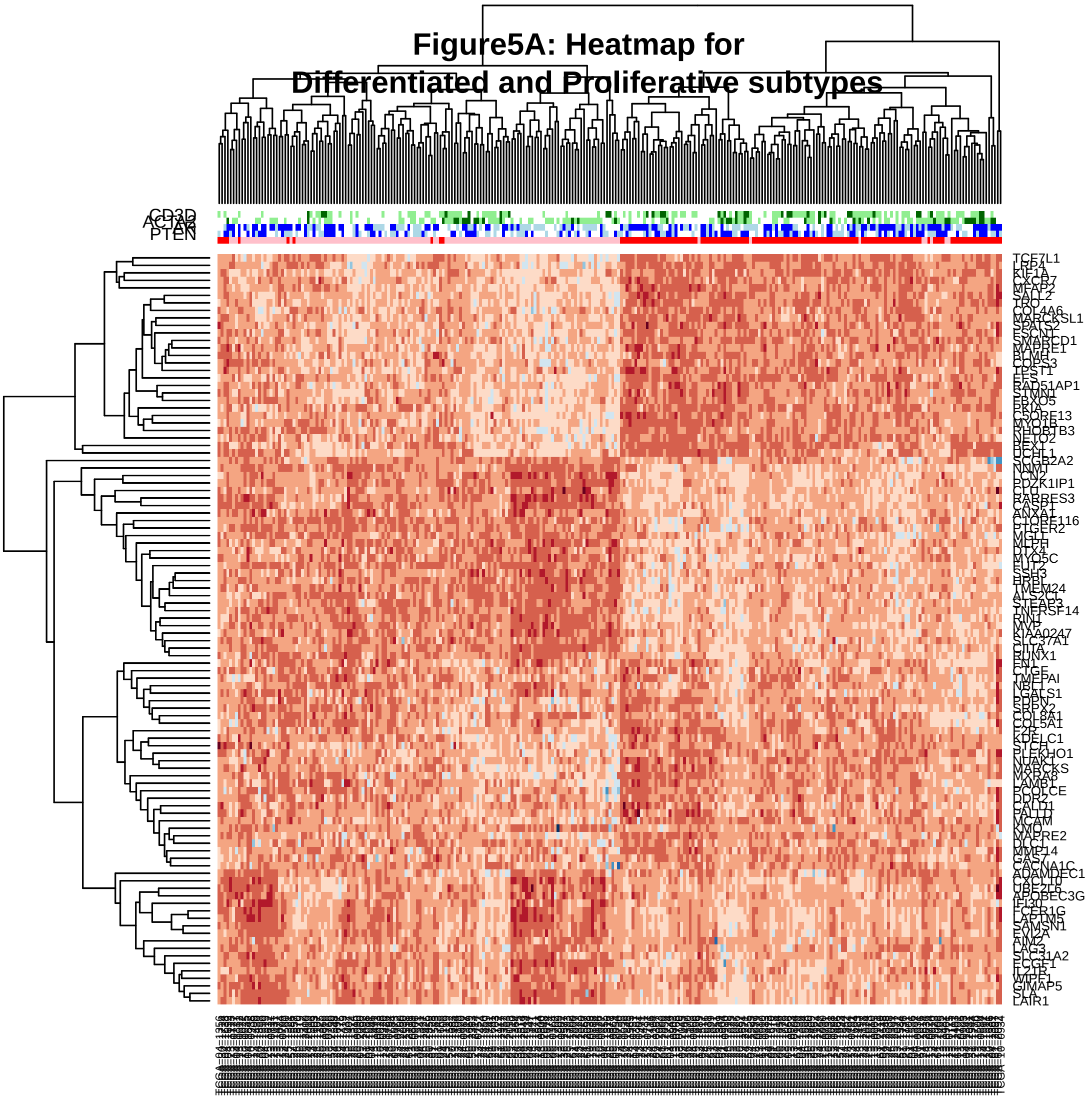

Looking at the column side colours, you can see more blue on the right side than on the left side - suggesting an association of high PTEN and AR with Differentiated samples.

7.2 Association between PTEN expression and the diferentiated and proliferative subtypes [Fig. 5B]

We test whether the previous association is statistically significant with a ChiSquare test, focusing on simply the differentiated and proliferative columns (Figure 5B):

```
PTENtab = data.frame(PatSubClass.SUBTYPE, GeneCols[, 1])
ARtab = data.frame(PatSubClass.SUBTYPE, GeneCols[, 2])
ind1 = which(PTENtab[, 1] == "Differentiated" | PTENtab[, 1] == "Proliferative")

par(mfrow = c(1, 2))

plotcorrImage(PTENtab)
title(main = "Figure 5B:PTEN")
chisq.test(table(PatSubClass.SUBTYPE[ind1], GeneCols[ind1, 1]))

##
##  Pearson's Chi-squared test
##
## data:  table(PatSubClass.SUBTYPE[ind1], GeneCols[ind1, 1])
## X-squared = 7.954, df = 2, p-value = 0.01874

plotcorrImage(ARtab)
title(main = "Figure 5B:AR")
```

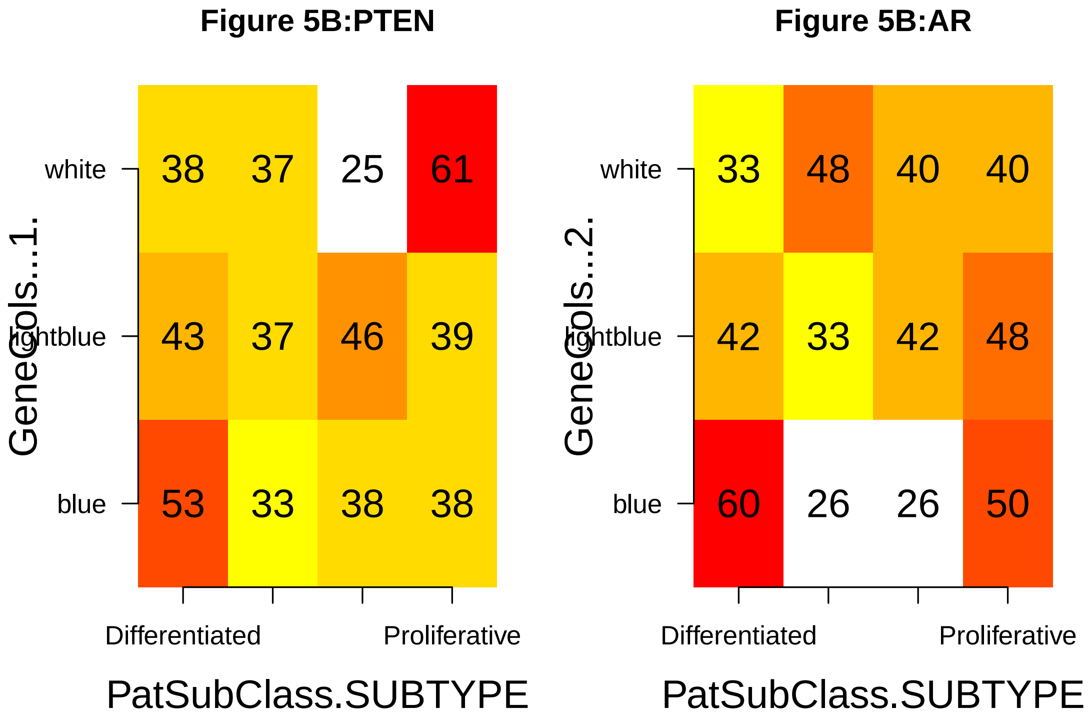

```
chisq.test(table(PatSubClass.SUBTYPE[ind1], GeneCols[ind1, 2]))

##
##  Pearson's Chi-squared test
##
## data:  table(PatSubClass.SUBTYPE[ind1], GeneCols[ind1, 2])
## X-squared = 1.948, df = 2, p-value = 0.3776
```

We clearly see an association between high PTEN and Differentiated subtype and low PTEN and Proliferative subtype. The AR difference however, is not as strong.

8 Session info: R-packages and their versions used for this analysis

```
sessionInfo()

## R version 3.0.2 (2013-09-25)
## Platform: x86_64-apple-darwin10.8.0 (64-bit)
##
## locale:
## [1] en_GB.UTF-8/en_GB.UTF-8/en_GB.UTF-8/C/en_GB.UTF-8/en_GB.UTF-8
##
## attached base packages:
## [1] compiler  splines   stats     graphics  grDevices utils     datasets
## [8] methods   base
##
## other attached packages:
##  [1] quantreg_5.05        SparseM_1.03         scales_0.2.3
##  [4] csSAM_1.2.4          heatmap.plus_1.3     clinfun_1.0.5
##  [7] survival_2.37-6      cluster_1.14.4       made4_1.36.0
## [10] scatterplot3d_0.3-34 gplots_2.12.1        RColorBrewer_1.0-5
## [13] ade4_1.6-2           limma_3.18.9         knitr_1.5
##
## loaded via a namespace (and not attached):
##  [1] bitops_1.0-6       caTools_1.16       colorspace_1.2-4
##  [4] dichromat_2.0-0    digest_0.6.4       evaluate_0.5.1
##  [7] formatR_0.10       gdata_2.13.2       gtools_3.4.0
## [10] highr_0.3          KernSmooth_2.23-10 labeling_0.2
## [13] munsell_0.4.2      mvtnorm_0.9-9997   plyr_1.8
## [16] stringr_0.6.2      tools_3.0.2
```

References

D. A. Barbie, P. Tamayo, J. S. Boehm, S. Y. Kim, S. E. Moody, I. F. Dunn, A. C. Schinzel, P. Sandy, E. Meylan, C. Scholl, S. Fröhling, E. M. Chan, M. L. Sos, K. Michel, C. Mermel, S. J. Silver, B. A. Weir, J. H. Reiling, Q. Sheng, P. B. Gupta, R. C. Wadlow, H. Le, S. Hoersch, B. S. Wittner, S. Ramaswamy, D. M. Livingston, D. M. Sabatini, M. Meyerson, R. K. Thomas, E. S. Lander, J. P. Mesirov, D. E. Root, D. G. Gilliland, T. Jacks, and W. C. Hahn. Systematic RNA interference reveals that oncogenic KRAS-driven cancers require TBK1. *Nature*, 462(7269):108–12, Nov. 2009. ISSN 1476-4687. doi: 10.1038/nature08460. URL `http://www.pubmedcentral.nih.gov/articlerender.fcgi?artid=2783335&tool=pmcentrez&rendertype=abstract`.

A. C. Culhane, J. Thioulouse, G. Perrière, and D. G. Higgins. Made4: an r package for multivariate analysis of gene expression data. *Bioinformatics*, 21(11):2789–2790, Jun 2005. doi: 10.1093/bioinformatics/bti394. URL `http://dx.doi.org/10.1093/bioinformatics/bti394`.

A. Day. *heatmap.plus: Heatmap with more sensible behavior.*, 2012. URL `http://CRAN.R-project.org/package=heatmap.plus`. R package version 1.3.

A. J. Hanrahan, N. Schultz, M. L. Westfal, R. A. Sakr, D. D. Giri, S. Scarperi, M. Janakiraman, M. Janikariman, N. Olvera, E. V. Stevens, Q.-B. She, C. Aghajanian, T. A. King, E. d. Stanchina, D. R. Spriggs, A. Heguy, B. S. Taylor, C. Sander, N. Rosen, D. A. Levine, and D. B. Solit. Genomic complexity and akt dependence in serous ovarian cancer. *Cancer Discov*, 2(1):56–67, Jan 2012. doi: 10.1158/2159-8290.CD-11-0170. URL `http://dx.doi.org/10.1158/2159-8290.CD-11-0170`.

M. Maechler, P. Rousseeuw, A. Struyf, M. Hubert, and K. Hornik. *cluster: Cluster Analysis Basics and Extensions*, 2013. R package version 1.14.4 — For new features, see the 'Changelog' file (in the package source).

F. C. Martins, S. De, V. Almendro, M. Gönen, S. Y. Park, J. L. Blum, W. Herlihy, G. Ethington, S. J. Schnitt, N. Tung, J. E. Garber, K. Fetten, F. Michor, and K. Polyak. Evolutionary pathways in brca1-associated breast tumors. *Cancer Discov*, 2(6):503–511, Jun 2012. doi: 10.1158/2159-8290.CD-11-0325. URL `http://dx.doi.org/10.1158/2159-8290.CD-11-0325`.

E. Neuwirth. *RColorBrewer: ColorBrewer palettes*, 2011. URL `http://CRAN.R-project.org/package=RColorBrewer`. R package version 1.0-5.

N. Otsu. Threshold selection method from gray-level histograms. *IEEE Transations on Systems Man and Cybernetics*, 9:62–66, 1979. ISSN 0018-9472. URL <GotoISI>://A1979GE96000010.

A. C. Ruifrok and D. A. Johnston. Quantification of histochemical staining by color deconvolution. *Anal Quant Cytol Histol*, 23(4):291–299, Aug 2001.

V. E. Seshan. *clinfun: Clinical Trial Design and Data Analysis Functions*, 2013. URL `http://CRAN.R-project.org/package=clinfun`. R package version 1.0.5.

S. S. Shen-Orr, R. Tibshirani, P. Khatri, D. L. Bodian, F. Staedtler, N. M. Perry, T. Hastie, M. M. Sarwal, M. M. Davis, and A. J. Butte. Cell type-specific gene expression differences in complex tissues. *Nat Methods*, 7(4):287–289, Apr 2010. doi: 10.1038/nmeth.1439. URL `http://dx.doi.org/10.1038/nmeth.1439`.

W. Sieh, M. Köbel, T. A. Longacre, D. D. Bowtell, A. deFazio, M. T. Goodman, E. Høgdall, S. Deen, N. Wentzensen, K. B. Moysich, J. D. Brenton, B. A. Clarke, U. Menon, C. B. Gilks, A. Kim, J. Madore, S. Fereday, J. George, L. Galletta, G. Lurie, L. R. Wilkens, M. E. Carney, P. J. Thompson, R. K. Matsuno, S. K. Kjær, A. Jensen, C. Høgdall, K. R. Kalli, B. L. Fridley, G. L. Keeney, R. A. Vierkant, J. M. Cunningham, L. A. Brinton, H. P. Yang, M. E. Sherman, M. García-Closas, J. Lissowska, K. Odunsi, C. Morrison, S. Lele, W. Bshara, L. Sucheston, M. Jimenez-Linan, K. Driver, J. Alsop, M. Mack, V. McGuire, J. H. Rothstein, B. P. Rosen, M. Q. Bernardini, H. Mackay, A. Oza, E. L. Wozniak, E. Benjamin, A. Gentry-Maharaj, S. A. Gayther, A. V. Tinker, L. M. Prentice, C. Chow, M. S. Anglesio, S. E. Johnatty, G. Chenevix-Trench, A. S. Whittemore, P. D. P. Pharoah, E. L. Goode, D. G. Huntsman, and S. J. Ramus. Hormone-receptor expression and ovarian cancer survival: an ovarian tumor tissue analysis consortium study. *Lancet Oncol*, 14(9):853–862, Aug 2013. doi: 10.1016/S1470-2045(13)70253-5. URL `http://dx.doi.org/10.1016/S1470-2045(13)70253-5`.

G. K. Smyth, J. Michaud, and H. S. Scott. Use of within-array replicate spots for assessing differential expression in microarray experiments. *Bioinformatics*, 21(9):2067–2075, May 2005. doi: 10.1093/bioinformatics/bti270. URL `http://dx.doi.org/10.1093/bioinformatics/bti270`.

The Cancer Genome Atlas Network. Comprehensive molecular portraits of human breast tumours. *Nature*, 490(7418):61–70, Oct 2012. doi: 10.1038/nature11412. URL `http://dx.doi.org/10.1038/nature11412`.

R. G. W. Verhaak, P. Tamayo, J.-Y. Yang, D. Hubbard, H. Zhang, C. J. Creighton, S. Fereday, M. Lawrence, S. L. Carter, C. H. Mermel, A. D. Kostic, D. Etemadmoghadam, G. Saksena, K. Cibulskis, S. Duraisamy, K. Levanon, C. Sougnez, A. Tsherniak, S. Gomez, R. Onofrio, S. Gabriel, L. Chin, N. Zhang, P. T. Spellman, Y. Zhang, R. Akbani, K. A. Hoadley, A. Kahn, M. Köbel, D. Huntsman, R. A. Soslow, A. Defazio, M. J. Birrer, J. W. Gray, J. N. Weinstein, D. D. Bowtell, R. Drapkin, J. P. Mesirov, G. Getz, D. A. Levine, M. Meyerson, and C. G. A. R. N. . Prognostically relevant gene signatures of high-grade serous ovarian carcinoma. *J Clin Invest*, 123(1):517–525, Jan 2013.

K. Yoshihara, M. Shahmoradgoli, E. Martínez, R. Vegesna, H. Kim, W. Torres-Garcia, V. Treviño, H. Shen, P. W. Laird, D. A. Levine, S. L. Carter, G. Getz, K. Stemke-Hale, G. B. Mills, and R. G. W. Verhaak. Inferring tumour purity and stromal and

immune cell admixture from expression data. *Nat Commun*, 4:2612, 2013. doi: 10.1038/ncomms3612. URL http://dx.doi.org/10.1038/ncomms3612.
