# Supplementary material for: Combined image and genomic analysis of high-grade serous ovarian cancer reveals PTEN loss as a common driver event and prognostic classifier
Source: Genome Biol. 2014 Dec 17;15(12):526. doi: 10.1186/s13059-014-0526-8 (PMC4268857; doi:10.1186/s13059-014-0526-8)
Supplement: Additional file 5 — Code for image Analysis. Code used to estimate stromal content in TCGA histopathological images. [file 13059_2014_526_MOESM5_ESM.zip › SSHE/readme.pdf]

Stromal Segmentaion in H&E Images

Description of image analysis approach taken

Martins et al 2014

1 Introduction and Preprocessing

This describes the MATLAB scripts used to estimate the stromal content in H&E images from the TCGA. The raw H&E Tissue Slide images files from the TCGA ovarian serous cystadenocarcinoma dataset can be downloaded from `https://tcga-data.nci.nih.gov/tcga/findArchives`.

Each .svs file was segmented into a series of 2000x2000 pixel images using DSStudio. All subimages of size less than 0.8MB were assumed to be mostly blank and discarded from analysis.

Analyses were performed on MATLAB version 7.11 (2010b) with the Image Processing Toolbox.

2 Segmentation of an Image

The code below demonstrates the segmentation process for one subimage. As an example, we can load image '61-2016-01A-BS1-DA69.jpg'. The image output from each step are shown in in Figure 1.

```
 %%Step1.  Load the image of interest
imIn=imread('sampleImages/61-2016-01A-BS1-Da69.jpg');
size(imIn)
imview(imIn)

%%Step2. Intensity Adjustment and Calculation of main tissue Area
[HEIm, HEarea]=HEOutline(imIn);
imview(HEIm)
imview(HEarea)
sum(HEarea(:))
```

The above image was loaded and preprocessed to adjust intensity and mark the main regions of interest. We note that the tissue occupies an area of `3943034` pixels.

We next perform Colour Deconvolution using Ruifrok's method:

```
%% Step3. Perform the Colour Deconvolution
CDIm=ColourDeconvolve(HEIm);
imview(CDIm)
```

Here, we see that most of the cellular regions are present in pink and the stromal regions are blue-green. We next perform a thresholding to segment the stromal region using Otsu's method to determine the optimal value. The user also has an option of providing a value (not run). The resultant overlay image shows the estimated stromal area (green) in the image.

```
%% Step4. Threshold the stromal image
[StrIm, val]=HEThresh(CDIm);
% not run: provide a threshold for the cut-off:
% [StrIm, val]=HEThresh(CDIm, 0.3);
imview(SegArea(imIn, StrIm))
```

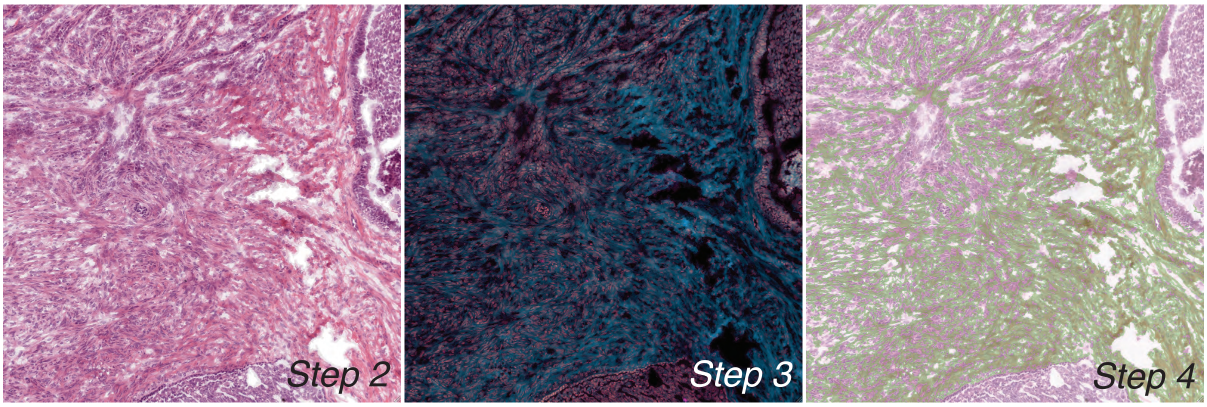

Figure 1: Outputs from segmentation steps. Step2: Image is intensity adjusted and non-tissue regions are set to an intensity of 255. Step3: Colour Deconvolution. Intensities are mapped to OD space and deconvolution is performed. Cells are shown in pink and stroma in blue. Step4: Overlay of the stromal area (green) on the original image.

An example output from the low-stromal sample ('10-0928-01A-BS1-Da123.jpg') is shown in Figure 2

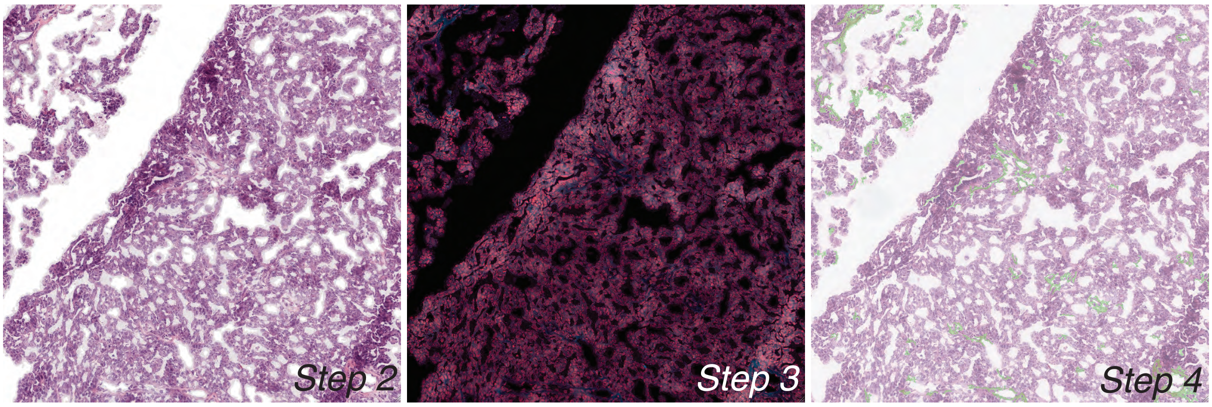

Figure 2: Stromal Segmentation of sample 10-0928, a low stroma sample.

3 Calculating Parameters

The main parameters attained from this analysis are the Tissue Area, Stromal Area and their relative ratios. As multiple subimages are mapped to the same tissue slide, raw tissue and stromal areas were stored. The final stromal fraction for one particular slide is calculated after summing all tissue areas and stromal areas for a particular slide.

```
%% Step5. Calculations:
% Tissue Area:
TArea=sum(HEarea(:))
% Stromal Area:
SArea=sum(StrIm(:))
% Stromal Fraction
SArea/TArea
```

For this particular sample, the stromal fraction is `0.5170`
